# α-KG conformationally stabilizes nuclear GLUD1-SNAIL complex to fuel trans-endothelial migration in metastasis

**DOI:** 10.1038/s41467-026-77149-0

**Published:** 2026-08-31

**Authors:** Jiawen Cui, Nuo Xu, Rongyu Cao, Ruqi Zhang, Ying Yu, Luyao Ao, Feiyi Wang, Qixing Gong, Le Zhen, Yantong Li, Qingqing Zhou, Yue Dang, Guangji Wang, Fang Zhou, Jiali Liu

**Affiliations:** 1 State Key Laboratory of Natural Medicines, China Pharmaceutical University, Nanjing, China; 2 Laboratory of Drug Metabolism and Pharmacokinetics, China Pharmaceutical University, Nanjing, China; 3 Department of Pharmacy, The Affiliated Taizhou People’s Hospital of Nanjing Medical University, Taizhou, China; 4 Department of Pathology, The First Affiliated Hospital of Nanjing Medical University, Nanjing, China; 5 Research Unit of PK-PD Based Bioactive Components and Pharmacodynamic Target Discovery of Natural Medicine of Chinese Academy of Medical Sciences, Nanjing, China; 6 Key Laboratory of Traditional Chinese Medicine Compound Pharmacokinetics of the State Administration of Traditional Chinese Medicine, Nanjing, China; 7 Targeting Discovery and Validation Center, China Pharmaceutical University, Nanjing, China

**Keywords:** Metastasis, Enzyme mechanisms

## Abstract

Trans-endothelial migration (TEM) is a critical rate-limiting barrier in tumor metastasis, yet its mechanistic basis remains poorly defined. Here we show that α-ketoglutarate (α-KG) and its metabolic enzyme glutamate dehydrogenase 1 (GLUD1) constitute a pivotal regulatory axis governing this process. Mechanistically, GLUD1 translocates to the nucleus, where α-KG binds to its non-catalytic site (K147) to induce a conformational change that stabilizes the GLUD1-SNAIL interaction, promoting assembly of a nuclear ternary complex. Notably, this complex is preferentially enriched in tumors, and specific blockade of K147 disrupts this assembly and abrogates metastatic effects. Our findings reveal a non-canonical role for GLUD1 and α-KG in transcriptional regulation and propose a therapeutic paradigm that selectively interferes with metabolite-dependent complex formation rather than enzymatic activity, offering a precise strategy to target TEM-driven metastatic cells.

## Abstract (web summary)

Trans-endothelial migration (TEM) is an important step in cancer metastasis. Here, the authors show that α-ketoglutarate stabilises a nuclear GLUD1-SNAIL complex, facilitating TEM during tumour dissemination.

## Introduction

Intravasation (cross the endothelial barrier from the primary tissue into the circulation) and extravasation (cross the endothelial barrier from circulation into distant tissues) of tumor cells represent the decisive physical barrier during hematogenous dissemination^1–3^. Although intravasation and extravasation may involve distinct regulatory mechanisms due to microenvironmental differences, it would have significant therapeutic implications to identify shared functional mechanisms that could serve as common intervention points. Tumor trans-endothelial migration (TEM) is a common feature of intravasation and extravasation, yet TEM remains one of the least mechanistically defined steps of metastasis^4–7^. While the importance of metabolic rewiring in metastatic competent tumor cells is well established^8–10^, the mechanisms by which this process influences the regulation of TEM are largely unknown.

Glutamate dehydrogenase 1 (GLUD1), a key enzyme that converts glutamate to α-ketoglutarate (α-KG) during glutamine catabolism^11^, is commonly upregulated in aggressive human cancers^12^. GLUD1’s metabolic functions, including generating α-KG to replenish tricarboxylic acid cycle^13^ and maintain redox homeostasis^14^, also generating ammonia to support biomass synthesis^15^, have been well documented in tumor growth. Recently, a compelling concept is emerging wherein tumor cells co-opt metabolic enzymes and metabolites for non-canonical, or “moonlighting” functions. Glycolytic enzymes have been shown to translocate into the nucleus and regulate gene expression through kinase activity, transcription factor interactions, or modulation of transcriptional co-regulators^16^. Recently, GLUD1 was reported to promote hepatitis B virus transcription with KDM4A through modulating histone methylation in the nucleus^17^; however, the underlying molecular mechanisms of nuclear translocation of GLUD1 and whether it fuels metastatically competent tumors beyond its established metabolic activities remain insufficiently understood.

Here, through multi-cohort clinical databases screening and phenotypic validation, we identified GLUD1 as a critical driver of tumor cell TEM. We further uncovered a previously unrecognized mechanism wherein GLUD1 translocates into the nucleus, where its metabolite α-KG functions as a conformational stabilizer, inducing changes in GLUD1 structure that enhance its interaction with the transcription factor SNAIL. This interaction promotes the assembly of a nuclear ternary complex, α-KG/GLUD1/SNAIL, which directly binds to the promoter of *CDH5*, the gene encoding VE-Cadherin, and activates its transcription in tumor cells. This transcriptional upregulation enhances tumor cell-endothelial cell interactions and ultimately promotes TEM during both intravasation and extravasation.

## Results

### GLUD1 was identified as a key mediator of TEM during both intravasation and extravasation

**Fig. 1:**
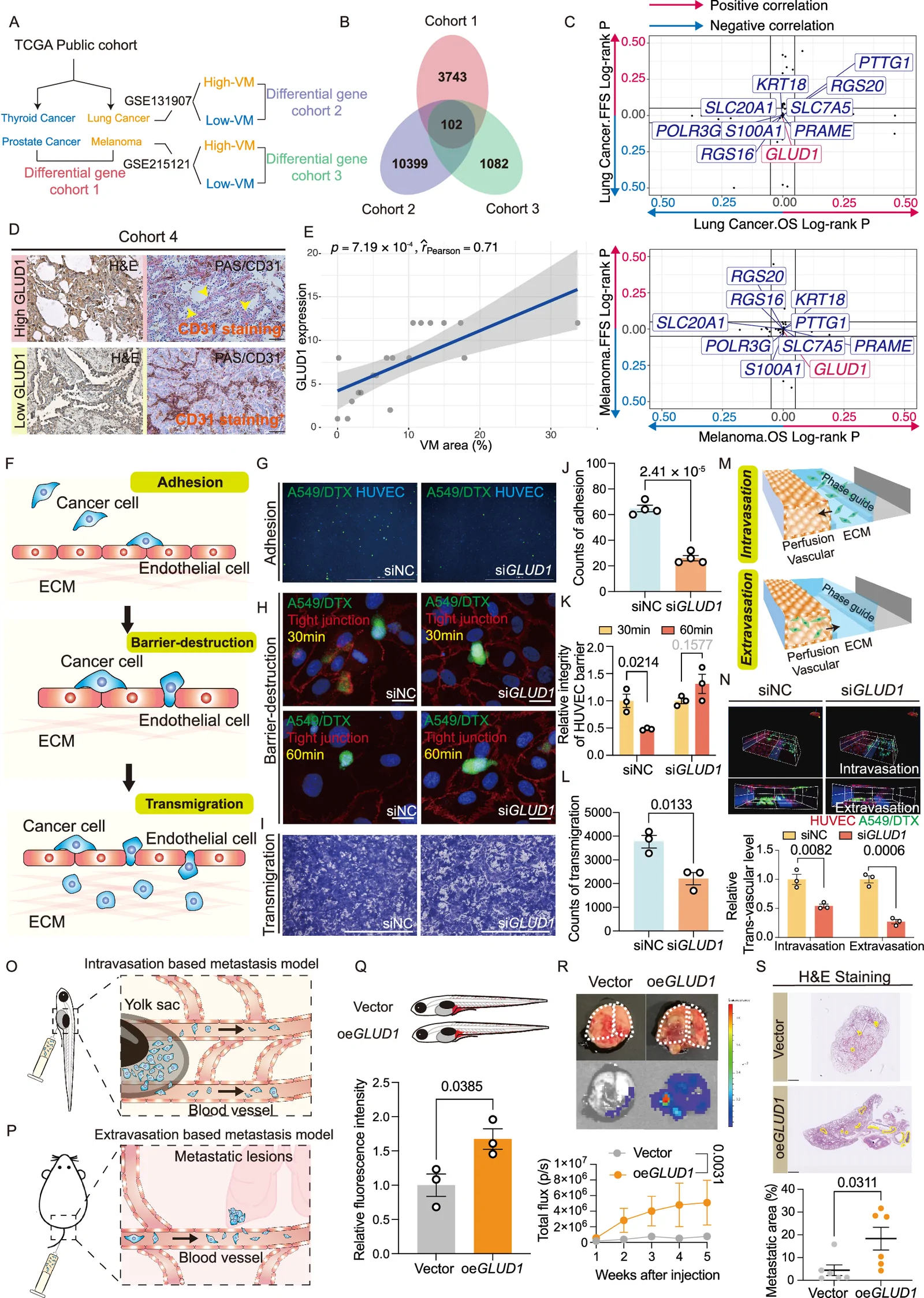
GLUD1 was identified as a key mediator of TEM. **A** Workflow of bioinformatic analyses using TCGA and GEO database. **B** Venn diagram identifying 102 VM-associated genes upregulated across three public cohorts. **C** Comparison of the survival associations of 102 VM-associated genes in lung cancer (top) and melanoma (bottom). The 10 labelled genes exhibited significant negative associations with both overall survival (OS) and failure-free survival (FFS) in both cancer types. Survival analyses included *n* = 589 patients with lung cancer and *n* = 476 patients with melanoma. **D** Representative images of GLUD1 expression and VM area (PAS^+^/CD31^−^) in lung cancer clinical specimens (*n* = 19). **E** Correlation between GLUD1 expression and VM levels in 19 clinical patients’ foci samples. GLUD1 expression is quantified by whole-slide IHC and VM levels are mean values of five PAS^+^/CD31^−^ field of view under ×20 scope. **F** Schematic of TEM: adhesion, barrier-destruction, and transmigration. **G**–**L** Representative immunofluorescence images (**G**–**I**) and quantification (**J**, **K**) of adhesion (**G**, **J**, *n* = 4 biologically independent samples), barrier-destruction (**H**, **K**, *n* = 3 biologically independent samples) and transmigration (**I**, **L**, *n* = 3 biologically independent samples) for siNC or si*GLUD1*-treated A549/DTX cells. Cells in (**G**, **I**) were counted using Fiji. **H** fluorescence was quantified using Fiji and normalized to the 30-min value within each experiment. **M**. Sketch map of intravasation and extravasation using 3D tubular vascular model. **N** Representative images and quantification of intra/extravasation of siNC or si*GLUD1*-treated A549/DTX, *n* = 3 biologically independent samples. All data were normalized to siNC group. **O**, **P** Schematics of the zebrafish intravasation model **O** and murine extravasation model **P**. **Q** Relative quantification of fluorescence intensity of migrating GLUD1-modulated A549/DTX cells in the zebrafish model (representative images top, quantification bottom; *n* = 3 embryo cohorts). **R** Metastatic burden in mice injected with *GLUD1*-overexpressing A549 cells by bioluminescence imaging (top) and weekly quantification (bottom) (*n* = 6 mice). **S** H&E staining and quantification of metastatic burden of lung metastases from (**R**). Metastatic burden was calculated as metastatic area divided by total lung-tissue area. All data are mean ± SEM. *P* values were determined using two-sided Pearson correlation analysis **E**; two-tailed unpaired Student’s *t* test (**J**, **L**, **N**, **Q**, **S**); two-way ANOVA followed by Šídák multiple-comparisons test **K**; two-way ANOVA **R**. Scale bars: D, 100 μm; **G**, **I**, **S**, 1000 μm; **H**, 20 μm. Source data are provided as a Source Data file.

Recent studies have revealed a interesting phenomenon in which tumor cells enhance heterotypic interactions by acquiring features of other cell lineages, thereby promoting cancer progression. For instance, tumor cells can adopt neuronal features to strengthen interactions with central neurons^18,19^, or acquire endothelial-like properties to facilitate interactions with vascular endothelial cells^20,21^. Given that trans-endothelial migration (TEM) fundamentally depends on tumor-endothelial cell interactions, we hypothesized that tumor cells exhibiting vasculature-like features possess an enhanced TEM capacity. This phenomenon, in which tumor cells adopt an endothelial-like identity, is commonly referred to as vasculogenic mimicry (VM)^22–26^. Thus, we isolated high VM, highly metastatic tumor cell subpopulations and analyzed their differentially expressed proteins to identify key mediators of TEM (Fig. 1A). Integration of the MetMap database (Supplementary Fig. 1A) distinguished strongly metastatic (lung cancer, melanoma) from weakly metastatic tumors (thyroid cancer, prostate cancer). VM markers were enriched in aggressive tumors in TCGA public database, supporting the positive correlation of vasculature-like signatures with metastasis^26,27^ (Supplementary Fig. 1B). Variance analysis revealed 3,743 differentially expressed genes between highly and weakly metastatic groups, designated Cohort 1 (Fig. 1B). Furthermore, additional single-cell RNA-seq data from lung cancer and melanoma, stratifies tumor cells into high-VM and low-VM subgroups based on VM programmers *SERPINE2/SLPI* expression^26^. Comparative analysis yielded two additional gene sets (Cohorts 2 and 3). Intersection of all three cohorts identified 102 genes (Fig. 1B). Among these 102 genes, only 10 genes exhibited concurrent significant negative correlations with both overall survival (OS) and failure-free survival (FFS). *GLUD1* emerged as the lead candidate, notably the only endogenous metabolic enzyme identified among the hits (Fig. 1C). Clinical validation in 19 lung cancer specimens (cohort 4) confirmed that GLUD1 expression strongly correlated with VM intensity (Fig. 1D, E). Consistent with clinical observations, lung cancer cell lines with higher GLUD1 expression exhibited enhanced in vitro VM phenotype, as assessed by the tube formation assay, a widely used functional in vitro surrogate of VM capacity evaluation (Supplementary Fig. 1C). This VM phenotype was abolished by *GLUD1* knockdown and augmented by its overexpression (Supplementary Fig. 1D, E).

Although the role of VM in tumor-endothelial heterotypic interactions has been proposed^28^, and we identified GLUD1 from highly metastatic, VM-positive tumor subpopulations, whether GLUD1 mediates TEM remains unclear. Therefore, we established both in vitro and in vivo experimental systems to address this question. TEM consists of three dynamic phases: adhesion, barrier disruption, and transmigration. We first assessed GLUD1 function using a three-phase co-culture model that recapitulates these steps (Fig. 1F). A549/DTX cells (high-GLUD1/high-VM) outperformed parental A549 cells (low-GLUD1/low-VM) in all phases (Supplementary Fig. 1F–K). This GLUD1-dependent regulation was confirmed through *GLUD1* knockdown in A549/DTX cells, which dramatically impaired all three TEM phases (Fig. 1G–L and Supplementary Fig. 1L–Q, showing results from two distinct siRNA sequences). The essential role of GLUD1 in mediating heterotypic interactions was further validated in PC9/OR cells, another established high-GLUD1/high-VM model system (Supplementary Fig. 1R–W). Further, to determine whether GLUD1-mediated TEM contributes to both intravasation and extravasation, we developed a reliable 3D vascular system that reconstructs key structural and barrier features of native vasculature, including physiological tube-like architecture and tight endothelial barriers (Supplementary Fig. 2A–C). This system exhibited a permeability coefficient (Papp) of 10^−7 ^cm/s comparable to that of real blood vessels (Supplementary Fig. 2D, E). Using this model, we simulated both intravasation and extravasation by seeding tumor cells either within the extracellular matrix (for intravasation) and directly into the vascular lumen (for extravasation) (Fig. 1M). Notably, *GLUD1* knockdown attenuated both intravasation and extravasation (Fig. 1N).

To more rigorously validate the role of GLUD1-mediated TEM in both intravasation and extravasation, we utilized two distinct in vivo metastasis models that rely on intravasation or extravasation. In the zebrafish yolk sac injection model, tumor cells form a confined aggregate within the yolk sac that serves as a surrogate primary tumor, from which they intravasate into the bloodstream^29–32^. In the tail vein injection model, tumor cells are administered directly into the circulation and subsequently extravasate to form metastatic lesions in the absence of primary tumor^33–35^ (Fig. 1O, P). Pan-cell line analysis demonstrated that high-GLUD1/high-VM cells exhibited greater metastatic dissemination in intravasation based zebrafish models (Supplementary Fig. 2F-2G) and formed larger and more metastatic lesions in extravasation based murine models (Supplementary Fig. 2H–L). Notably, these lesions displayed elevated PAS-positive/CD31-negative (PAS⁺/CD31⁻) staining, the histopathological gold standard for identifying endothelium-independent vasculogenic mimicry in vivo. (Supplementary Fig. 2M, N). Conversely, overexpression of *GLUD1* in low-GLUD1/low-VM cell lines significantly enhanced metastatic burden in both intravasation based zebrafish models (Fig. 1Q) and extravasation based murine models (Fig. 1R, S and Supplementary Fig. 2O, P), accompanied by upregulated PAS^+^/CD31^−^ staining (Supplementary Fig. 2Q). Furthermore, given that implanting cancer cells into the intervitelline space represents a more accurate approach that permits both invasion and intravasation^36,37^, we performed additional experiments using this model. The results showed that GLUD1 similarly regulated tumor cell dissemination in the intervitelline space injection model, consistent with the findings from the yolk sac injection approach (Supplementary Fig. 2R).

Collectively, GLUD1 was selectively identified from high-VM and highly metastatic tumor subpopulations and functionally validated as a key mediator of TEM, influencing both tumor cell intravasation and extravasation.

### GLUD1 promotes TEM by regulating VE-Cadherin

**Fig. 2:**
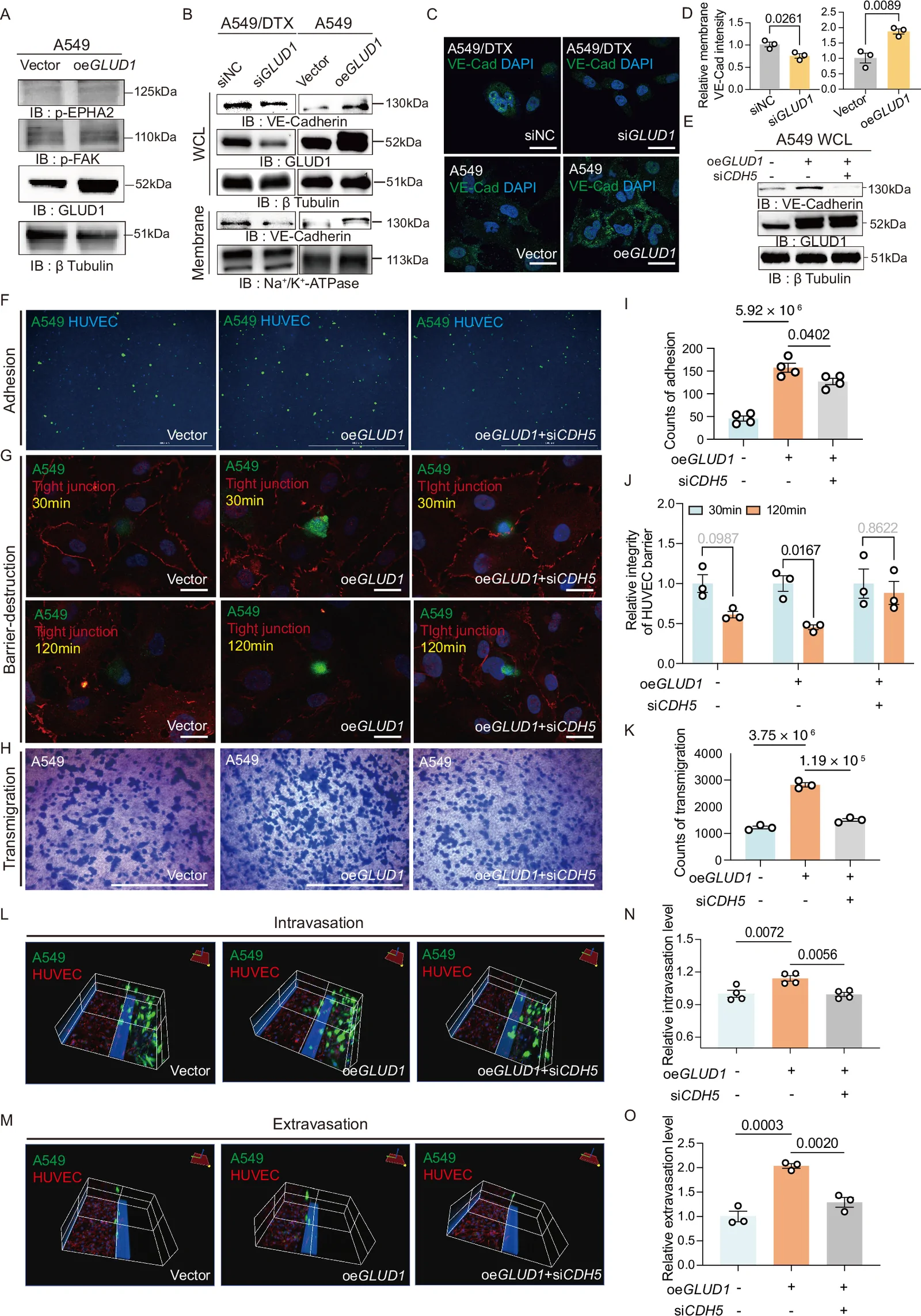
GLUD1 promotes TEM by regulating VE-Cadherin. **A** Representative immunoblotting analysis (*n* = 3 independent experiments) of phosphorylated EPHA2 and FAK (EPHA2 Y588 and FAK Y397) in A549 (oe*GLUD1* versus vector). **B** Representative immunoblotting analysis (*n* = 3 independent experiments) of VE-Cadherin protein expression in whole-cell lysates (WCL) and membrane fractions of A549/DTX (si*GLUD1* versus siNC; left) and A549 (oe*GLUD1* versus vector; right). **C** Immunofluorescence staining of VE-Cadherin in A549/DTX (si*GLUD1* versus siNC; top) and A549 (oe*GLUD1* versus vector; bottom), with quantification shown in (**D**), *n* = 3 biologically independent samples. The average fluorescence intensity of VE-Cadherin on the cell membrane surface was calculated by manually selecting the area using FIJI. **E** Representative immunoblotting analysis (*n* = 3 independent experiments) of VE-Cadherin and GLUD1 in A549 WCL following *GLUD1* overexpression and subsequent *CDH5* knockdown. **F**–**K** Representative images (**F**–**H**) and quantification (**I**–**K**) of adhesion (**F**, **I**, *n* = 4 biologically independent samples), barrier-destruction (**G**, **J**, *n* = 3 biologically independent samples) and transmigration (**H**, **K**, *n* = 3 biologically independent samples) for siNC or si*GLUD1*-treated A549 cells. Cells in (**F**, **H**) were counted using Fiji. **G** fluorescence was quantified using Fiji and normalized to the 30-min value within each experiment. **L**–**O** Immunofluorescence analysis of intravasation (**L**) and extravasation (**M**) in A549 cells following *GLUD1* overexpression and subsequent *CDH5* knockdown. Intravasation was quantified by tumor cells’ average fluorescence intensity in adjacent channels (*N*, *n* = 4 biologically independent samples), while extravasation was measured by ECM-invading cells (**O**, *n* = 3 biologically independent samples). All data were normalized to oe*GLUD1*^−^/si*CDH5*^−^ group. All data are represented as mean ± SEM. *P* values were calculated using two-tailed unpaired Student’s *t* test **D**; one-way ANOVA followed by Dunnett’s multiple-comparisons test (**I**, **K**, **N**, **O**); two-way ANOVA followed by Šídák multiple-comparisons test **J**. Scale bars: **C**, **G**, 20 μm; **F**, **H**, 1000 μm. Source data are provided as a Source Data file. For immunoblots, samples within each panel were derived from the same experiment, with separate gels processed in parallel where applicable; detailed gel assignments are provided in the Methods.

VE-Cadherin, EPHA2 and FAK have been implicated as key mediators of VM and invasive behavior in aggressive tumors. Aberrant VE-Cadherin expression promotes tumor cell plasticity and its interaction with vascular endothelial cells. EPHA2 and its phosphorylation-dependent activation cooperate with VE-Cadherin to activate downstream signaling pathways that facilitate motility and matrix remodeling while FAK and its phosphorylation-dependent activation contribute to cytoskeletal dynamics and invasive phenotypes associated with VM^21,38^. Given that GLUD1 mediates TEM, we first asked whether it acts through these known regulators. Genetic overexpression of *GLUD1* in low-GLUD1/low-VM cell lines failed to influence the protein abundance of total and phosphorylated FAK and EPHA2 (Fig. 2A and Supplementary Fig. 3A).

VE-Cadherin, a key mediator of tumor-endothelial heterotypic interactions whose knockdown significantly impaired all phases of TEM (Supplementary Fig. 3B–G), was consistently associated with GLUD1 expression in isolated mouse metastatic lesions (Supplementary Fig. 3H, I). Furthermore, A549/DTX and PC9/OR cells derived from their parental counterparts exhibit elevated GLUD1 expression. Notably, high GLUD1 expression in these cells correlated with increased VE-Cadherin protein levels (Supplementary Fig. 3J) and membrane-localized levels (Supplementary Fig. 3K, L). Knockdown of GLUD1 in A549/DTX and PC9/OR cells markedly reduced VE-Cadherin expression across these levels, whereas *GLUD1* overexpression in A549 and PC9 cells significantly upregulated it (Fig. 2B–D and Supplementary Fig. 3M–O). These findings suggest that GLUD1 mediates the protein expression of VE-Cadherin. Crucially, *CDH5* silencing largely abolished GLUD1-driven adhesion, barrier destruction, and transmigration in 2D co-culture systems (Fig. 2E–K), as well as intravasation and extravasation in 3D organoid models (Fig. 2L–O). Conversely, *CDH5* overexpression rescued the impaired TEM phenotypes caused by *GLUD1* silencing (Supplementary Fig. 3P–U), confirming that VE-Cadherin is essential for GLUD1-mediated TEM.

### GLUD1 activates VE-Cadherin transcription by forming a nucleus complex with SNAIL

**Fig. 3:**
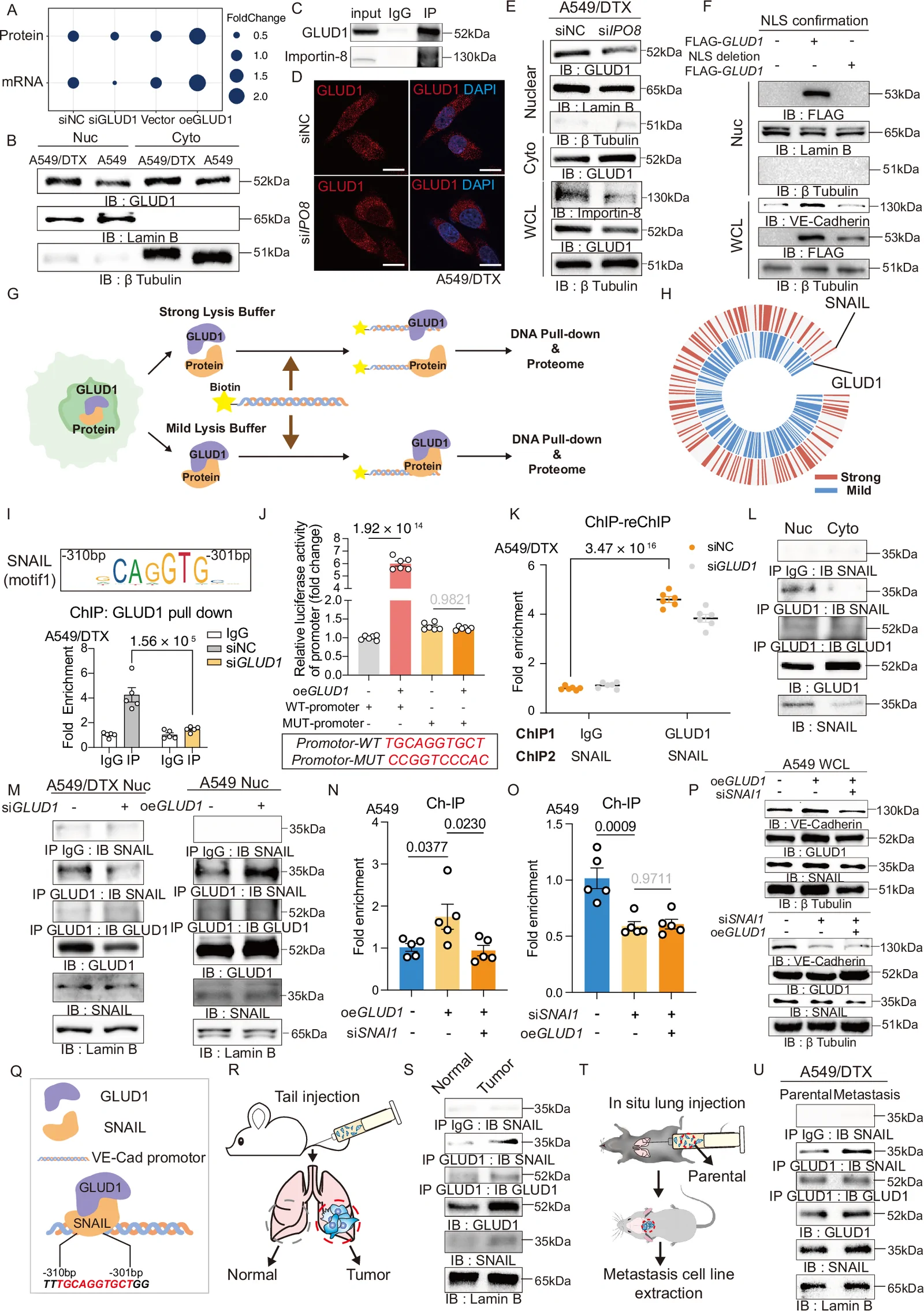
GLUD1 activates VE-Cadherin transcription by forming a nuclear complex with SNAIL. **A** VE-Cadherin protein and mRNA levels in NSCLC cells following GLUD1 knockdown or overexpression. **B** Representative immunoblotting analysis of GLUD1 distribution in nuclear and cytoplasmic fractions (*n* = 3 independent experiments). **C** Confirmed interaction of GLUD1 and Importin-8 with co-IP assay. A representative immunoblot from three independent experiments is shown. **D**, **E** GLUD1 localization assessed by immunofluorescence **D** and cytoplasm/nuclear levels with immunoblot assay **E** in A549/DTX following *IPO8* knockdown. Representative results from three independent experiments are shown. **F** Subcellular localization of FLAG-GLUD1 with or without its predicted nuclear localization sequence (NLS), together with VE-cadherin expression, in A549/DTX cells. The predicted NLS spans amino acids 517–550 and was identified using NLS Mapper (https://nls-mapper.iab.keio.ac.jp/). Representative results from three independent experiments are shown. **G** Workflow of DNA pulldown-coupled mass spectrometry with different preparation methods. **H** Circular heatmap of proteins identified under stringent (red) or mild (blue) conditions. **I** JASPAR-predicted SNAIL binding motif in the *CDH5* promoter and ChIP-qPCR analysis of GLUD1 occupancy at the predicted site in siNC- or si*GLUD1*-treated A549/DTX cells (*n* = 5 biologically independent samples). Data were normalized to input, Motif1: TGCAGGTGCT. **J** Dual-luciferase analysis of wild-type and mutant *CDH5* promoters in vector- or *GLUD1*-overexpressing A549 cells (*n* = 6 biologically independent samples), normalized to the wild-type promoter/vector group. **K** Sequential ChIP using anti-GLUD1 followed by anti-SNAIL antibodies, with IgG controls, in siNC- or si*GLUD1*-treated A549/DTX cells (*n* = 6 biologically independent samples). **L** Complex levels in cytoplasmic and nuclear fractions (representative of three independent experiments). **M** Nuclear complex in A549/DTX with si*GLUD1* treatment (left) or A549 with oe*GLUD1* treatment (right). Representative results from three independent experiments are shown. **N**, **O** ChIP analysis of GLUD1 and SNAIL binding on *CDH5* promoter following sequential si*SNAI1* and oe*GLUD1* treatments, normalized to input (*n* = 5 biologically independent samples). **P** Representative immunoblotting analysis of VE-Cadherin protein levels after these manipulations (*n* = 3 independent experiments). **Q** Proposed model of GLUD1/SNAIL complex binding to the *CDH5* promoter. **R**, **S** Processing of paired tumor-adjacent and tumor tissues (**R**) and analysis of nuclear complex levels (**S**, representative results from four biologically independent samples). **T**, **U** Sample-processing workflow **T** and analysis of nuclear complexes in leptomeningeal metastasis-competent A549/DTX cells (**U**, representative results from five biologically independent samples). Data are represented as mean ± SEM. *P* values were calculated using two-way ANOVA followed by Šídák multiple-comparisons test (**I**, **K**); one-way ANOVA followed by Tukey’s multiple-comparisons test (**J**); one-way ANOVA followed by Dunnett’s multiple-comparisons test (**N**, **O**). Scale bars: **D**, 20 μm. Source data are provided as a Source Data file. For immunoblots, samples within each panel were derived from the same experiment, with separate gels processed in parallel where applicable; detailed gel assignments are provided in the Methods.

GLUD1 regulates *CDH5* mRNA levels (Supplementary Fig. 4A), with a more pronounced effect than that observed at the protein level (Fig. 3A), pointing to its primary role in regulating VE-Cadherin transcription. Although several TCA cycle enzymes exhibit nuclear localization and exert non-canonical regulatory functions^39,40^, GLUD1 has long been regarded as a strictly cytoplasmic and mitochondrial enzyme primarily involved in basal metabolic processes^15,41^. Notably, recent work has shown that GLUD1 can act within the nucleus to regulate hepatitis B virus transcription^17^. This prompted us to investigate whether GLUD1 directly localizes to the nuclei of malignant tumor cells, how it gains nuclear access, and whether it participates in transcriptional regulation.

Through nuclear fractionation assays and DAPI co-localization microscopy, we verified that GLUD1 can localize to the nucleus (Fig. 3B), with its nuclear abundance dynamically regulated by genetic knockdown and overexpression (Supplementary Fig. 4B–D). Subcellular fractionation analysis further revealed that GLUD1 was predominantly localized in mitochondria (~60%), while approximately 23% and 17% were distributed in the nucleus and cytoplasm, respectively (Supplementary Fig. 4E, F). To elucidate the molecular mechanism underlying GLUD1 nuclear translocation, immunoprecipitation (IP)-Mass Spectrometry (MS) screening coupled by co-IP validation was adopted and revealed Importin-8 (encoding by *IPO8*) as the primary nuclear transporter for GLUD1 (Supplementary Fig. 4G and Fig. 3C), confirming by the reduction of nuclear GLUD1 accumulation upon *IPO8* knockdown; specifically, a 54% reduction in Importin-8 expression led to an approximately 32% decrease in the nuclear-to-cytoplasmic ratio (Fig. 3D, E and Supplementary Fig. 4H, I, showing results from two distinct siRNA sequences). Moreover, deletion of the GLUD1 nuclear localization signal (NLS) impaired its nuclear import, resulting in pronounced reduction of VE-Cadherin (Fig. 3F).

Having established GLUD1’s nuclear localization mechanism, we next investigated how nuclear GLUD1 exerts its transcriptional regulatory function, specifically whether nuclear GLUD1 directly binds the *CDH5* promoter as an autonomous transcriptional regulator or requires cooperation with other transcription factors^42^. To address this, we designed and performed a DNA pulldown-coupled MS assay (Fig. 3G). A synthesized biotinylated *CDH5* promoter fragment was incubated with A549/DTX cell lysates prepared under either stringent conditions that disrupt protein interactions or native conditions that preserve them. As shown in Fig. 3H, GLUD1 only associates with the biotinylated *CDH5* promoter under mild lysis conditions rather than under strong denaturing conditions, demonstrating its indirect DNA binding through protein interactions.

We next analyzed proteins enriched under both lysis conditions to identify those involved in GLUD1’s DNA binding. Among them, SNAIL, a core metastasis-related transcription factor encoding by *SNAI1*, emerged as the exclusive transcriptional regulator in our screening. The SNAIL binding motif within the *CDH5* promoter was predicted using JASPAR, and specific binding sites were validated by chromatin immunoprecipitation (ChIP) assays and luciferase reporter assays using a promoter variant with mutated binding sites (Fig. 3I, J and Supplementary Fig. 5A–C). In addition, JASPAR also predicted binding motifs for other relative transcription factors in the *CDH5* promoter (Supplementary Fig. 5D). However, these were ruled out due to either minimal enrichment after GLUD1 pulldown (e.g., SLUG/ZEB1 and SOX2 in Supplementary Fig. 5E) or unchanged binding site occupancy upon GLUD1 knockdown (e.g., TWIST in Supplementary Fig. 5F). Moreover, no detectable interaction was observed between GLUD1 and SLUG, ZEB1, SOX2, or TWIST (Supplementary Fig. 5G). Sequential ChIP (reChIP) experiments definitively confirmed GLUD1 and SNAIL co-occupy the same *CDH5* promoter region (Fig. 3K). Robust nuclear co-localization of GLUD1 and SNAIL in A549/DTX cells was confirmed by Immunofluorescence analysis (Supplementary Fig. 5H). Further Co-IP assay validated that there is an interaction between GLUD1 and SNAIL proteins, and this interaction occurs predominantly in the nucleus which can be modulated by GLUD1 expression levels (Fig. 3L, M and Supplementary Fig. 5I). Critically, *SNAI1* knockdown reversed the *GLUD1* overexpression-induced enrichment of *CDH5* promoter sequences, whereas *GLUD1* overexpression failed to rescue the reduced promoter binding and subsequent VE-Cadherin expression caused by *SNAI1* depletion (Fig. 3N–P). Consistently, *SNAI1* silencing abolished the enhancement of TEM induced by *GLUD1* overexpression (Supplementary Fig. 5J–O), whereas *SNAI1* overexpression failed to restore the impaired TEM phenotype resulting from *GLUD1* knockdown (Supplementary Fig. 5P–U). This unidirectional epistatic relationship also further validated that GLUD1 requires pre-existing SNAIL binding to associate with the *CDH5* promoter as shown in Fig. 3Q. Furthermore, *SNAI1* re-expression experiments following knockdown excluded potential off-target effects (Supplementary Fig. 5V, W), solidifying the mechanistic hierarchy in which GLUD1 depends on forming nucleus complex with SNAIL for promoter recruitment.

Meanwhile, comparative analysis showed that this interaction was markedly enriched in metastatic compared to contralateral normal lung tissues, where it was rarely detected (Fig. 3R, S). Notably, circulating tumor cells (CTCs) exhibited elevated nuclear GLUD1 and SNAIL levels together with increased VE-Cadherin expression, while leptomeningeal metastatic lesions derived from primary lung cancer displayed enhanced GLUD1-SNAIL interaction and VE-Cadherin expression compared to parental cells (Fig. 3T, U and Supplementary Fig. 5X, Y), further supporting the biological relevance of this complex in metastasis.

### The GLUD1 metabolite α-KG is essential for the GLUD1-SNAIL interaction

**Fig. 4:**
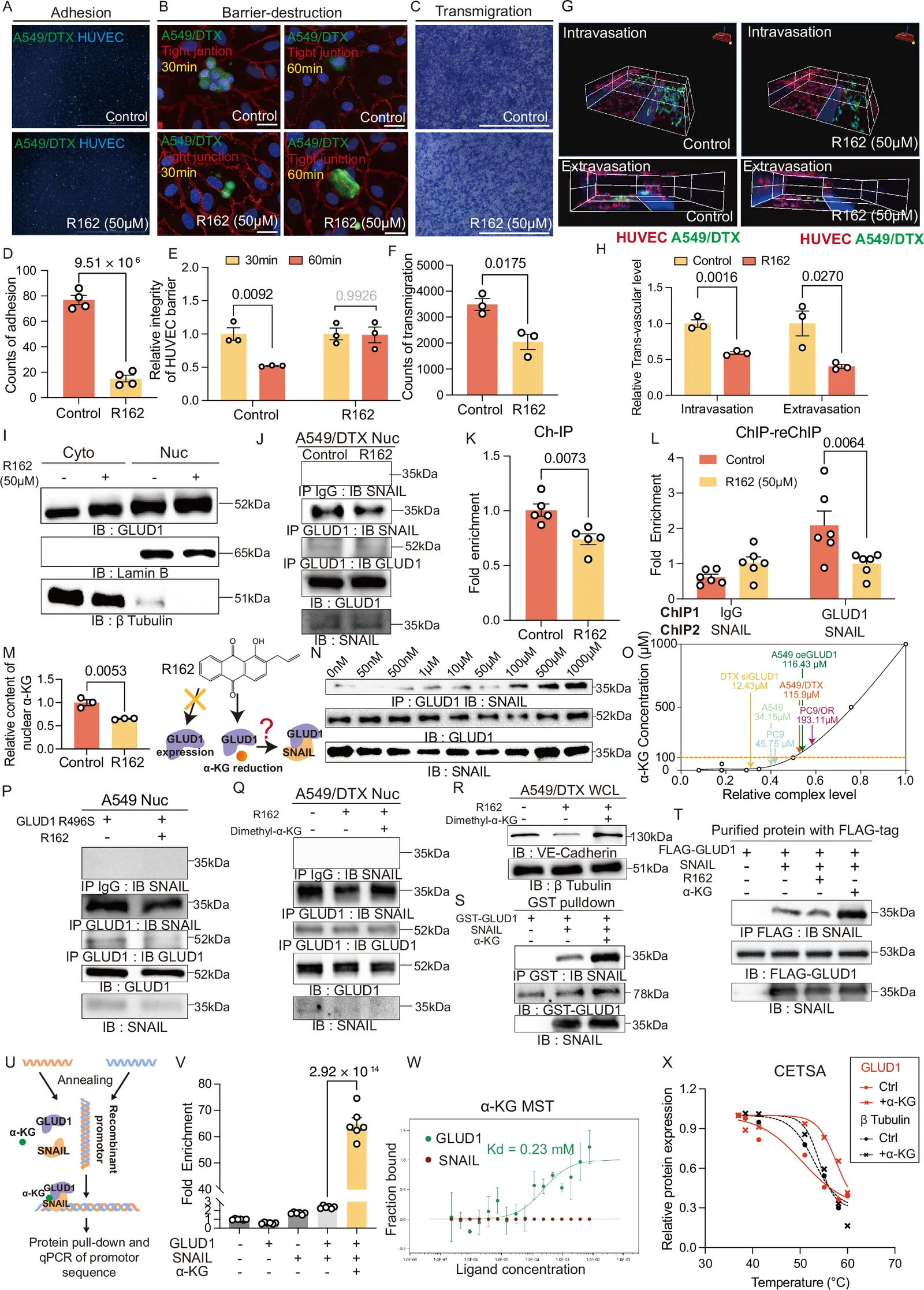
The GLUD1 metabolite α-KG is essential for the GLUD1-SNAIL interaction. **A**–**F** Representative images **A**–**C** and quantification (**D**–**F**) of adhesion (**A**, **D**, *n* = 4 biologically independent samples), barrier-destruction (**B**, **E**, *n* = 3 biologically independent samples) and transmigration (**C**, **F**, *n* = 3 biologically independent samples) in A549/DTX cells treated with DMSO or R162 (50 μM). Cells were counted; tight-junction fluorescence was normalized to 30 min. **G**, **H** Intravasation and extravasation under the same treatments (*n* = 3 biologically independent samples), normalized to control. **I**–**J** GLUD1 distribution **I** and nuclear GLUD1/SNAIL complexes **J** after R162 treatment (representative of three independent experiments). **K** GLUD1 binding to predicted SNAIL sites following 50 μM R162 treatment, all data were normalized to input, *n* = 5 biologically independent samples. **L** Sequential ChIP using anti-GLUD1 then anti-SNAIL, with IgG controls, under both treatments, *n* = 6 biologically independent samples. **M** Nuclear α-KG measured by LC-MS/MS under both treatments (*n* = 3 biologically independent samples), normalized to control. **N**, **O** Complex formation in purified proteins with 0–1000 μM α-KG (immunoblots, (**N**); quantification, (**O**)). Representative results from three independent experiments are shown. Arrows indicate the estimated nuclear α-KG concentrations in the indicated cell lines, each calculated as the mean of three biologically independent samples. **P** Nuclear complexes in R162-treated A549 cells expressing GLUD1 R496S (representative of three independent experiments). **Q**, **R** Nuclear complexes (**Q**) and VE-Cadherin expression (**R**) after rescue with 10 μM dimethyl-α-KG (representative of three independent experiments). **S**, **T** Pull-down assays of GLUD1/SNAIL binding using GST-tagged (**S**) or FLAG-tagged (**T**) GLUD1 with 100 μM α-KG (representative of three independent experiments). **U**, **V** Promoter pull-down analysis of α-KG/GLUD1/SNAIL ternary-complex formation with 100 μM α-KG (scheme (**U**); quantification (**V**); *n* = 6 biologically independent samples). **W** Microscale thermophoresis of α-KG binding to GFP-GLUD1 or GFP-SNAIL (0.00023–7.5 mM; *n* = 3 biologically independent samples). **X** CETSA validation of α-KG binding to GLUD1 using 10 μM dimethyl-α-KG. Quantification is from one representative experiment (*n* = 1); the experiment was independently repeated three times with similar results. The corresponding immunoblot is shown in Supplementary Fig. 6V. All data are represented as mean ± SEM. *P* values were calculated using two-tailed unpaired Student’s *t* test (**D**, **F**, **H**, **K**, **M**); two-way ANOVA followed by Šídák multiple-comparisons test (**E**, **L**); one-way ANOVA followed by Tukey’s multiple-comparisons test (**V**). Scale bars: **A**, **C** 1000 μm; **B** 20 μm. Source data are provided as a Source Data file. For immunoblots, samples within each panel were derived from the same experiment, with separate gels processed in parallel where applicable; detailed gel assignments are provided in the Methods.

R162, a selective GLUD1 enzyme activity inhibitor, potently suppressed VE-Cadherin transcriptional activation (Supplementary Fig. 6A) and subsequent protein expression both in vitro (Supplementary Fig. 6B–D) and in vivo (Supplementary Fig. 6E–G). This effect was recapitulated by EGCG, another GLUD1 enzymatic inhibitor (Supplementary Fig. 6E–G). R162 also inhibited tumor cell adhesion, barrier-destruction and transmigration across endothelial cells as validated in two tumor cell lines (Fig. 4A–F for A549/DTX and Supplementary Fig. 6H–M for PC9/OR). Additionally, R162 suppressed tumor cell intravasation and extravasation (Fig. 4G, H). Furthermore, without altering GLUD1 expression along with its capabilities of translocating into nucleus (Fig. 4I), R162 attenuated the GLUD1-SNAIL interaction (Fig. 4J) and their co-occupancy at the *CDH5* promoter (Fig. 4K, L). Thus, we raised a hypothesis that the reduction of nuclear α-KG resulted by R162 is involved in the regulation of complex assembly (Fig. 4M).

To test this hypothesis, we first examined the effect of α-KG on GLUD1-SNAIL interaction in purified protein system (Supplementary Fig. 6N). Increasing concentrations of α-KG progressively enhanced complex formation (Fig. 4N, O), with 100 μM identified as the threshold concentration for promoting complex assembly. Notably, this concentration is higher than the estimated local physiological concentrations of α-KG in the nucleus (6–50 μM)^43^. Using LC-TRAP/MS, we measured nuclear α-KG concentrations in the various cell lines used throughout this study. Notably, cell lines exhibiting stronger GLUD1-SNAIL interaction harbored higher nuclear α-KG levels, and these levels were above 100 μM in those lines (Fig. 4O).

To test whether this correlation reflects a causal role of GLUD1-generated α-KG, we generated cells expressing the catalytically dead mutant GLUD1 R496S^44^. In R496S-expressing cells, basal GLUD1 catalytic activity was already markedly impaired, and R162 failed to further reduce α-KG-dependent GLUD1-SNAIL complex formation (Fig. 4P and Supplementary Fig. 6O). We next supplemented α-KG in R162-treated A549/DTX cells. Here, we use methyl-α-KG due to its cell-permeable nature and ability to rapidly release α-KG upon intracellular hydrolysis^45^. α-KG restoration robustly increased nuclear GLUD1-SNAIL interaction (Fig. 4Q) and subsequent VE-Cadherin expression (Fig. 4R). Furthermore, the following GST pulldown and recombinant protein Co-IP assays in cell-free systems confirmed that α-KG is an enhancer in the assembly process of GLUD1-SNAIL complex (Fig. 4S, T).

To determine whether α-KG modulates the binding of the protein complex to the promoter, we synthesized recombinant promoter DNA sequences and incubated them with different combinations of α-KG, recombinant GLUD1, and recombinant SNAIL. After pull-down assays, we quantified the binding affinity of GLUD1/SNAIL for promoter DNA via qPCR (Fig. 4U). As shown in Fig. 4V, neither GLUD1 nor SNAIL alone recruited the *CDH5* promoter, whereas α-KG significantly enhanced binding of the GLUD1-SNAIL complex to this promoter. Meanwhile, using the inhibitor of another α-KG upstream cytoplasmic enzyme IDH1, also significantly impaired the nuclear α-KG abundance and complex level in nucleus (Supplementary Fig. 6P, Q), emphasizing the necessity of α-KG for complex assembly. Notably, other endogenous metabolites structurally similar to α-KG, including Glutamate, Succinate, Fumarate, Malate and D/L-2-HG, failed to significantly promote either complex formation or its binding to the promoter, underscoring the specificity of α-KG (Supplementary Fig. 6R–U).

Next, a key question arose: does α-KG influence complex formation by binding to the GLUD1-SNAIL complex? Microscale thermophoresis (MST) demonstrated that α-KG preferentially binds to GLUD1 rather than SNAIL (Fig. 4W). This finding was further supported by cellular thermal shift assays (CETSA, Fig. 4X; Supplementary Fig. 6V) and drug affinity responsive target stability assays (DARTS, Supplementary Fig. 6W). Together, these results suggest that α-KG likely binds to GLUD1, forming an α-KG/GLUD1/SNAIL ternary complex.

### α-KG acts as a conformational stabilizer within the α-KG/GLUD1/SNAIL ternary complex

**Fig. 5:**
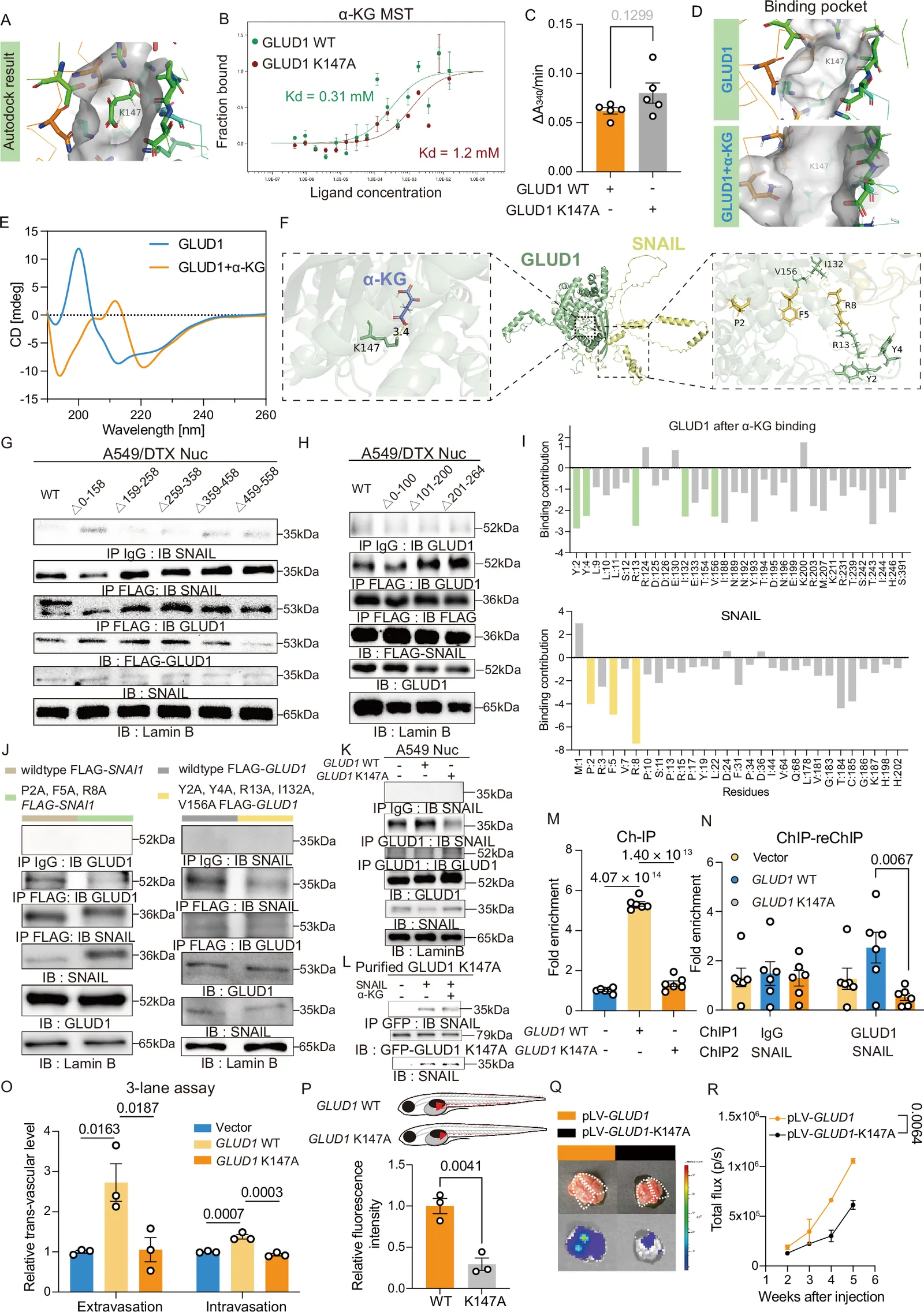
Structural assembly model of the α-KG/GLUD1/SNAIL ternary complex. **A** Predicted α-KG binding pocket in GLUD1 (AutoDock) and visualization using PyMOL. **B** MST confirming disrupted α-KG binding to K147A-GLUD1 mutant comparing with wildtype GLUD1, the concentration of α-KG ranges from 15 mM to 0.00046 mM, *n* = 3 biologically independent samples. **C** Metabolic activity of wild-type versus K147A-GLUD1, *n* = 5 biologically independent samples. **D** Conformational changes in GLUD1 upon α-KG binding. **E** Circular Dichroism result of GLUD1 with or without 100 μM α-KG. A representative result from three independent experiments is shown. **F** Molecular dynamics simulations revealing α-KG/GLUD1/SNAIL interaction interfaces visualizing with PyMOL, The displayed structural snapshot was obtained from one MD simulation trajectory. **G**, **H** Domain mapping: GLUD1 (amino acids 1–158) and SNAIL (amino 1–100) mediate complex formation (representative of three independent experiments). **I** Residue interaction maps for GLUD1-SNAIL complex formation with molecular dynamics assay, binding sites with significantly high contribution in GLUD1 (amino acids 1–158) and SNAIL (amino 1–100) were chosen. **J** Residue mapping: GLUD1 (amino acids Y2, Y4, R13, I132, V156) and SNAIL (amino acids P2, F5, R8) mediate complex formation (representative of three independent experiments). **K** Impaired binding between K147A-GLUD1 and SNAIL in cell nucleus (representative of three independent experiments). **L** Impaired binding between K147A-GLUD1 and SNAIL in purified protein system despite 100 μM α-KG treatment (representative of three independent experiments). **M**, **N** ChIP (**M**) and sequential ChIP (**N**) showing defective promoter recruitment with K147A-GLUD1, all data were normalized to input, *n* = 6 biologically independent samples. **O** Quantified intra/extravasation efficiency of wild-type versus K147A-GLUD1 in 3-lane vascular model, *n* = 3 biologically independent samples. **P** Relative quantification of migrating cells’ fluorescence intensity in GLUD1 variants using intravasation based zebrafish model, *n* = 3 embryo cohorts. **Q**, **R** In vivo metastasis assay using extravasation based mouse model: (**Q**) representative bioluminescence images; (**R**) weekly photon flux quantification (GFP-luciferase-tagged A549/DTX cells injected via tail vein in Balb/c nude mice), *n* = 4 mice. All data are represented as mean ± SEM. *P* values were calculated using two-tailed unpaired Student’s *t* test (**C**, **P**); one way ANOVA followed by Dunnett’s multiple-comparisons test (**M**, **O**); two way ANOVA followed by Šídák multiple-comparisons test (**N**); two way ANOVA (**R**). Source data are provided as a Source Data file. For immunoblots, samples within each panel were derived from the same experiment, with separate gels processed in parallel where applicable; detailed gel assignments are provided in the Methods.

Using Autodock predictions (Fig. 5A) followed by MST validation with point mutant recombinant proteins (Fig. 5B), we identified K147 (Lys), a highly conserved residue across species (Supplementary Fig. 7A), as the primary α-KG binding site in GLUD1. Enzymatic characterization of the K147 mutant confirmed this to be a non-catalytic binding site (Fig. 5C). As predicted by molecular dynamics simulations, α-KG binding induces conformational changes in GLUD1 (Fig. 5D). Circular Dichroism (CD) spectroscopy directly demonstrated this structural effect, with a distinct spectral shift confirming α-KG-driven alterations in secondary structure (Fig. 5E). This structural change induced by α-KG binding enhances the interaction between GLUD1 and SNAIL (Fig. 5F). Through fragment deletion analysis of GLUD1 and SNAIL, residues 1–158 of GLUD1 and 1–100 of SNAIL were identified as protein-protein binding domains (Fig. 5G, H). Molecular dynamics simulations of the α-KG bound GLUD1/SNAIL complex revealed a set of predominantly conserved, high-contribution interfacial residues: TYR2, TYR4, ARG13, ILE132, and VAL156 in GLUD1, and PRO2, PHE5, and ARG8 in SNAIL, predicted to mediate stable complex formation (Fig. 5I and Supplementary Fig. 7B, C). Mutating these residues in either GLUD1 or SNAIL significantly impaired their protein-protein interaction, in agreement with the molecular dynamics predictions. (Fig. 5J). Moreover, the K147A mutation, which decreased α-KG binding as shown in Fig. 5B, significantly weakened the GLUD1-SNAIL interaction (Fig. 5K). Even with exogenous α-KG supplementation, the K147A mutation deprived the ability of α-KG to promote GLUD1-SNAIL binding (Fig. 5L). Furthermore, mutation of this α-KG binding site in GLUD1 substantially reduced the occupancy of the GLUD1/SNAIL transcriptional complex at the *CDH5* promoter (Fig. 5M, N). This was accompanied by impaired TEM ability, affecting both the intravasation process (evidenced by Fig. 5O for 3-lane model and Fig. 5P for zebrafish xenografts) and the extravasation process (Fig. 5O for 3-lane model and Fig. 5Q, R for tail vein injection model).

### Competitive inhibition of α-KG engagement blocks ternary complex formation and suppresses TEM-driven metastasis

**Fig. 6:**
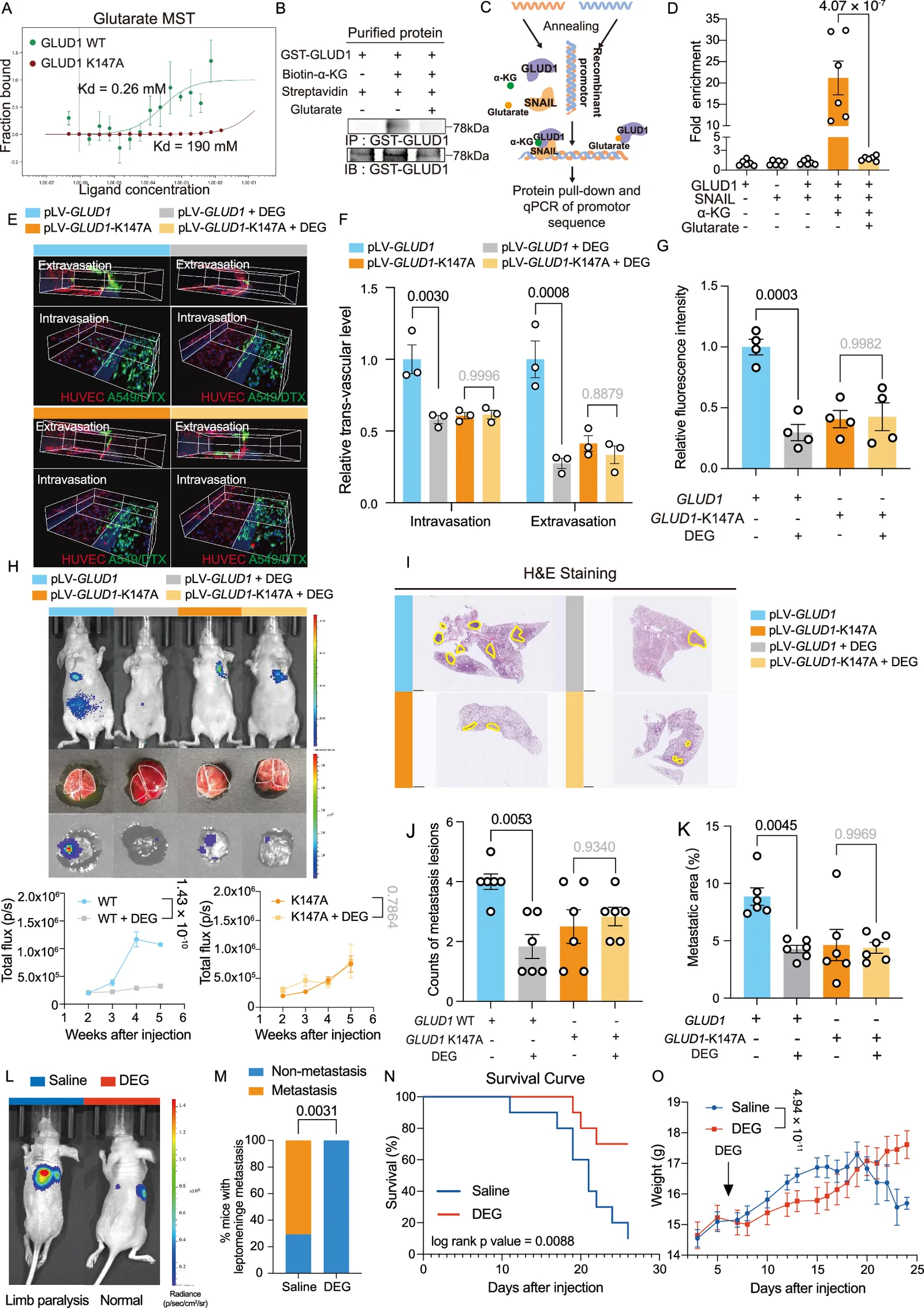
Competitive inhibition of α-KG engagement blocks ternary complex formation and suppresses TEM. **A** MST analysis of diethyl glutarate (DEG) binding to GFP-tagged wild-type (WT) or K147A mutant GLUD1 in A549 cell lysates, the concentration of DEG ranges from 15 mM to 0.00046 mM, *n* = 3 biologically independent samples. **B** Streptavidin pull-down assay showing competition between biotinylated α-KG and glutarate (100 μM each) for GST-GLUD1 binding (representative of three independent experiments). **C**, **D** CDH5 promoter pull-down analysis of GLUD1-SNAIL binding with α-KG or glutarate (100 μM each) (scheme, (**C**); quantification, (**D**); *n* = 6 biologically independent samples). **E**, **F** 3-lane vascular model images (**E**) and quantification (**F**) of TEM in A549/DTX cells expressing WT or mutant GLUD1 with DEG (10 μM) treatment, *n *= 3 biologically independent samples, normalized to pLV-*GLUD1*. **G** Intravasation based zebrafish metastasis model showing fluorescence intensity of migrating A549/DTX cells microinjected with 1  nL 7 M DEG, *n* = 4 embryo cohorts. **H** Bioluminescence tracking of metastatic burden in nude mice intravenously injected with GFP-luciferase-tagged A549/DTX cells and treated with DEG (10 mg/kg, i.p., every other day), *n* = 6 mice. **I**–**K** H&E staining (**I**), metastatic lesion number (**J**) and metastatic area fraction (**K**) in lungs from (**H**) (*n* = 6 mice). Metastatic area fraction was calculated as metastatic area divided by total lung area. **L** Bioluminescence imaging of nude mice implanted in situ with metastatic A549/DTX cells and treated with saline or DEG. Saline-treated mice developed brain metastases with hindlimb paralysis, whereas DEG-treated mice showed normal behavior. **M** Proportion of mice developing brain metastases (*n* = 10 mice). **N** Survival and **O** body weight of the mice in (**L**) (*n *= 10 mice). Data are represented as mean ± SEM. *P* values were calculated using one way ANOVA followed by Tukey’s multiple-comparisons test (**D**, **F**, **G**, **J**, **K**); two way ANOVA (**H**, **O**); two-sided Fisher’s exact test (**M**); two-sided log-rank (Mantel-Cox) test (**N**). Scale bars: **I**, 500 μm. Source data are provided as a Source Data file. For immunoblots, samples within each panel were derived from the same experiment, with separate gels processed in parallel where applicable; detailed gel assignments are provided in the Methods.

Glutarate structurally resembles α-KG and has been proposed as a competitive inhibitor of α-KG-dependent metabolic enzymes^46^. MST assays confirmed that glutarate directly binds to GLUD1, and mutation of K147 abolished this interaction, indicating that glutarate, like α-KG, interacts with the K147 residue of GLUD1 (Fig. 6A). However, unlike α-KG, glutarate did not alter the secondary structure of GLUD1 (Supplementary Figs. 8A vs.  5E), suggesting that it acts as a competitive inhibitor that blocks α-KG engagement and consequently prevents ternary complex formation. For further validation, we synthesized biotinylated α-KG and incubated it with GST-tagged GLUD1, followed by streptavidin bead pull-down. As shown in Fig. 6B, glutarate significantly reduced the interaction between GLUD1 and α-KG. Consistent with this finding, both in vitro recombinant protein system and cellular system demonstrated that glutarate effectively suppresses α-KG-induced formation of the GLUD1-SNAIL complex (Supplementary Fig. 8B, C). For cellular experiments, diethyl glutarate (DEG) was used due to its cell-permeable nature and ability to rapidly release glutarate upon intracellular hydrolysis. Further in vitro DNA promoter-contained pull-down assays revealed that glutarate competitively inhibits transcriptional complex binding at the *CDH5* promoter (Fig. 6C, D), concomitant with reduced VE-Cadherin induction (Supplementary Fig. 8D). Finally, we validated the functional consequences of glutarate treatment and found it impaired TEM ability, affecting both the intravasation and extravasation processes. For intravasation, glutarate significantly reduced tumor cell migration from the stromal side into the vascular lumen in a 3-lane model (Fig. 6E, F) and attenuated metastatic dissemination in a zebrafish intravasation model (Fig. 6G). For extravasation, glutarate significantly reduced tumor cell egress from the vascular lumen into the stroma in the same 3-lane model (Fig. 6E, F) and resulted in fewer metastatic lesions in a murine extravasation model (Fig. 6H–K). Crucially, glutarate failed to exert these effects in tumor cells carrying the GLUD1 with K147A mutation, confirming that its activity depends on competing with α-KG for binding at the K147 site, thereby inhibiting α-KG/GLUD1/SNAIL complex formation.

In addition to the step-specific models described above, we also employed an in-situ lung tumor metastatic model to validate our intervention strategy. This model is commonly used to assess the complete metastatic cascade, as it combines both intravasation and extravasation within a single system. As shown in Fig. 6L–O, DEG administration reduced brain metastatic burden (Fig. 6L) and decreased the proportion of mice developing metastasis-associated hindlimb paralysis (0/10 versus 7/10; two-sided Fisher’s exact test, *P* = 0.0031; absolute risk reduction = 0.70, 95% CI, 0.29–0.89; Fig. 6M). DEG treatment also prolonged survival (Fig. 6N) and attenuated body weight loss (Fig. 6O).

Additionally, we further explored the clinical relevance of the GLUD1/VE-Cadherin axis across multiple cancer types using TCGA datasets. Notably, GLUD1 expression positively correlated with *CDH5* gene levels in high-VM breast cancer and melanoma cohorts (Supplementary Fig. 8E, F). Moreover, *CDH5* expression was significantly elevated in advanced-stage tumors compared with early-stage tumors, supporting a potential role for this pathway in other malignancies, as we showed in NSCLC models (Supplementary Fig. 8G, H).

## Discussion

**Fig. 7:**
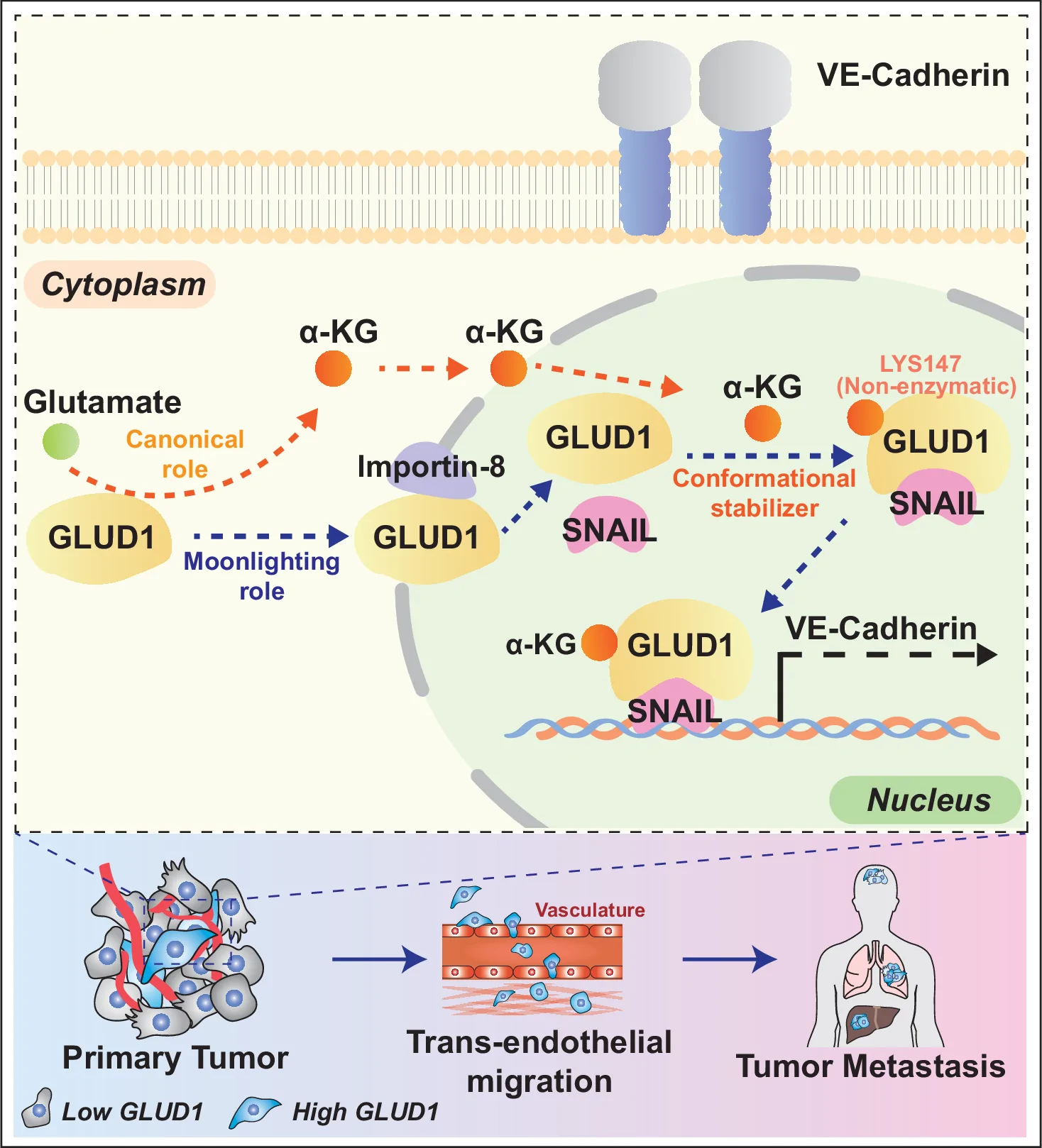
Schematic model of α-KG-dependent GLUD1-SNAIL complex formation during trans-endothelial migration and metastasis. In the cytoplasm, GLUD1 performs its canonical metabolic function in glutamate catabolism by generating α-KG. In addition to this enzymatic role, a fraction of GLUD1 undergoes Importin-8-dependent nuclear translocation and exerts a moonlighting function. Within the nucleus, GLUD1 associates with the transcription factor SNAIL. Nuclear α-KG binds to the non-catalytic Lys147 site of GLUD1 and acts as a conformational stabilizer, thereby promoting and stabilizing the interaction between GLUD1 and SNAIL. This metabolite-dependent interaction supports the assembly of a nuclear α-KG/GLUD1/SNAIL ternary complex. The resulting complex is recruited to the VE-cadherin promoter and enhances VE-cadherin transcription in tumour cells. Increased VE-cadherin expression strengthens trans-endothelial migration and facilitates tumor cell intravasation into and extravasation from the vasculature, two critical steps in hematogenous metastatic dissemination. The lower panel summarizes the biological consequence of this pathway, in which tumour cells with relatively high GLUD1 expression more readily interact with the vasculature, undergo trans-endothelial migration and contribute to the formation of distant metastases, whereas tumour cells with relatively low GLUD1 expression show a reduced capacity for these processes. Orange circles represent α-KG, yellow ovals represent GLUD1, pink ovals represent SNAIL and the purple structure represents Importin-8. The blue and grey tumour-cell symbols indicate relatively high and low GLUD1 expression, respectively. Dashed arrows indicate the proposed directional steps involved in α-KG production, GLUD1 nuclear translocation, ternary-complex assembly and transcriptional regulation. The schematic summarizes the mechanism supported by the experimental findings and is not drawn to scale.

TEM is a dynamic and essential step in the metastatic cascade, enabling tumor cells to intravasate and extravasate the vasculature and thereby drive tumor dissemination. Yet whether metabolic enzymes, long regarded solely as catalysts of cellular metabolism, can directly moonlight as regulators of the transcriptional programs governing this process remains largely unexplored. Our findings establish α-KG as a conformational stabilizer that enables the formation of a nuclear α-KG/GLUD1/SNAIL complex potentiating the ability of tumors to intravasate and extravasate through vasculature. Furthermore, through detailed mechanistic dissection of the assembly mode of this ternary complex, we uncover previously unreported moonlighting functions of both α-KG and GLUD1 (Fig.7).

α-KG exhibits pleiotropic functions in tumor progression that extend beyond core metabolism. As an essential cofactor for dioxygenases, α-KG regulates epigenetics and epitranscriptomics^47,48^ and it can also acts as a signaling molecule by directly binding and modulating protein activity^49,50^. Although previous study has detected GLUD1 and α-KG in the nucleus^17,43^, where it modulates histone methylation via α-KG-dependent dioxygenases to influence epigenetic remodeling^51^, the nuclear entry of GLUD1 remains mechanistically unexplained. Our study delineates a coherent mechanistic framework that GLUD1 can translocate into the nucleus via Importin-8 and directly interacts with SNAIL, with α-KG functioning as an essential conformational stabilizer. Structural and mutagenesis analyses revealed that α-KG docks at the hydrophilic K147 site of GLUD1, a non-enzymatic binding pocket that influences GLUD1 conformation, enabling its enhanced interaction with SNAIL. This interaction forms a binding domain comprising Y2, Y4, R13, I132 and V156 in GLUD1, along with P2, F5, and R8 in SNAIL. The resulting α-KG/GLUD1/SNAIL complex binds to the *CDH5* promoter, transcriptionally upregulating its expression, thereby promoting tumor cell TEM and metastasis. In addition to SNAIL^52^, another transcription factor TWIST1 also has been reported to directly bind with the *CDH5* promoter and upregulate tumor cells’ transactivation and VM ability in hepatocellular carcinoma^53^ while our experiments conclusively demonstrated the absence of GLUD1-TWIST1 interaction. Notably, VM has been widely used as a morphological feature associated with aggressive tumor behavior. We therefore employed it as a phenotypic screening marker to identify subpopulations with enhanced TEM capacity, which subsequently led us to identify GLUD1 as a key regulator of this process. It should be emphasized that the GLUD1-VM correlation is associative, and we do not claim a mechanistic connection between VM structures and metastasis. Rather, our study focuses on the role of GLUD1 in regulating TEM-related tumor cell behavior, whereas the mechanistic basis of VM formation and its causal contribution to metastasis remain beyond the scope of the current work.

Compared to normal cells, tumor cells co-opt metabolic enzymes not only to fulfil cellular metabolic demands but also to regulate diverse non-metabolic processes^54,55^. These non-canonical roles of endogenous metabolic enzymes have garnered increasing attention due to their pivotal regulatory functions in tumorigenesis^16^, particularly glucose-metabolizing enzymes such as HK^56,57^, PFK^58^, PGK1^59^, PKM2^60,61^ and PCK1^62^, which act as protein kinases with phosphorylating a broad spectrum of substrates to drive tumor progression. Beyond modulating post-translational modifications, glucose metabolic enzymes further diversify their functions via protein-protein interactions, altering the activity of binding partners in a catalysis-independent manner^63–65^. While glutamine catabolism also plays a crucial role in tumorigenesis and progression^12^, evidence for moonlighting functions of glutamine-related metabolic enzymes remains limited. In the cytosol, phosphorylated GLUD1 (Ser384) interacts with RelA and IKKβ to form an oncogenic complex^66^. Our current work reveals analogous functionality in the nucleus. GLUD1 engages in protein-protein interactions with SNAIL, while its metabolic product α-KG similarly regulates complex assembly. These findings significantly expand the known scope of GLUD1’s moonlighting functions and suggest that similar to glucose-metabolizing enzymes, glutamine-related metabolic enzymes may possess diverse regulatory capabilities warranting broader investigation. Although the cell lines and animal models used in this study are primarily based on lung cancer models and leptomeningeal metastasis of lung cancer, the initial screening based on clinical samples in Fig. 1 incorporated multiple tumor types. In addition, our TCGA-based analyses suggest that the GLUD1/VE-cadherin axis may have broader relevance beyond NSCLC, although the strength of this association varied across cancer types. The variable correlation strengths between GLUD1 expression and VE-Cadherin across lung cancer, breast cancer, and melanoma may be partly attributable to the nature of bulk TCGA transcriptomic data, which may be influenced by differences in tumor purity, vascular density, stromal composition, endothelial cell abundance, and immune infiltration across cancer types. In addition, cancer type-specific molecular backgrounds, metabolic dependencies and baseline propensities may further contribute to the variable correlation patterns observed among different malignancies. Therefore, the applicability and clinical value of the α-KG/GLUD1/SNAIL axis in other cancer types require further systematic investigation, including assessment of complex abundance across a broader spectrum of malignancies and evaluation of strategies targeting α-KG/GLUD1-mediated TEM and metastasis.

Many disease-associated metabolism enzymes have been targeted through the development of small-molecule compounds for therapeutic intervention, particularly in cancer^67^. However, conventional approaches to intervention in the canonical catalytic activities of these enzymes unavoidably disrupt systematic metabolism, which is essential for maintaining the homeostasis of normal cells, such as immune cells, and organs, such as the brain. This disruption often leads to severe side effects. The discovery of the moonlighting functions of metabolic enzymes and specific mechanism, underscores alternative approaches to mitigating these non-canonical functions for treating cancer. In this study, we reveal that GLUD1 regulates TEM through a dual function that integrates its canonical metabolic activity with moonlighting functions through both protein-protein interactions and metabolic regulation, the latter mediated by α-KG-dependent modulation of complex assembly. By competitively inhibiting the conformational binding of α-KG with GLUD1, we were able to block the assembly of the ternary complex and suppress the TEM process. α-KG has attracted considerable attention for its well-established roles in anti-aging and maintenance of cellular stemness and it has gained popularity as a daily anti-aging supplement^68–73^. However, for cancer patients, α-KG supplementation may not yield positive outcomes. Although α-KG has traditionally been considered tumor-suppressive^47,48^, emerging evidence reveals a contrasting oncogenic role: α-KG accumulation can promote immune evasion^50^ and confer resistance to disulfidptosis^74^. Similarly, our study identifies α-KG as a factor supporting tumor metastasis. Unlike previous reports describing α-KG as a cofactor for dioxygenases that modulate epigenetics to promote tumorigenesis, we reveal that α-KG acts as a conformational stabilizer facilitating transcriptional complex assembly, a previously unrecognized mechanism. The addition of α-KG alters the secondary structure while Glutarate act as non-functional ligand. Therefore, Glutarate administration exerts competitive inhibition of this interaction effectively reduces TEM in tumor cells. However, given α-KG’s pivotal physiological functions, systemic inhibition of α-KG using non-targeted metabolic analogs inevitably induces toxic side effects. Meanwhile, glutarate is an endogenous metabolite whose physiological ratio with crotonyl-CoA is tightly regulated. Excessive glutarate accumulation exhibits cytotoxicity, as evidenced in glutaric acidemia type I patients harboring GCDH loss-of-function mutations, who develop severe neurodevelopmental impairments^46,75,76^. Although glutarate demonstrates anti-metastatic properties, supratherapeutic accumulation due to impaired clearance mechanisms triggers metabolic dysregulation, including feeding abnormalities in murine models^77^. While we have characterized the complex using AlphaFold predicted protein model and MD simulations, these findings underscore the necessity of developing novel compounds, contingent upon precise structural characterization of the α-KG/GLUD1/SNAIL ternary complex to overcome the inherent uncertainties, for instance, using interface-based peptidomimetics^78^.

In summary, our study identifies the conformational regulatory role of α-KG and the assembly of the nuclear α-KG/GLUD1/SNAIL complex as a key mechanism mediating the often-overlooked process of TEM during tumor dissemination. Notably, preferential assembly of this complex in tumor tissues provides a mechanistic basis for selective therapeutic intervention. Targeting the non-canonical α-KG-GLUD1 interface, rather than enzymatic activity, offers a strategy to disrupt metastatic progression while potentially sparing normal metabolic functions. Our findings provide a potential therapeutic innovation, underscoring the need for deeper structural insight and structure-guided therapeutic designs into this α-KG-driven nuclear complex. However, further studies, including detailed structural characterization and in vivo evaluation of safety and pharmacological properties, will be required to assess the feasibility of therapeutic development based on this mechanism.

## Methods

### Ethics statement

All experiments complied with the relevant ethical regulations. The use of de-identified archival human NSCLC specimens was approved by the Research Ethics Committee of the First Affiliated Hospital of Nanjing Medical University (approval no. 2019-SR-450). Written informed consent to participate in the research was obtained from all participants. All animal procedures were approved by the Institutional Animal Care and Use Committee of China Pharmaceutical University (approval no. SYXK2021-0010) and were conducted in accordance with the Guide for the Care and Use of Laboratory Animals.

### Reagents and tools

All reagents and resources used in this study, including antibodies, chemicals, recombinant proteins, critical commercial assays and software, are listed in Supplementary Data 1.

### Cell culture

All tumor cell lines were obtained from the Cell Bank of the Chinese Academy of Sciences (Shanghai, China). Human lung cancer cells (PC9, H460, A549, PC9/OR, H1975, A549/DTX) were maintained in RPMI 1640 supplemented with 10% fetal bovine serum (FBS), 100 U/mL penicillin, and 100 μg/mL streptomycin. Primary Human Umbilical Vein Endothelial Cell (HUVEC) was purchased from Shanghai Zhong Qiao Xin Zhou Biotechnology Co., Ltd and cultured in endothelial cell culture medium (ZQ-1304, Shanghai Zhong Qiao Xin Zhou Biotechnology Co., Ltd).

### Plasmid transfections, viral infections and RNA interference

For transient plasmid transfection, parental cells were seeded in 6-well plates at 5 × 10^5^ cells per well and transfected with 1 μg plasmid using lipofectamine 3000 (Invitrogen, L3000015) in serum-free RPMI 1640. After 6 h incubation, the medium was replaced with complete RPMI 1640 containing 10% FBS. Transfected cells were harvested 2-4 days post-transfection for subsequent assays. *GLUD1* overexpression vectors (wild-type, GFP-tagged, and K147A mutant), FLAG-tagged GLUD1 deletion mutants (wild-type, Δ1-158, Δ159-258, Δ259-358, Δ359-458, Δ459-558), FLAG-tagged *SNAI1* deletion mutants (Δ1-100, Δ101-200, Δ201-264), FLAG-tagged GLUD1 point mutants (Y2A/Y4A/R13A/I132A/V156A), FLAG-tagged *SNAI1* point mutants (P2A, F5A, R8A) and the *CDH5* promoter-driven dual-luciferase reporter system (wild-type and mutant) was constructed as described. Plasmids pCDNA3.1 containing FLAG-tagged constructs were obtained from Genomeditech (Shanghai, China), while pEGFP-*GLUD1* variants and luciferase reporters were purchased from Jinbeijin Biotechnology (Nanjing, China).

For stable lentiviral transduction, lentiviral vectors (pLV-CMV-MCS-GFP-luciferase-puro and its *GLUD1* variants) were obtained from Jinbeijin Biotechnology. Cells were infected at multiplicities of infection of 50 (A549/DTX, H1975) or 20 (PC9, A549) in serum-free medium, followed by selection with 4 μg/ml puromycin.

The sequences of all relevant constructs are provided in Supplementary Data 2.

### Animal treatment

Zebrafish (*Danio rerio*) were supplied by Hunter Biotech. For the zebrafish xenograft model, tumor cells (1 × 10^6^) were washed twice with PBS, stained with 1 μg CellTracker^TM^ Cm-DiI (Thermofisher, USA) in 1 mL PBS at 37 °C for 5 min, then centrifuged (1000 × *g*, 3 min) and harvested. After three PBS washes, cells were injected into the yolk sac or intervitelline space of 48 hpf zebrafish larvae using a capillary needle. At 24 hpi, zebrafish with successful engraftment were selected under a stereomicroscope and maintained at 34 °C. Metastasis was assessed at 96 hpi by fluorescence imaging (stereomicroscope) and quantified using FIJI software (ImageJ). For the zebrafish xenograft experiments, tumor cells were injected into 48 hpf zebrafish larvae, and metastasis was assessed at 96 hpi. During this experimental window, biological sex cannot be reliably determined; therefore, sex was not considered as a biological variable in these experiments.

Female BALB/c nude mice (3–4 weeks old) were purchased from Beijing Vital River Laboratory Animal Technology Co., Ltd. and housed in individually ventilated cages in the specific pathogen-free facility of the Laboratory Animal Center, China Pharmaceutical University. Mice were maintained at 22 ± 2 °C and 50 ± 10% relative humidity under a 12-h light/12-h dark cycle. They were provided with an irradiated maintenance diet (XT101WC-009; Jiangsu Synergy Pharmaceutical Bioengineering Co., Ltd., China) and water ad libitum. Cages contained irradiated corncob bedding (8 mesh; catalogue no. 1060012; Jiangsu Synergy Pharmaceutical Bioengineering Co., Ltd., China).

For mouse metastasis model in Figs. 1 and  5, Luciferase-expressing tumor cells (1 × 10⁶ in 200 μL PBS) were intravenously injected into 3–4 week old female BALB/c nude mice. Metastasis was monitored by bioluminescence imaging (IVIS Spectrum, Revvity) at days 2, 7, 14, and 28 post-injection following luciferin administration (150 mg/kg). Lungs were harvested for either fixation (4% PFA) or Western blot analysis. H&E and PAS + /CD31− staining assays were performed by Servicebio (China), with whole-slide scanning (NanoZoomer S210, Hamamatsu) and quantification (NDP.view2).

For drug-treated mouse metastasis model in Fig. 6H–K, A549/DTX cells expressing luciferase with either wild-type or K147A GLUD1 (1 × 10⁶) were injected as above. Treatment groups received DEG (10 mg/kg, i.p.) every other day. Imaging and analysis followed the same protocol.

For mouse metastasis model in Fig. 6L–O, A549/DTX cells expressing GFP-luciferase (5 × 10⁶) were orthotopically injected into mouse lungs. Mice were treated with Saline/DEG (10 mg/kg, i.p., every 3 days) and hindlimb paralysis was recorded as a metastasis-associated neurological outcome.

For the zebrafish xenograft experiments, the endpoint of 96 hpi was prospectively defined to allow assessment of metastatic dissemination within the established experimental window. For all tail-vein metastasis models, including those shown in Figs. 1, 5 and 6H–K, the endpoint of 5 weeks after tumour-cell injection was prospectively defined based on our previous experience with these models and the expected time required for the development of detectable metastatic lesions. For the orthotopic lung tumour model shown in Fig. 6L–O, the experiment was designed to evaluate survival. Animals were followed for survival, and the experiment was terminated on day 26, when cumulative mortality in the saline-treated group reached 90%. All surviving animals were euthanized at this time, and tissues were collected for subsequent analyses. Because the tail-vein and orthotopic lung tumour models involved internally located tumours, caliper-based measurements were not applicable. Tumour burden was monitored by bioluminescence imaging and clinical assessment. No animal exceeded the maximum tumour burden permitted under the approved animal protocol before the respective experimental endpoints.

### Tube formation assays

Pre-chill a 96-well plate on ice and coat each well with 50 μL of Matrigel (Corning, #354230). Polymerize the Matrigel by incubating the plate at 37 °C for 2 h. Harvest and count tumor cells from experimental groups, then seed 2 × 10⁴ cells per well in 200 μL of RPMI 1640 supplemented with 2% fetal bovine serum (FBS) onto the Matrigel-coated wells. Incubate the plate at 37 °C with 5% CO₂ for 4–6 h. Tube formation images were taken using an Agilent BioTek Lionheart™ automated microscope. Quantify tube formation using Fiji (ImageJ) with the Angiogenesis Analyzer2 plugin to automatically assess endothelial network parameters (e.g., tube length, branching points).

### RNA analysis by quantitative PCR

Total RNA was isolated using TRIzol Reagent, followed by cDNA synthesis with the HiScript Reverse Transcriptase kit (Vazyme). Quantitative PCR was performed on a CFX96 Real-Time System (Bio-Rad) using SYBR Green PCR Master Mix (Bio-Rad) under the following conditions: 95 °C for 15 sec (denaturation), 60 °C for 30 sec (annealing/extension) for 40 cycles. Melting curve analysis verified primer specificity. Relative gene expression was calculated by the 2−ΔΔCt method using ACTIN as the endogenous control. The primers used are shown as below: *CDH5*-F CACTTCCCCATCATGTAGGCAA, *CDH5*-R TTTTCGCCAGTGTCCTTG TCC, *ACTIN*-F CATGTACGTTGCTATCCAGGC, *ACTIN*-R CTCCTTAATGTCACGCACGAT.

### Western blot

For whole-cell lysate preparation, cultured cells were lysed in NP-40 buffer, while tissue samples were homogenized in RIPA buffer supplemented with 100 μM PMSF and 0.1 % (v/v) phosphatase inhibitor (Beyotime Biotechnology, China). After 30 min lysis on ice, samples were centrifuged (14,000 × *g*, 10 min, 4 °C), and supernatants were collected for BCA protein quantification (Bio-Rad). Lysates were mixed with 4× Laemmli buffer and denatured.

For surface protein preparation, cell lysate was performed with Surface and Cytoplasmic Protein Reagent Kit (P0033, Beyotime, Shanghai, China). In brief, cells were washed with PBS and resuspended in membrane extraction reagent A containing 100 μM PMSF. After 15 min incubation on ice, samples were centrifuged (700 × *g*, 10 min, 4 °C). The supernatant was further centrifuged (14,000 × *g*, 30 min, 4 °C) to obtain membrane pellets (solubilized in reagent B). Then the surface protein preparation was mixed with 4× Laemmli buffer and denatured.

For nuclear extraction, cell lysate was performed with Nuclear Protein and Cytoplasmic Protein Extraction Kit (P0027, Beyotime, Shanghai, China). In brief, cells were resuspended in cytoplasmic extraction reagent A (with 100 μM PMSF), incubated on ice for 15 min, then treated with reagent B for 1 min on ice before centrifugation (14,000 × *g*, 10 min, 4 °C). Nuclear pellets were extracted by vortexing in nuclear extraction reagent (30 min, with intermittent mixing), followed by centrifugation to collect nuclear proteins (14,000 × *g*, 10 min, 4 °C). Then the nuclear protein preparation was mixed with 4× Laemmli buffer and denatured.

All the prepared proteins were resolved on 7.5–10% Omni-PAGE™ Hepes Plus gels (Epizyme) and transferred to PVDF membranes (Bio-Rad). After blocking with 5% skim milk in TBST (37 °C, 1 h), membranes were probed with primary antibodies overnight at 4 °C, followed by HRP-conjugated secondary antibodies (37 °C, 1 h). Signals were detected using a ChemiDoc XRS+ system (Bio-Rad) and quantified with Fiji/ImageJ. The antibodies used were shown as followed: VE-Cadherin (ab33168; 1:1000, Abcam), Lamin B (ab16048; 1:5000, Abcam), Sodium/Potassium ATPase alpha 1 (ab308329; 1:1000, Abcam), FLAG (ab205606; 1:10,000, Abcam), ACTIN (ab8226, 1:2000, Abcam), GLUD1 (14299-1-AP; 1:1000, Proteintech), FAK (12636-1-AP; 1:1000, Proteintech), EPHA2 (25749-1-AP; 1:1000, Proteintech), SNAIL (13099-1-AP; 1:1000, Proteintech), β-Tubulin (80713-1-RR; 1:1000, Proteintech), Phospho-FAK (83933-1-RR, 1:1000, Proteintech), Phospho-EPHA2 (25749-1-AP, 1:1000, Proteintech), SOX2 (11064-1-AP, 1:500, Proteintech), SLUG (12129-1-AP, 1:500, Proteintech), ZEB1 (21544-1-AP, 1:500, Proteintech). Importin-8 (PA1-41599; 1:1000, Thermo Fisher), GFP (PA1-980A; 1:1000, Thermo Fisher).

Unless otherwise specified below, immunoblotting samples were analyzed on the same gel. For the immunoblotting experiments listed below, samples were derived from the same experiment, and separate gels were processed in parallel. For Fig. 2A, p-EPHA2 and GLUD1 were analyzed on one gel, whereas p-FAK and β-tubulin were analyzed on another. For Supplementary Fig. 3A, FAK and β-tubulin were analyzed on one gel, whereas EPHA2 and GLUD1 were analyzed on separate gels. For Fig. 2B, E and Supplementary Figs. 1D, 3I, J, M, 5W, 6F, V and 8D, VE-cadherin and GLUD1 from whole-cell lysates were analyzed on one gel, whereas β-tubulin/ACTIN was analyzed on a separate gel; VE-cadherin and Na⁺/K⁺-ATPase from membrane fractions were analyzed on the same gel. For Figs. 3B and 4I and Supplementary Fig. 4D, GLUD1, Lamin B and β-tubulin were analyzed on separate gels. For Fig. 3E, GLUD1 and Importin-8 were analyzed on one gel, whereas Lamin B and β-tubulin were analyzed on separate gels. For Fig. 3F, FLAG-GLUD1 and VE-cadherin from whole-cell lysates were analyzed on one gel, whereas Lamin B, β-tubulin, FLAG-GLUD1 from nucleus fractions were analyzed on separate gels. For Fig. 3M, S, U, the IB: Lamin B/SNAIL blot was obtained from a separate gel from the IP: GLUD1/SNAIL blot. For Fig. 3P and Supplementary Fig. 5X, GLUD1 and SNAIL were analyzed on one gel, whereas β-tubulin and VE-cadherin were analyzed on separate gels. For Figs. 3L and 4J and Supplementary Fig. 8C, the IB: SNAIL blot was obtained from a separate gel from the IP: GLUD1/SNAIL blot. For Figs. 4N, Q, S, T, and 5G, H, J–L and Supplementary Figs. 5I, 6R, S and 8B, the IB: GLUD1/SNAIL blot was obtained from a separate gel from the corresponding IP blot, with IB: Lamin B analyzed on an additional separate gel where indicated. For Supplementary Fig. 5G, ZEB1, SOX2, SLUG, TWIST and GLUD1 were analyzed on separate gels. For Supplementary Fig. 5Y, VE-cadherin and GLUD1 were analyzed on one gel, whereas β-tubulin and SNAIL were analyzed on another.

### Immunofluorescence staining

Cells grown in glass-bottom confocal dishes (50 μg/ml fibronectin was coated onto the glass-bottom in advance for primary HUVEC) were washed with ice-cold PBS, fixed with 4% paraformaldehyde (15 min at room temperature), and permeabilized with 0.3% Triton X-100 in PBS (10 min). After blocking with donkey serum (1.5 h), samples were incubated with primary antibodies overnight at 4 °C, followed by species-matched secondary antibodies (1 h, RT). Nuclei were counterstained with DAPI (Beyotime, #C1005). Confocal images were acquired using an FV3000 microscope (Olympus) and analyzed with Fiji/Image J. The used antibodies were shown as followed: VE-Cadherin (ab33168; 1:300, Abcam), GLUD1 (67026-1-lg; 1:300, Proteintech), SNAIL (13099-1-AP; 1:200, Proteintech), Hsp60 (HY-P80184, 1:200, MedChemExpress).

### Tumor cell trans-endothelial migration based on 2D model

For adhesion assays, primary HUVECs (1 × 10⁵ cells/well) were seeded in 48-well plates and cultured for 48–72 h (37 °C, 5% CO₂) to form tight junctions. Tumor cells (2.5 × 10⁵ cells/well) were labeled with 5 μM CellTrace™ CFSE (Thermo Fisher, #C34570; 30 min, 37 °C), while confluent primary HUVECs were labeled with 5 μM CellTrace™ Violet (Thermo Fisher, #C34571). Labeled tumor cells were co-cultured with HUVECs (30 min, 37 °C), washed with PBS (3×), and imaged (BioTek Lionheart FX). Adhesion was quantified using Fiji/ImageJ.

For barrier-destruction assays, primary HUVECs (1 × 10^4^ cells/well) were cultured in glass-bottom confocal dishes for 24–48 h to establish junctions. CFSE-labeled tumor cells (5 × 10³ cells/well) were added for 30–120 min (37 °C), washed (PBS 3×), fixed (4% PFA, 15 min), and immunostained for VE-Cadherin (Abcam ab33168; 1:300). Confocal images (Olympus FV3000) were analyzed for junction disruption.

For transmigration assays, Transwell inserts (Corning) were coated with rat tail collagen I (50 μL, 354236). HUVECs (5×10³ cells/insert) were seeded to form confluent monolayers. Tumor cells (5 × 10³ cells/insert in serum-free ECM) were added to the upper chamber, with complete ECM (+5% FBS) in the lower chamber. After 48 h (37 °C, 5% CO₂), invaded cells were fixed and stained with 0.1% crystal violet (Keygene, #KGE1202-100). Membranes were imaged (BioTek Lionheart FX) and quantified (Fiji).

### Physiologically relevant 3D vascular model

We developed a physiologically relevant 3D vascular model using the OrganoPlate 3-lane platform (MIMETAS, Netherlands). The ECM channel was prepared by mixing neutralized rat tail collagen I (Corning) with 1 M HEPES and NaHCO₃ (1:1:8 ratio) on ice, then loaded (2–5 μL) into lane 3 of a pre-cooled OrganoPlate 3-lane (MIMETAS). After polymerization (37 °C, 15 min), 50 μL HBSS was added. Then primary HUVECs (*P *≤ 5, 80% confluency) were trypsinized, resuspended (1 × 10⁷ cells/mL), and seeded (2 μL) into lane 1. Following medium addition (50 μL) and tilting (75°, 25 min), plates were cultured on an OrganoFlow rocker (7°, 8 min intervals) with medium changes every 24-48 h. Lumen formation was monitored by live imaging from day 2.

To assess barrier function, 20 kDa FITC-dextran and 155 kDa TRITC-dextran were perfused through the top channel (lanes 1–2) of established vascular lumens in OrganoPlate while imaging GFP/RFP channels (Lionheart FX, 2 min intervals for 20 min). Exposure settings were calibrated using cell-free controls to achieve a gel/perfusion channel fluorescence ratio ≈1 at 20 min. After removing medium from all ports, systems were equilibrated (7° tilt, 8 min intervals) before adding dye solutions (40 μL inlet/30 μL outlet, top channel) and 20 μL serum-free medium to other ports. Permeability coefficients (Papp, cm/s) were calculated from time-dependent fluorescence intensity ratios (*I*_gel_/*I*_perfusion_) in Fiji using

$${P}_{{app}}=\frac{d(\frac{{I}_{{gel}}}{{I}_{{perfusion}}})}{{dt}}\times \,\frac{{V}_{{gel}}}{{A}_{{barrier}}}$$

where *V*_gel_ is gel volume (cm³) and *A*_barrier_ is lumen-gel contact area (cm²). Post-assay, channels were washed (3× with endothelial medium) for continued culture.

### Tumor cell trans-endothelial migration based on a 3D model

To model tumor intravasation, GFP-tagged tumor cells (1 × 10⁷ cells/mL in collagen I matrix) were seeded into the middle channel of OrganoPlate 3-lane and polymerized (37 °C, 15 min). HUVECs (*P* ≤ 5) were trypsinized, resuspended in endothelial medium (1 × 10⁷ cells/mL), and seeded (2 µL) into the top perfusion channel. After tilting (75°, 25 min) for endothelial attachment, both channels were perfused with respective media and cultured under dynamic conditions (OrganoFlow rocker: 7° tilt, 8 min intervals) with medium changes every 24–48 h. Tumor-endothelial interactions were monitored daily by phase-contrast/GFP imaging until day 5, when samples were fixed for immunofluorescence analysis.

To model tumor extravasation, HUVECs were cultured in the top channel of OrganoPlate 3-lane for 4 days to form stable vascular networks, with barrier function verification. GFP-tagged tumor cells (1 × 10⁶ cells/mL in endothelial medium) were introduced into the vascular lumen (2 µL via inlet port), allowed to settle (75° tilt, 30 min), and perfused with fresh medium. Tumor-endothelial interactions were monitored by time-lapse imaging (GFP/RFP channels) at 12, 24, and 36 h post-seeding using live-cell microscopy.

### Co-immunoprecipitation (Co-IP) assay

For cellular protein studies, nuclear and whole-cell extracts from A549/DTX cells were prepared using commercial kits (Nuclear Protein Extraction Kit, #P0027; Cell Lysis Buffer, #P0013; Beyotime). Lysates (5 μg/μL) were aliquoted into input (30 μL) and immunoprecipitation (IP) samples. IP samples were incubated overnight at 4 °C with either target antibodies (anti-GLUD1, #14299-1-AP, Proteintech; anti-SNAIL, #13099-1-AP, Proteintech) or control IgG (#A7016, Beyotime), followed by 4 h incubation with Protein A/G magnetic beads (#HY-K0202, MCE). Beads were washed (3× PBS) and bound proteins eluted in 4× Laemmli buffer (95 °C, 5 min).

For recombinant protein interactions, tagged proteins (GST/FLAG/GFP/Biotin, 5 μg) were incubated (2 h, RT) before IP with respective antibodies: anti-FLAG (#ab205606, Abcam), anti-GFP (#PA1-980A, Thermo Fisher), or streptavidin/GST magnetic beads (#P2151/P2138, Beyotime). Input (20 μL) and IP samples were processed as above.

All samples were resolved by SDS-PAGE, transferred to PVDF membranes, and probed with appropriate antibodies. Signals were detected by chemiluminescence.

### Chromatin-immunoprecipitation (Ch-IP) assay

Chromatin immunoprecipitation was performed using the Pierce™ Magnetic ChIP Kit (Thermo Fisher Scientific, #26157) with the following modifications:

For cellular chromatin, tumor cells (1 × 10⁷) were crosslinked with 1% formaldehyde (10 min, RT) and quenched with 0.125 M glycine. Chromatin was extracted by nuclear separation and sheared by MNase/sonication to 200–500 bp fragments and immunoprecipitated overnight at 4 °C with rotation using different antibodies and species-matched IgG controls.

For recombinant chromatin, annealed DNA sequences were incubated with recombinant proteins (2 h, 37 °C) before antibody addition and overnight IP at 4 °C.

Protein A/G magnetic beads were added (4 h, 4 °C), followed by sequential washes and elution. For sequential ChIP (reChIP), eluates were subjected to secondary IP. Crosslinks were reversed (65 °C, 1.5 h with Proteinase K) and DNA purified using kit columns.

Quantitative PCR was performed with the following primers (5’–3’): SNAIL binding site (Motif1) within *CDH5* promoter F-TGTGATGCTGCAGCCAAG, R-AGTCCCAGAGCTGAGGCCGGG; Motif2 within *CDH5* promoter F- AGATTCCCAGGATCTGCCCT, R- GAAGGGAGGGGCCAAGATTC; Motif3 within *CDH5* promoter F- CCTTGTGAGGATGAGGCTGT, R- GGAGTCAAGTGACCCAGCTC; Motif4 within *CDH5* promoter F- AATGTGATGCTGCAGCCAAG, R- CCGGATGGCTGTCTCCAC; TWIST binding site within *CDH5* promoter: F-CTTGTGAGGATGAGGCTGTGA, R-CACTTGACTCCACCCAATATCCCT; SOX2 binding site within *CDH5* promoter: F-CCAGCCCTCTGTGGAGAC, R-CAATAACAGGAAACCATCCCAGG; SLUG/ZEB1 binding site in *CDH5* promoter: F-ACCCCAGCTAGATTTTTGGC, R-TTCCAAGGAGGGTTAAA.

### Immunoprecipitation coupled with proteomics analysis

Nuclear protein extracts from tumor cells were prepared using a nuclear and cytoplasmic protein extraction kit (Beyotime #P0027). Protein concentrations were determined using a BCA assay and adjusted to 5 μg/μL in PBS containing 100 μM PMSF. Nuclear lysates were incubated with 5 μg anti-GLUD1 antibody or an equivalent amount of control IgG overnight at 4 °C, followed by incubation with magnetic beads for 4 h. The beads were washed three times with PBS, and bound proteins were eluted in loading buffer at 95 °C for 5 min. Samples were separated on 10% Omni-PAGE Hepes Plus gels, and the excised gel regions were subjected to bottom-up proteomic analysis by APT Biotechnology. Three independent immunoprecipitation experiments were pooled to generate one LC-MS/MS sample per condition.

### Conditional DNA pull-down coupled with proteomics analysis

The target promoter sequence (1000 bp upstream of exon) was retrieved from the NCBI database (https://www.ncbi.nlm.nih.gov/) and synthesized with 5’ biotin modification (FITGENE, China). Tumor cells were lysed under two conditions: (1) SDS lysis buffer with sonication (strong condition) or (2) IP lysis buffer (mild condition). After centrifugation at 14,000 × *g* for 10 min at 4 °C, the clarified lysates were incubated with the biotinylated DNA probe overnight at 4 °C. DNA-protein complexes were captured using streptavidin magnetic beads for 30 min at room temperature. Bound proteins were eluted in 4× Laemmli buffer at 95 °C for 7 min, separated on 10% Omni-PAGE Hepes Plus gels and subjected to bottom-up proteomic analysis by APT Biotechnology. Three independent DNA pull-down experiments were pooled to generate one LC-MS/MS sample per condition.

### LC-MS/MS acquisition and database searching

Excised gel samples were reduced and alkylated and digested with sequencing-grade trypsin at an enzyme-to-protein ratio of 1:50 (w/w) for 20 h at 37 °C. Peptides were desalted, lyophilized and reconstituted in 0.1% formic acid. Nano-LC-MS/MS analysis was performed using an Easy-nLC 1000 system coupled to a Q Exactive mass spectrometer (Thermo Fisher Scientific) in positive-ion mode. Mobile phase A consisted of water containing 0.1% formic acid, and mobile phase B consisted of 84% acetonitrile containing 0.1% formic acid. The LC system was equilibrated with 95% mobile phase A. Peptides were loaded onto an Acclaim PepMap 100 trap column (100 μm × 2 cm, nanoViper C18; Thermo Fisher Scientific) and separated on an EASY analytical column (25 cm × 75 μm inner diameter, 1.9 μm particle size; Thermo Fisher Scientific) at a flow rate of 300 nL min^−1^. The 60-min gradient of mobile phase B was as follows: 5–8% from 0 to 3 min, 8–26% from 3 to 43 min, 26–40% from 43 to 48 min, 40–90% from 48 to 50 min, followed by maintenance at 90% from 50 to 60 min.

Full MS scans were acquired over an m/z range of 300–1800 at a resolution of 70,000 at m/z 200, with an automatic gain control target of 1 × 10⁶ and a maximum injection time of 50 ms. The 20 most intense precursor ions from each full scan were selected for higher-energy collisional dissociation using a normalized collision energy of 27. MS/MS scans were acquired at a resolution of 17,500 at m/z 200 with an isolation window of 2 m/z. Dynamic exclusion was set to 30 s, and the underfill ratio was 0.1%. Raw files were processed using MaxQuant version 1.6.14 and searched against the UniProt Homo sapiens protein sequence database downloaded on 8 January 2024. Trypsin specificity was applied, allowing up to two missed cleavages. Carbamidomethylation of cysteine was specified as a fixed modification, and oxidation of methionine was specified as a variable modification. The precursor-ion and fragment-ion mass tolerances were 20 ppm and 0.1 Da, respectively. The false-discovery rate was controlled at 1% at both the peptide and protein levels, and intensity-based absolute quantification was enabled. The minimum peptide length was seven amino acids. No additional minimum unique-peptide filter was applied after MaxQuant processing, and all protein groups passing the specified false-discovery-rate threshold were retained.

### Microscale thermophoresis (MST) binding assay

Binding affinities were determined using Monolith NT.115 (NanoTemper Technologies). GFP-tagged recombinant proteins were mixed with 16 serially diluted concentrations (typically spanning 1 nM to 1 mM) of α-Ketoglutarate (α-ketoglutarate, #K105570, Aladdin, China) and Glutarate (#D154294, Aladdin, China)

### Cellular thermal shift assay (CETSA)

We performed thermal stability profiling using an optimized CETSA protocol. Tumor cells (1 × 10⁸) were harvested in PBS containing 100 μM PMSF and protease inhibitors, then aliquoted into eight PCR tubes for temperature gradient treatment (37 °C, 38.5 °C, 41.3 °C, 45.7 °C, 51 °C, 55.1 °C, 58.1 °C, 60 °C; 3 min each) using a thermal cycler. Following 3 min equilibration at room temperature, samples underwent three freeze-thaw cycles (alternating liquid nitrogen and 25 °C water bath) before centrifugation (20,000 × *g*, 20 min, 4 °C). Soluble fractions (90 μL supernatant) were collected for western blotting.

### Drug affinity responsive target stability (DARTS) assay

The DARTS assay was performed to assess protein-ligand interactions through protease sensitivity profiling. Recombinant GLUD1 (50 ng) was incubated with 5 mM α-ketoglutarate (α-KG) in 20 mM HEPES buffer (pH 7.4) containing 1 mM DTT for 15 min at room temperature. Proteolytic digestion was then carried out using subtilisin (Sigma-Aldrich, #P4860) at final concentrations of ~0.4, 0.2 and 0.1 μg/mL for 30 min at 25 °C. Reactions were terminated by adding 4× Laemmli buffer and heating at 95 °C for 5 min. Finally, separation was performed by Western Blot.

### Targeted LC-MS/MS determination of nuclear α-ketoglutarate

Nuclear α-ketoglutarate (α-KG) was quantified using a targeted LC-MS/MS method. A total of three independent biological samples was analyzed per group. Cells were collected in ice-cold 0.25 M sucrose buffer (containing 10 mM Tris-HCl and 3 mM MgCl_2_, pH 7.4) and homogenized using a pre-cooled homogenizer. The homogenate was filtered and layered onto a 0.34 M sucrose cushion, followed by centrifugation at 700 × *g* for 10 min at 4 °C to pellet the nuclei. The nuclear pellet was washed twice with 0.25 M sucrose buffer at 1000 × *g* for 10 min to minimize cytosolic contamination. To ensure metabolic quenching and prevent α-KG leakage, the final nuclear pellet was immediately extracted with 500 μL of pre-chilled 80% (v/v) methanol at −80 °C for 2 hours. After centrifugation at 15,000 × *g* for 15 min, the supernatant was collected and vacuum-dried for subsequent LC-TRAP-MS/MS analysis. Chromatographic separation was performed on a Shimadzu LC-20 HPLC system (Kyoto, Japan) coupled to a QTRAP 5500 mass spectrometer (AB Sciex, Framingham, MA, USA).

Chromatographic separation was performed using a liquid chromatography system equipped with an XSelect HSS T3 column (150 × 4.6 mm, 3.5 μm; Waters, Milford, MA, USA) at 40 °C on the Shimadzu HPLC. The mobile phase consisted of solvent A (5 mM ammonium acetate, pH = 9, and 5% acetonitrile) and solvent B (acetonitrile) with the following gradient:0 min, 5% B; 5 min, 5% B; 5.5 min, 75% B; 7.5 min, 75% B; 8 min, 5% B with a flow rate of 0.5 mL/min.

α-KG was detected in negative electrospray ionization mode using multiple reaction monitoring (MRM). The ESI source conditions were set as follows: Gas1, 33 psi; Gas2, 33 psi; Curtain Gas, 25 psi; Ion spray voltage, −4500 V; Turbo spray temperature, 550 °C. The MRM transition for α-KG was m/z 145.1 → 101.0, with declustering potential (DP) = −20 V and collision energy (CE) = −12 V. 2-Ketoglutaric acid-^13^C_5_ was used as an isotopically labeled internal standard and The MRM transition for Ketoglutaric acid-^13^C_5_ was m/z 151.06 → 105.0, with declustering potential (DP) = −50 V and collision energy (CE) = −12 V. Data acquisition and analysis were both performed using Analyst v1.5.1 software. Quantification was by comparison with an external standard curve generated using authentic α-KG standards. Further nuclear α-KG concentration was calculated based on the measured α-KG amount normalized to the estimated nuclear fraction volume (1 pL per cell).

### MD simulation

Protein structures were predicted using AlphaFold (GLUD1: P00367; SNAIL:O95863). The α-KG structure was retrieved from PubChem (CID:51). Molecular docking was performed using Molecular Operating Environment (MOE) software, generating 30 ligand-protein conformations ranked by the MOE scoring algorithm. The top-scoring conformation was selected for molecular dynamics (MD) simulations. All simulations were conducted using Gromacs 2022 with the following parameters: Force fields, Amber99sb-ildn (protein) and GAFF (ligand); Solvation, SPCE water model in a 10 × 10 × 10 nm³ box (minimum 1.2 nm protein-to-edge distance); Electrostatics, Particle-Mesh Ewald (PME) method with 1.0 nm cutoff; Energy minimization: 50,000 steps using steepest descent algorithm; System equilibration: NVT and NPT ensembles (300 K, 1 bar) using V-rescale and Berendsen coupling methods; Production run: 100 ns MD simulation at 300 K and 1 bar with 10 Å non-bonded interaction cutoff. Trajectory analysis was performed using Gromacs-built-in tools, with visualizations created in PyMOL (v2.5.0).

### Public bioinformatics dataset analysis

Publicly available datasets were obtained from the following sources: TCGA PanCancer Atlas (lung adenocarcinoma, melanoma, thymoma, and prostate cancer; https://xenabrowser.net), GEO (NSCLC single-cell RNA-seq: GSE131907; melanoma single-cell RNA-seq: GSE215121; https://www.ncbi.nlm.nih.gov/geo/), Kaplan-Meier Plotter (overall/progression-free survival analysis; https://kmplot.com), and MetMap (metastasis quantification; https://depmap.org/metmap/).

None of the databases applied sex-based screening. Both female and male patients are included. In the TCGA dataset, the lung adenocarcinoma cohort comprised 357 females and 301 males; the melanoma cohort comprised 180 females and 296 males; the thyroid carcinoma cohort comprised 423 females and 157 males; and the prostate cancer cohort (which inherently includes only male patients) comprised 572 males; and the breast cancer cohort included 1241 females and 13 males (which mainly occurs in females). In the GEO single-cell datasets, the lung adenocarcinoma cohort included 17 females and 27 males; the melanoma cohort included 3 females and 5 males. The in-house cohort related to Fig. 1D, E included 11 females and 8 males.

### Statistics and reproducibility

Statistical analyses were performed using GraphPad Prism 10.0 and R v4.3.2. Data are presented as mean ± SEM unless otherwise indicated. Exact sample sizes and the definition of n are provided in the corresponding figure legends. No statistical method was used to predetermine sample size. No data were excluded from the analyses. Comparisons between two groups were performed using two-sided unpaired Student’s t-tests. Comparisons among multiple groups were analyzed using one-way or two-way ANOVA. For single-factor multigroup comparisons, one-way ANOVA with Tukey’s or Dunnett’s post hoc testing was applied. For experiments involving two independent variables, two-way ANOVA followed by Sidak’s multiple-comparisons test was used. Categorical outcomes, including the incidence of metastasis-associated hindlimb paralysis, were compared using a two-sided Fisher’s exact test. Correlations were evaluated using two-sided Pearson’s correlation. Survival analyses were performed using the two-sided log-rank test. Details of the statistical methods for each figure are included in the respective figure legends, with significance set at *P* < 0.05. All experiments corresponding to the representative micrographs shown in the figures were independently repeated more than three times.

### Reporting summary

Further information on research design is available in the Nature Portfolio Reporting Summary linked to this article.

## Supplementary information

Supplementary information

Description of Additional Supplementary Files

Supplementary Data 1

Supplementary Data 2

Supplementary Data 3

Reporting Summary

Transparent Peer Review file

## Source data

Source data

## Data Availability

The TCGA datasets analysed in this study are available through the UCSC Xena Browser (https://xenabrowser.net/). The publicly available lung cancer and melanoma single-cell RNA-sequencing datasets are available from the Gene Expression Omnibus under accession codes GSE131907 and GSE215121, respectively. The IP-mass spectrometry data related to Supplementary Fig. 4G generated in this study have been deposited in the ProteomeXchange Consortium via the iProX partner repository under accession code PXD075031. The DNA pull-down coupled mass spectrometry data related to Fig. 3G have been deposited in the ProteomeXchange Consortium via iProX under accession code PXD075033. The targeted LC-MS/MS data for nuclear α-ketoglutarate quantification related to Fig. 4O have been deposited in MetaboLights under accession code MTBLS15115. Source data are provided with this paper.

## Code availability

No custom code was developed for this study.
