# Supplementary material for: α-KG conformationally stabilizes nuclear GLUD1-SNAIL complex to fuel trans-endothelial migration in metastasis
Source: Nat Commun. 2026 Aug 31;17:10377. doi: 10.1038/s41467-026-77149-0 (PMC13627656; doi:10.1038/s41467-026-77149-0)
Supplement: Supplementary file 1 [file 41467_2026_77149_MOESM1_ESM.pdf]

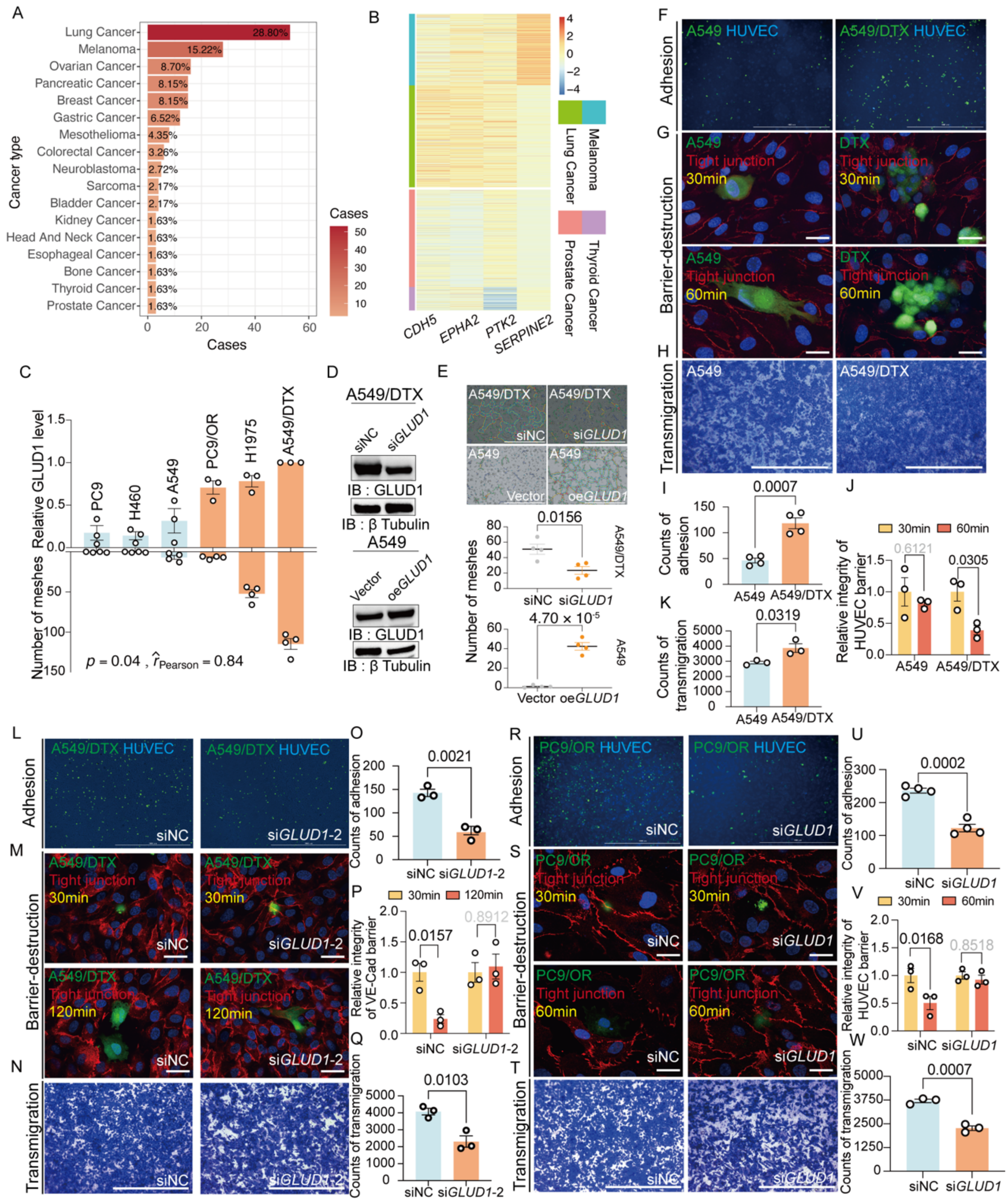

Supplementary Fig. 1 GLUD1 promotes tumor trans-endothelial migration. **Related to Fig. 1.** (A) Incidence of metastasis from primary tumors (Data source: https://depmap.org/metmap/). (B) Heatmap of VM marker genes (CDH5, EPHA2, PTK2, SERPINE2) expression across four cancer types (Data source: TCGA). (C) GLUD1 protein levels (top, n=3 biologically independent samples) and tube formation capacity (bottom, n=4 biologically independent samples) across NSCLC cell lines (A549/DTX as control). The relative GLUD1 levels were corrected by β-tubulin. (D-E) GLUD1 protein level (D, representative of three independent experiments) and tube formation capability (E) in A549/DTX (si*GLUD1* vs. control) and A549 (oe*GLUD1* vs. control), images were processed with FIJI Angiogenesis Analyzer2, n=4 biologically independent samples. (F-K) Representative immunofluorescence images (F-H) and quantification (I-K) of adhesion (F and I,

n=4 biologically independent samples), barrier-destruction (G and J, n=3 biologically independent samples) and transmigration (H and K, n=3 biologically independent samples) for A549 and A549/DTX cells. For adhesion and transmigration quantification, cells were counted by FIJI. For barrier-destruction quantification, the immunofluorescence of tight junction is determined by FIJI and normalized to the 30-minute time point within each group. (L-Q) Representative immunofluorescence images (L-N) and quantification (O-Q) of adhesion (L and O, n=3 biologically independent samples), barrier-destruction (M and P, n=3 biologically independent samples) and transmigration (N and Q, n=3 biologically independent samples) for siNC or si*GLUD1*-2-treated A549/DTX cells. (R-W) Representative immunofluorescence images (R-T) and quantification (U-W) of adhesion (R and U, n=4 biologically independent samples), barrier-destruction (S and V, n=3 biologically independent samples) and transmigration (T and W, n=3 biologically independent samples) for siNC or si*GLUD1*-treated PC9/OR cells. All data are mean ± SEM. P values were calculated using two-sided Pearson correlation analysis (C); two-tailed unpaired Student's t-test (E,I,K,O,Q,U,W); two way ANOVA followed by Šídák multiple-comparisons test (J,P,V). Scale bars: E,F,H,L,N,R,T 1000 μm; G,M,S, 20 μm. Source data are provided as a Source Data file. For immunoblots, samples within each panel were derived from the same experiment, with separate gels processed in parallel where applicable; detailed gel assignments are provided in the Methods.

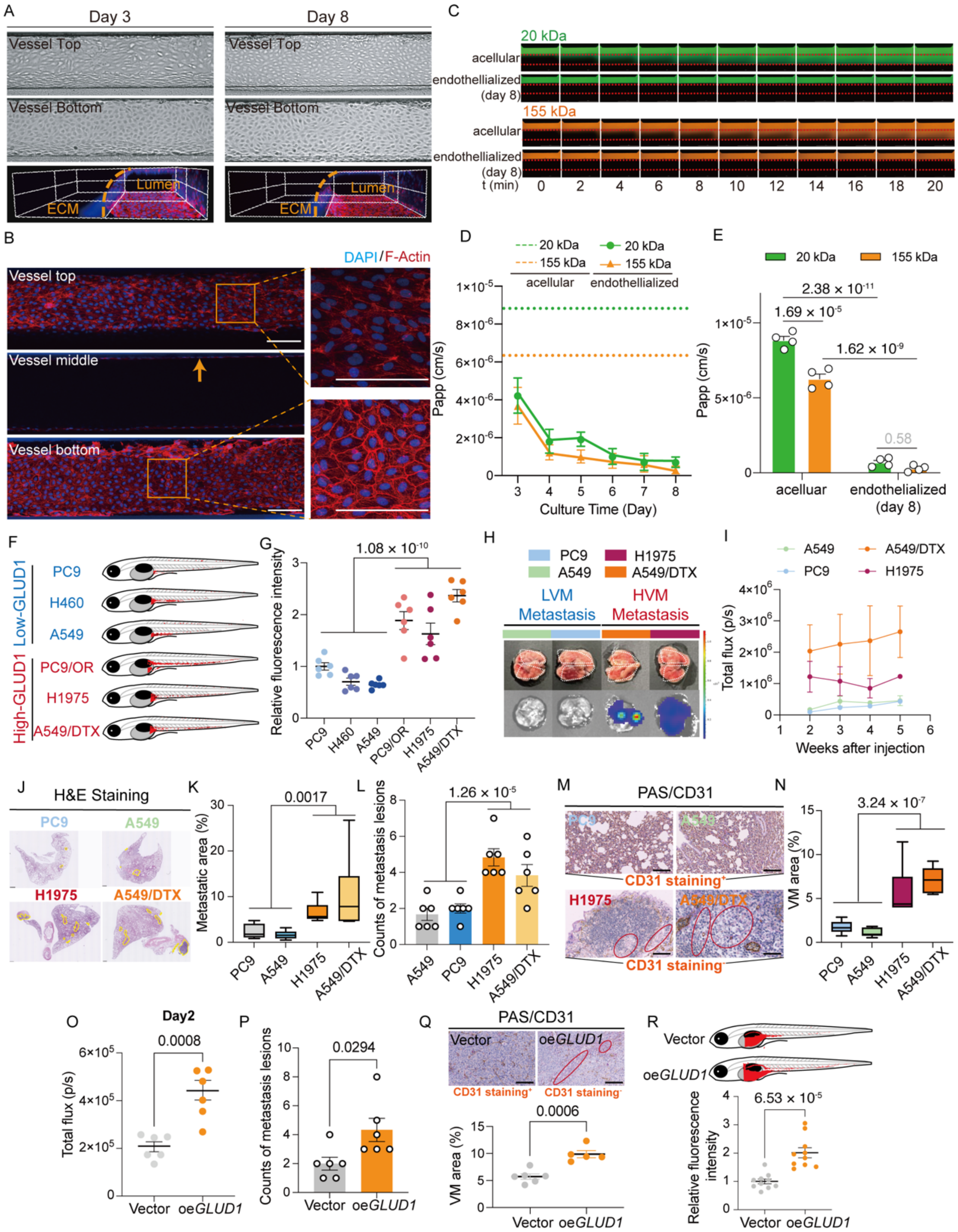

Supplementary Fig. 2 GLUD1 drives metastatic progression across multiple *in vivo* models. Related to Fig. 1. (A) Validation of vascular-network formation in 3-lane microfluidic model. Brightfield images show distinct cellular layers, and DAPI/CD31 immunofluorescence shows 3D-reconstructed luminal structures on days 3 and 8. Representative images from four biologically independent samples are shown. (B) Tight-junction integrity of day-8 vascular networks assessed by phalloidin staining and multilayer fluorescence imaging. Representative images from four biologically independent samples are shown. (C) Dextran-permeability assay showing penetration of 20- and 155-kDa dextran through day-8 vascular networks, imaged every 2 min for 30 min. Representative images from four biologically independent samples are shown. (D) Time-course

analysis of apparent permeability (Papp) values during 3D vascular maturation (unseeded channels as negative controls), n=4 biologically independent samples. (E) Comparative Papp values at culture endpoint (Day 8), n=4 biologically independent samples. (F-G) Metastatic potential (F) and quantification (G) of six cell lines in intravasation based metastasis zebrafish model (n=6 embryos per group). (H-I) Bioluminescence imaging (H) and quantification (I) of metastatic burden in extravasation based metastasis murine model (n=5 mice per group). (J-K) H&E staining (J) of metastatic lung lesions from (H) and quantitative analysis shown in K (metastatic area/total lung area; n=6 mice per group). (L) Counts of lung metastasis lesions related to Supplementary Fig. 2H (n=6 mice per group). (M-N) VM formation (PAS+/CD31-) in lung metastases (M) and quantitative analysis (N) related to Supplementary Fig.2H (n=6 mice per group). (O) Quantification of bioluminescence on metastasis murine model injected GLUD1-expressing A549 in day 2 related to Fig.1R, n = 6 mice per group. (P) Counts of lung metastasis lesions related to Fig.1R (n=6 mice per group). (Q) VM formation (PAS+/CD31-) in lung metastases (above) and quantitative analysis (below) related to Fig.1R. VM levels were calculated as the mean of five $PAS^+/CD31^-$ fields of view per mouse at ×20 magnification, with each data point representing one biologically independent mouse (n = 6 mice per group). (R) Relative quantification of fluorescence intensity of migrating cells in A549 cells transfected with Vector/oe*GLUD1* plasmid within perivitelline space (PVS) zebrafish xenograft model, n = 10 embryos per group. All data are mean ± SEM. P values were calculated using two-tailed unpaired Student's t-test (G,K,L,N,O,P,R); two way ANOVA followed by Šídák multiple-comparisons test (E). Scale bars: B,M,Q, 100 μm; J, 500 μm. Source data are provided as a Source Data file.

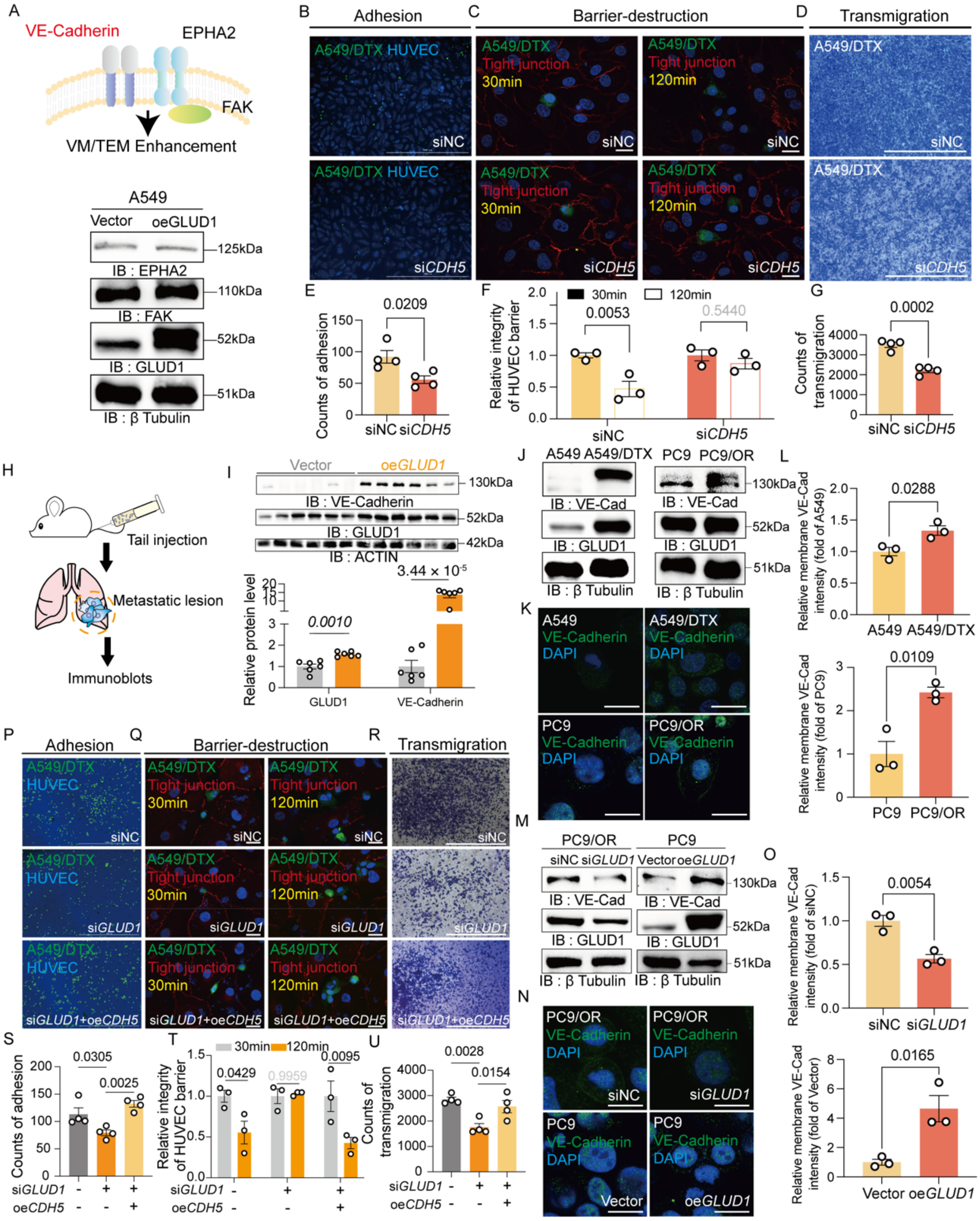

Supplementary Fig. 3. VE-Cadherin is regulated by GLUD1. Related to Fig. 2. (A) Protein levels of EPHA2 and FAK in A549 (oe*GLUD1* versus vector). (B-G) Representative immunofluorescence images (B-D) and quantification (E-G) of adhesion (B and E, n=4 biologically independent samples), barrier-destruction (C and F, n=3 biologically independent samples) and transmigration (D and G, n=3 biologically independent samples) for A549/DTX cells following *CDH5* knockdown (si*CDH5* versus siNC). (H) Sketchmap of extracting mice metastatic lesions from extravasation based murine models. (I) VE-Cadherin and GLUD1 protein expression and quantification in metastatic lesions of mice model, n=6 biologically independent samples. (J) Immunoblot of VE-Cadherin protein in high-VM cells (A549/DTX and PC9/OR) versus paired low-VM cells (A549 and PC9). Representative images from three independent experiments are shown.

(K-L) VE-Cadherin immunofluorescence (K) and membrane quantified intensity (L) in high-VM cells (A549/DTX and PC9/OR) versus paired low-VM cells (A549 and PC9). The average fluorescence intensity of VE-Cadherin on the cell membrane surface was calculated by manually selecting the area using FIJI and normalized to A549/PC9 group, respectively, n=3 biologically independent samples. (M) Immunoblot of VE-Cadherin protein in PC9/OR transfected with siNC or si*GLUD1* and PC9 transfected with Vector and oe*GLUD1* plasmid. Representative images from three independent experiments are shown. (N-O) Corresponding VE-Cadherin immunofluorescence (N) and membrane quantified intensity (O). The average fluorescence intensity of VE-Cadherin on the cell membrane surface was calculated by manually selecting the area using FIJI and normalized to siNC/Vector group, respectively, n=3 biologically independent samples. (P-U) Representative immunofluorescence images (P-R) and quantification (S-U) of adhesion (P and S, n=4 biologically independent samples), barrier-destruction (Q and T, n=3 biologically independent samples) and transmigration (R and U, n=3 biologically independent samples) for A549/DTX cells following *GLUD1* knockdown and *CDH5* overexpression. All data are represented as mean ± SEM. P values were calculated using two-tailed unpaired Student's t-test (E,G,I,L,O); one way ANOVA followed by Dunnett's multiple-comparisons test (S,U); two-way ANOVA followed by Šídák multiple-comparisons test (F,T). Scale bars: B,D,P,R 1000 µm; C,K,N,Q 20 µm. Source data are provided as a Source Data file. For immunoblots, samples within each panel were derived from the same experiment, with separate gels processed in parallel where applicable; detailed gel assignments are provided in the Methods.

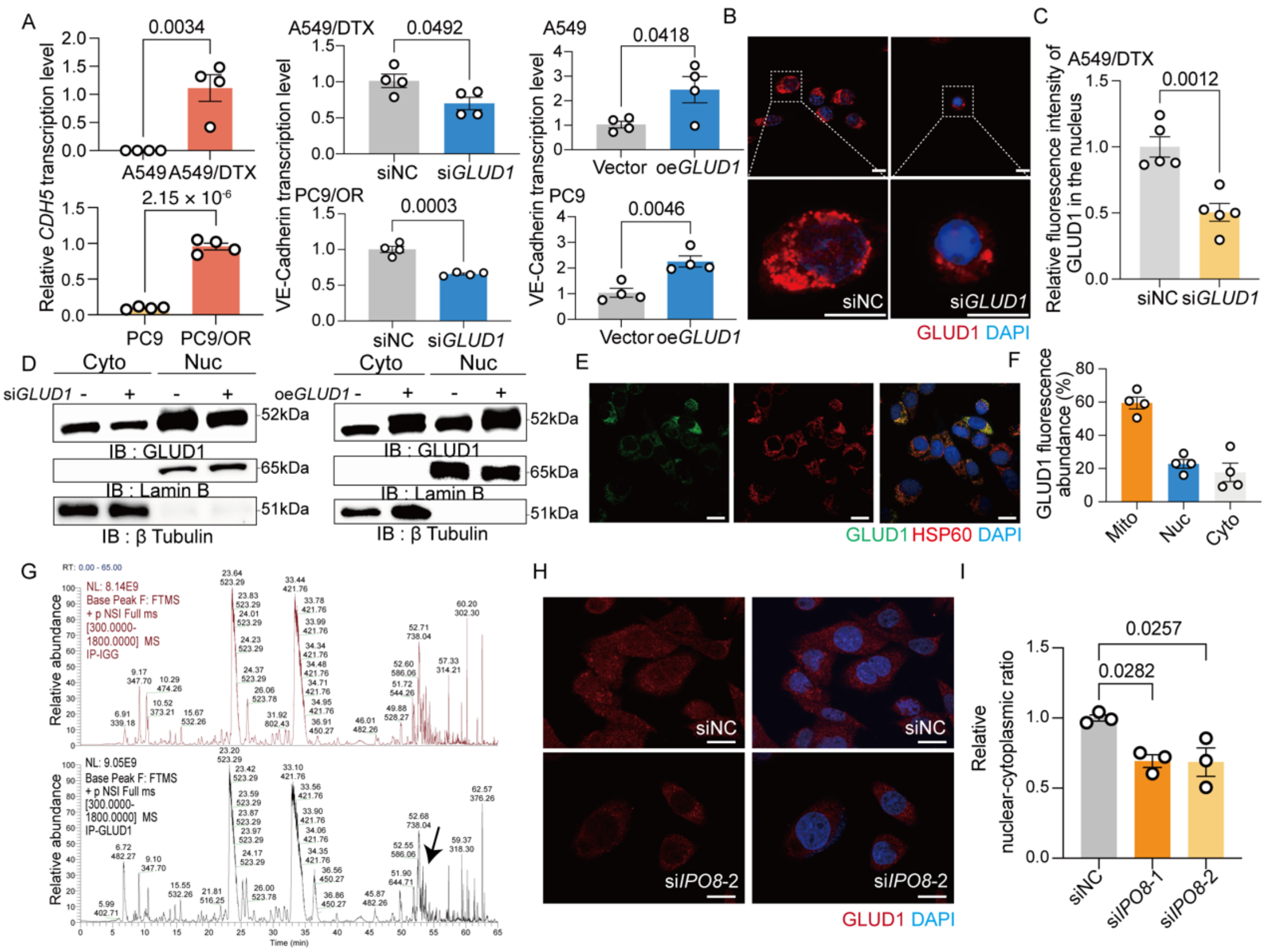

Supplementary Fig. 4 GLUD1 translocates into nucleus by interacting with Importin-8 and its Nuclear Localization Sequence (NLS). Related to Fig. 3. (A) *CDH5* mRNA levels in HLM/LVM cells (left) and A549/DTX, PC9/OR transfected with siNC or si*GLUD1* (middle) and A549, PC9 transfected with Vector and oe*GLUD1* plasmid (right), n=4 biologically independent samples. (B-C) Immunofluorescence analysis (B) of GLUD1 localization with quantification of relative fluorescence intensity (C). DAPI area was selected as the area for GLUD1 fluorescence to be measured and data has been normalized to siNC group, n=4 biologically independent samples. (D) Subcellular GLUD1 distribution in A549/DTX cells following *GLUD1* knockdown (left) and A549 cells with *GLUD1* overexpression (right). Representative images from three independent experiments are shown. (E-F) Immunofluorescence analysis (E) of GLUD1 localization with quantification of relative fluorescence intensity (F). DAPI area was selected for nucleus, HSP60 area was selected for mitochondria and the total area minus nucleus and mitochondria was selected for cytosol using FIJI, n=4 biologically independent samples. (G) IP-MS spectrum showing GLUD1-interacting proteins (above) with IgG pull down as control (below). (H-I) Representative immunofluorescence images and quantification of relative fluorescence nuclear-cytoplasm ratio of A549/DTX treated with siNC/si*IPO8* related to Fig.3D , n=3 biologically independent samples. All data are represented as mean ± SEM. P values were calculated using two-tailed unpaired Student's t-test (A,C); one way ANOVA followed by Dunnett's multiple-comparisons test (I). Scale bars: B,E,H, 20 µm. Source data are provided as a Source Data file. For immunoblots, samples within each panel were derived from the same experiment, with separate gels processed in parallel where applicable; detailed gel assignments are provided in the Methods.

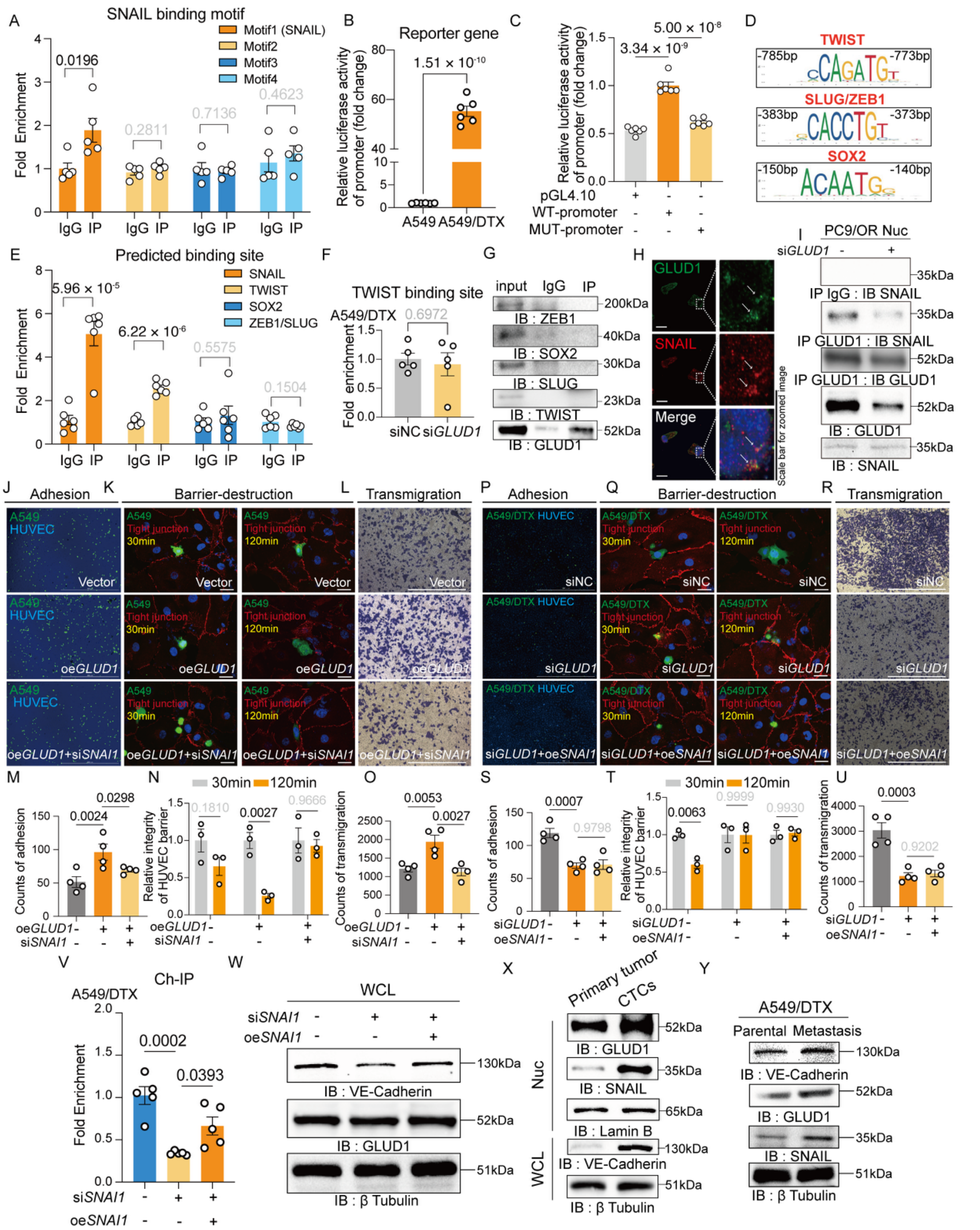

Supplementary Fig. 5 GLUD1 transactivates VE-Cadherin by interacting with SNAIL thereby binding to *CDH5* promoter. Related to Fig. 3. (A) GLUD1 binding to top 4 predicted motifs of SNAIL in A549/DTX, n=5 biologically independent samples. Motif1:TCCAGGTGCT, Motif2: TCAGTTG, Motif3: TTCAAGTGGG , Motif4: GGGAAGTGGG. (B) *CDH5* promoter activity in A549 versus A549/DTX with dual-luciferase assay, n=6 biologically independent samples. (C) Activity of wild-type and mutant *CDH5* promoters in A549/DTX cells, n=6 biologically independent samples. (D) JASPAR-predicted SNAIL, TWIST, ZEB1/SLUG and SOX2 sites in the *CDH5* promoter. (E) GLUD1 binding affinity to predicted sites of SNAIL, TWIST, SLUG, ZEB1/SOX2 with ChIP assay, n = 6 biologically independent samples. (F) GLUD1 binding

affinity to predicted sites of TWIST in A549/DTX with si*GLUD1* treatment versus controls, n=5 biologically independent samples. (G) Interactions between GLUD1 and ZEB1/SOX2/SLUG/TWIST with co-IP assay. Representative images from three independent experiments are shown. (H) GLUD1-SNAIL nuclear co-localization in A549/DTX. (I) Nuclear complex in PC9/OR with si*GLUD1* treatment. Representative images from three independent experiments are shown. (J-O) Representative immunofluorescence images (J-L) and quantification (M-O) of adhesion (J and M, n=4 biologically independent samples), barrier-destruction (K and N, n=3 biologically independent samples) and transmigration (L and O, n=4 biologically independent samples) for A549 cells after *GLUD1* overexpression and *SNAI1* knockdown. (P-U) Representative immunofluorescence images (P-R) and quantification (S-U) of adhesion (P and S, n=4 biologically independent samples), barrier-destruction (Q and T, n=3 biologically independent samples) and transmigration (R and U, n=4 biologically independent samples) for A549/DTX cells after *GLUD1* knockdown and *SNAI1*overexpression. (V-W) Effects of *SNAI1* manipulation on GLUD1 binding to SNAIL sites (V) and VE-Cadherin protein levels (W, representative images from three independent experiments are shown.). (X) Protein level of GLUD1/SNAIL in nucleus and VE-Cadherin in whole cell lysate of primary tumor and circulation tumor cells. Representative images from three independent experiments are shown. (Y) Enhanced VE-Cadherin/GLUD1/SNAIL level in leptomeningeal metastasis-competent A549/DTX. Representative images from three independent experiments are shown. All data are represented as mean ± SEM. P values were calculated using two-tailed unpaired Student's t-test (A,B,E,F); one-way ANOVA followed by Dunnett's multiple-comparisons test (C,M,O,S,U,V); two-way ANOVA followed by Šídák multiple-comparisons test (N,T). Scale bars: J,L,P,R 1000 μm; H,K,Q, 20 μm. Source data are provided as a Source Data file. For immunoblots, samples within each panel were derived from the same experiment, with separate gels processed in parallel where applicable; detailed gel assignments are provided in the Methods.

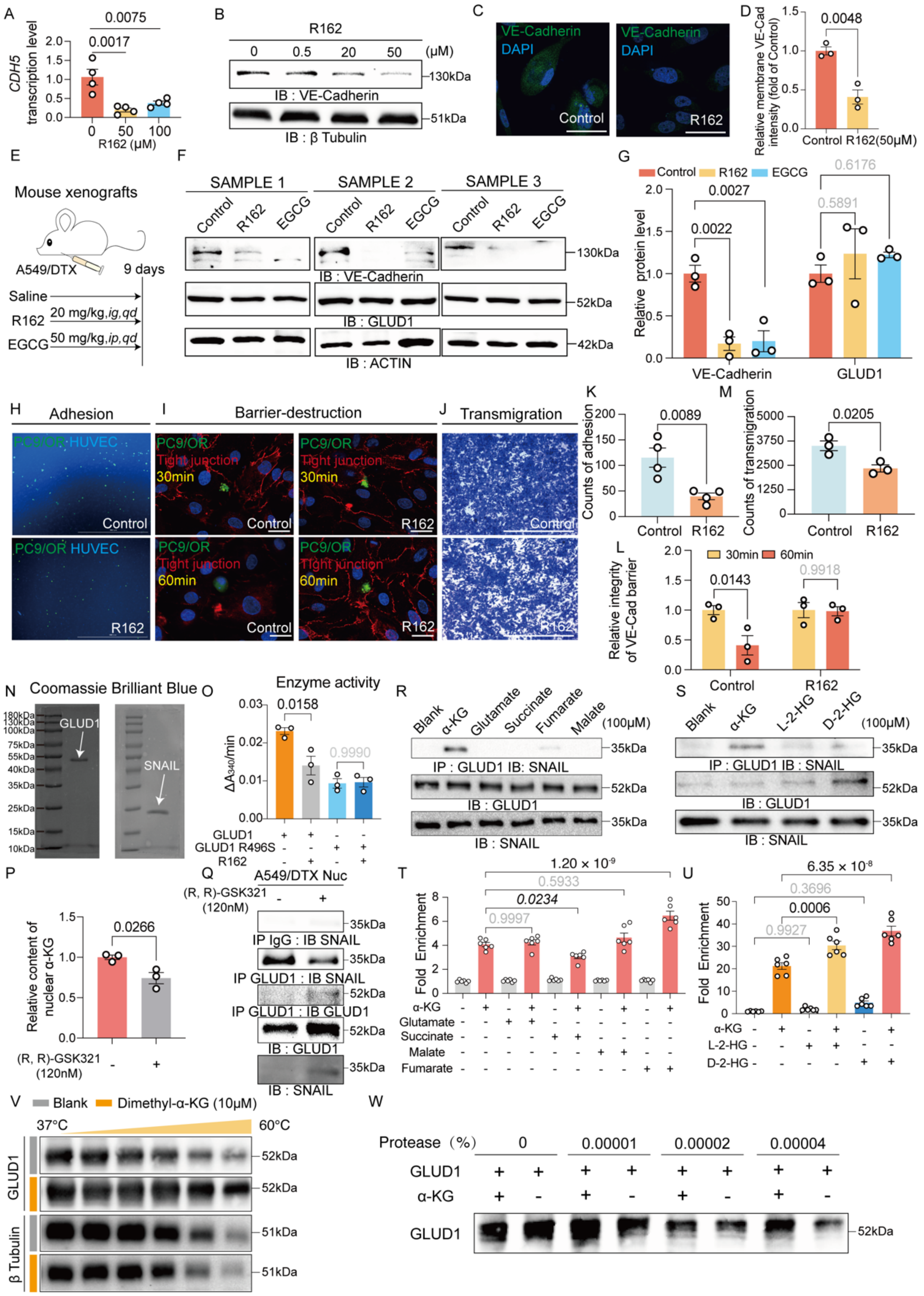

Supplementary Fig. 6 α-KG is vital for the formation of GLUD1-SNAIL complex. Related to Fig. 4. (A) Dose-dependent *CDH5* mRNA suppression by R162 (n=4 biologically independent samples). (B) VE-cadherin protein levels after R162 treatment (0, 0.5, 20 and 50 μM). Representative images from three independent experiments are shown. (C-D) Membrane VE-cadherin immunofluorescence after 50 μM R162 treatment (C, representative images from three independent experiments are shown) and quantification (D). Membrane-associated fluorescence

was quantified within manually defined regions using Fiji. (E-G) Experimental scheme (E), immunoblots (F, sample 1-3 represents sample from three independent mice) and quantification (G, n=3 mice per group) of VE-cadherin and GLUD1 in xenograft tumors treated with R162 (20 mg/kg, oral gavage, daily) or EGCG (50 mg/kg, intraperitoneally, daily). Protein levels were normalized to β-tubulin. (H-M) Representative immunofluorescence images (H-J) and quantification (K-M) of adhesion (H and K, n=4 biologically independent samples), barrier-destruction (I and L, n=3 biologically independent samples) and transmigration (J and M, n=3 biologically independent samples) for PC9/OR cells treated with DMSO or R162 (50 μM). (N) Coomassie staining of purified GLUD1 and SNAIL. Protein purification was independently performed and validated for each preparation; a representative image from more than three independent protein purification experiments is shown. (O) Enzymatic activity of wild-type or R496S loss-of-function GLUD1 in A549 cells treated with 50 μM R162, n=3 biologically independent samples. (P) Nuclear α-KG measured by LC–MS/MS in A549/DTX cells treated with DMSO or (R,R)-GSK321 (120 nM), n=3 biologically independent samples. (Q) Nuclear GLUD1/SNAIL complexes after (R,R)-GSK321 treatment (representative images from three independent experiments are shown). (R-S) Effects of α-KG and related metabolites on GLUD1/SNAIL complex formation in purified proteins (glutamate, succinate, fumarate and malate in O; L-2-HG and D-2-HG in P; 100 μM each; representative images from three independent experiments are shown). (T-U) Effects of these metabolites on GLUD1/SNAIL binding to the *CDH5* promoter in purified proteins, normalized to input, n=6 biologically independent samples. (V) Representative result of CETSA analysis of α-KG/GLUD1 interaction (10 μM dimethyl-α-KG vs DMSO). The experiment was independently repeated three times with similar results. (W) Representative result of DARTS analysis of α-KG-mediated protection of GLUD1 from subtilisin digestion (100 μM α-KG). The experiment was independently repeated three times with similar results. Data are represented as mean ± SEM. P values were calculated using one-way ANOVA followed by Dunnett's multiple-comparisons test (A,G); one-way ANOVA followed by Tukey's multiple-comparisons test (O,T,U); two-tailed unpaired Student's t-test (D,K,M,P); two way ANOVA followed by Šídák multiple-comparisons test (L). Scale bars: C,I, 20 μm; H,J, 1000 μm. Source data are provided as a Source Data file. For immunoblots, samples within each panel were derived from the same experiment, with separate gels processed in parallel where applicable; detailed gel assignments are provided in the Methods.

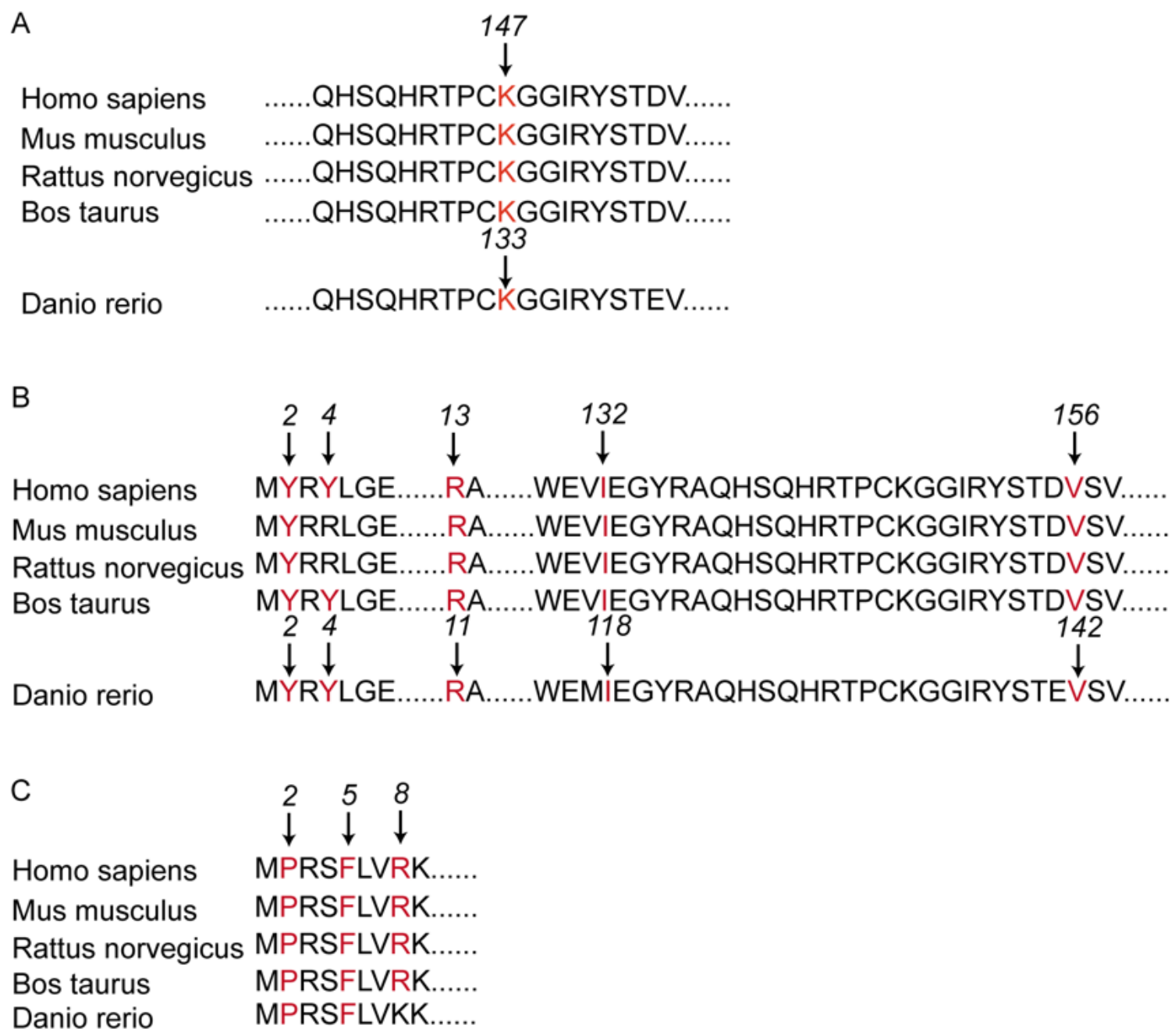

Supplementary Fig. 7 The key residues mediating α-KG/GLUD1/SNAIL interaction are conserved across species. Related to Fig. 5. (A) Cross-species conservation of the α-KG-binding residue K147 in GLUD1. (B-C) Cross-species conservation of GLUD1 (B) and SNAIL (C) residues contributing to the GLUD1-SNAIL interface.

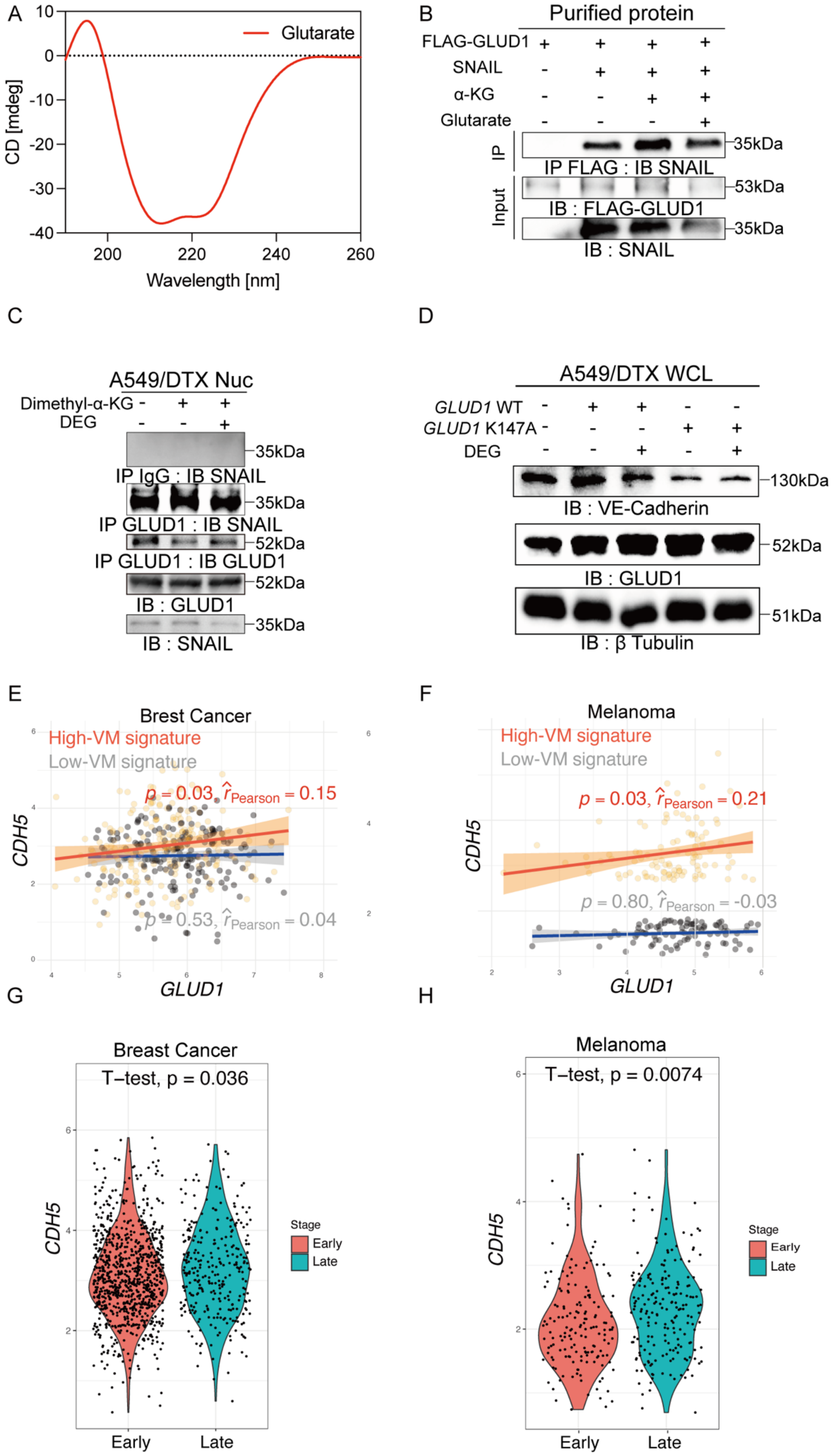

Supplementary Fig. 8 Glutarate competitively inhibits α-KG engagement and blocks ternary

complex formation. Related to Fig. 6. (A) Circular dichroism analysis of GLUD1 in the presence of 100 μM glutarate. A representative result from three independent experiments is shown. (B) Anti-FLAG immunoprecipitation demonstrating glutarate disruption of the α-KG/FLAG-GLUD1/SNAIL/ ternary complex, the concentration of α-KG and Glutarate is 100 μM (representative of three independent experiments). (C) Co-immunoprecipitation of endogenous GLUD1 complexes in A549/DTX cells treated with 10 μM dimethyl-α-KG or DEG (representative of three independent experiments). (D) Western blot showing VE-Cadherin expression in A549/DTX cells expressing wildtype or K147A GLUD1 with DEG treatment (representative of three independent experiments). (E-F) Correlation of GLUD1 and CDH5 expression in high- and low-VM signature breast cancer (E, high-VM subgroup included top 200 independent patients and low-VM subgroup included bottom 200 independent patients) and melanoma cohorts (F, high-VM subgroup included top 100 independent patients and low-VM subgroup included bottom 100 independent patients) from TCGA. (G-H) Association of CDH5 expression with clinical stage in breast cancer (G; n = 1,226 independent patients) and melanoma cohorts (H; n = 476 independent patients) from TCGA P values were calculated using two-sided Pearson correlation analysis (E,F); two-tailed unpaired Student's t-test (G,H). Source data are provided as a Source Data file. For immunoblots, samples within each panel were derived from the same experiment, with separate gels processed in parallel where applicable; detailed gel assignments are provided in the Methods.
