# Supplementary material for: α-KG conformationally stabilizes nuclear GLUD1-SNAIL complex to fuel trans-endothelial migration in metastasis
Source: Nat Commun. 2026 Aug 31;17:10377. doi: 10.1038/s41467-026-77149-0 (PMC13627656; doi:10.1038/s41467-026-77149-0)
Supplement: Supplementary file 2 [file 41467_2026_77149_MOESM2_ESM.pdf]

Description of Additional Supplementary Files

Supplementary Data 1: Reagents and tools used in this study.

Supplementary Data 2: Sequences of primers, siRNAs and expression constructs used in this study.

Supplementary Data 3: Clinicopathological information for the human NSCLC specimens.
