# Supplementary material for: α-KG conformationally stabilizes nuclear GLUD1-SNAIL complex to fuel trans-endothelial migration in metastasis
Source: Nat Commun. 2026 Aug 31;17:10377. doi: 10.1038/s41467-026-77149-0 (PMC13627656; doi:10.1038/s41467-026-77149-0)
Supplement: Supplementary file 3 [file 41467_2026_77149_MOESM6_ESM.pdf]

nature portfolio

Corresponding author(s): Jiali Liu, Fang Zhou, Guangji Wang

Last updated by author(s): August 14, 2026

Reporting Summary

Nature Portfolio wishes to improve the reproducibility of the work that we publish. This form provides structure for consistency and transparency in reporting. For further information on Nature Portfolio policies, see our Editorial Policies and the Editorial Policy Checklist.

Statistics

For all statistical analyses, confirm that the following items are present in the figure legend, table legend, main text, or Methods section.

n/a	Confirmed	
☒	☐	The exact sample size (*n*) for each experimental group/condition, given as a discrete number and unit of measurement
☐	☒	A statement on whether measurements were taken from distinct samples or whether the same sample was measured repeatedly
☐	☒	The statistical test(s) used AND whether they are one- or two-sided<br>*Only common tests should be described solely by name; describe more complex techniques in the Methods section.*
☒	☐	A description of all covariates tested
☐	☒	A description of any assumptions or corrections, such as tests of normality and adjustment for multiple comparisons
☐	☒	A full description of the statistical parameters including central tendency (e.g. means) or other basic estimates (e.g. regression coefficient) AND variation (e.g. standard deviation) or associated estimates of uncertainty (e.g. confidence intervals)
☐	☒	For null hypothesis testing, the test statistic (e.g. *F*, *t*, *r*) with confidence intervals, effect sizes, degrees of freedom and *P* value noted<br>*Give P values as exact values whenever suitable.*
☒	☐	For Bayesian analysis, information on the choice of priors and Markov chain Monte Carlo settings
☒	☐	For hierarchical and complex designs, identification of the appropriate level for tests and full reporting of outcomes
☒	☐	Estimates of effect sizes (e.g. Cohen's *d*, Pearson's *r*), indicating how they were calculated

Our web collection on statistics for biologists contains articles on many of the points above.

Software and code

Policy information about availability of computer code

Data collection	R v4.3.2 was used to retrieve data from the TCGA and GEO databases. Analyst TF 1.5.1 software was used for LC–MS/MS data acquisition. Gen5 v3.15 and FV31S-SW v2.3.1.163 was used for microscopy and Living Image v4.4 was used for in vivo imaging data acquisition.
Data analysis	R v4.3.2, Seurat v5.0.1 and DESeq2 v1.42.0 were used for bioinformatics analysis. GraphPad Prism v10.3.1 was used for statistical analysis and data visualization. Fiji/ImageJ 2.0.0 was used for image quantification. MaxQuant v1.6.14 was used for protein mass spectrometry data processing, and Analyst TF 1.5.1 was used for targeted LC-MS/MS quantification.

For manuscripts utilizing custom algorithms or software that are central to the research but not yet described in published literature, software must be made available to editors and reviewers. We strongly encourage code deposition in a community repository (e.g. GitHub). See the Nature Portfolio guidelines for submitting code & software for further information.

Data

Policy information about availability of data

All manuscripts must include a data availability statement. This statement should provide the following information, where applicable:

- Accession codes, unique identifiers, or web links for publicly available datasets
- A description of any restrictions on data availability
- For clinical datasets or third party data, please ensure that the statement adheres to our policy

The TCGA datasets analysed in this study are available through the UCSC Xena Browser (https://xenabrowser.net/). The publicly available lung cancer and

melanoma single-cell RNA-sequencing datasets are available from the Gene Expression Omnibus under accession codes GSE131907 (https://www.ncbi.nlm.nih.gov/geo/query/acc.cgi?acc=GSE131907) and GSE215121 (https://www.ncbi.nlm.nih.gov/geo/query/acc.cgi?acc=GSE215121), respectively. The IP–mass spectrometry data related to Supplementary Fig. 4G generated in this study have been deposited in the ProteomeXchange Consortium via the iProX partner repository under accession code PXD075031 (https://www.iprox.cn/page/project.html?id=IPX0015899000). The DNA pull-down–coupled mass spectrometry data related to Fig. 3G have been deposited in the ProteomeXchange Consortium via iProX under accession code PXD075033 (https://www.iprox.cn/page/project.html?id=IPX0015902000). The targeted LC–MS/MS data for nuclear α-ketoglutarate quantification related to Fig. 4O have been deposited in MetaboLights under accession code MTBLS15115 (https://www.ebi.ac.uk/metabolights/MTBLS15115).
No custom code was developed for this study, as stated in the Code Availability section.
No additional data required for reanalysis are available only upon request.

Research involving human participants, their data, or biological material

Policy information about studies with human participants or human data. See also policy information about sex, gender (identity/presentation), and sexual orientation and race, ethnicity and racism.

Reporting on sex and gender: This study analysed de-identified publicly available human datasets and de-identified archival human NSCLC specimens. No sex-based filtering was applied, and both female and male participants were included where applicable. As the study was not designed or powered to assess sex-based differences, sex was not included as a covariate in the analyses.

Reporting on race, ethnicity, or other socially relevant groupings: Race, ethnicity and other socially relevant groupings were not analysed. These demographic variables were unavailable or incompletely reported in the de-identified public datasets and archival specimen cohort.

Population characteristics: In the TCGA datasets, the lung adenocarcinoma cohort comprised 357 females and 301 males; the melanoma cohort comprised 180 females and 296 males; the thyroid carcinoma cohort comprised 423 females and 157 males; the prostate cancer cohort, which inherently included only male patients, comprised 572 males; and the breast cancer cohort, which predominantly included female patients, comprised 1,241 females and 13 males. In the GEO single-cell datasets, the lung adenocarcinoma cohort included 17 females and 27 males, and the melanoma cohort included 3 females and 5 males. The in-house cohort related to Fig 1D-1E included 11 females and 8 male.

Recruitment: No participants were prospectively recruited specifically for this study. The in-house cohort consisted of archival specimens collected from participants who had provided written informed consent for research participation.

Ethics oversight: The use of de-identified archival human NSCLC specimens was approved by the Research Ethics Committee of the First Affiliated Hospital of Nanjing Medical University (approval no. 2019-SR-450). Written informed consent to participate in the research was obtained from all participants. Separate consent for the publication or public sharing of individual-level participant data was not obtained; therefore, only aggregated demographic information is reported in supplementary data 3.

Note that full information on the approval of the study protocol must also be provided in the manuscript.

Field-specific reporting

Please select the one below that is the best fit for your research. If you are not sure, read the appropriate sections before making your selection.

☒ Life sciences ☐ Behavioural & social sciences ☐ Ecological, evolutionary & environmental sciences

For a reference copy of the document with all sections, see nature.com/documents/nr-reporting-summary-flat.pdf

Life sciences study design

All studies must disclose on these points even when the disclosure is negative.

Sample size: No statistical methods were used to predetermine sample size; instead, sample sizes were determined based on the frequently used number of experimental replicates found in the existing literature. For each experiment, we aimed to obtain a sample size sufficient for statistical analysis, with the exact number specified in the figure legend.

Data exclusions: No data were excluded from the analyses.

Replication: Biological replicates for each experiment are specified in the figure legends, with all experiments comprising a minimum of two or three replicates. In vivo experiments were conducted with an ample number of mice to guarantee statistical power in detecting variances between control and treated groups.

Randomization: Animals were randomly assigned to treatment groups. No randomization was applied to in vitro experiments.

Blinding: Data were collected prior to analysis, and investigators performing quantitative analysis were blinded to group allocation. Image quantification was conducted using automated software with identical parameters applied across all groups.

Reporting for specific materials, systems and methods

We require information from authors about some types of materials, experimental systems and methods used in many studies. Here, indicate whether each material, system or method listed is relevant to your study. If you are not sure if a list item applies to your research, read the appropriate section before selecting a response.

Materials & experimental systems

n/a	Involved in the study
☐	☒ Antibodies
☐	☒ Eukaryotic cell lines
☒	☐ Palaeontology and archaeology
☐	☒ Animals and other organisms
☒	☐ Clinical data
☒	☐ Dual use research of concern
☒	☐ Plants

Methods

n/a	Involved in the study
☒	☐ ChIP-seq
☒	☐ Flow cytometry
☒	☐ MRI-based neuroimaging

Antibodies

Antibodies used

Rabbit polyclonal anti-VE-Cadherin is obtained from Abcam Cat# ab33168 1:1000 for Western blot 1:300 for Immunofluorescence Staining
Rabbit polyclonal anti-Lamin B1 is obtained from Abcam Cat# ab16048 1:5000
Rabbit Monoclonal Sodium/Potassium ATPase alpha 1 is obtained from Abcam Cat# ab308329 1:1000
Rabbit monoclonal anti-DDDDK tag is obtained from Abcam Cat# ab205606 1:10000
Mouse monoclonal anti-beta Actin is obtained from Abcam Cat# ab8226 1:2000
Goat anti-rabbit IgG H&L (HRP) is obtained from Abcam Cat# ab6721 1:10000
Goat anti-mouse IgG H&L (HRP) is obtained from Abcam Cat# ab6789 1:10000
Rabbit polyclonal anti-GLUD1 is obtained from Proteintech Cat# 14299-1-AP 1:1000
Mouse Monoclonal anti-GLUD1 is obtained from Proteintech Cat# 67026-1-IG 1:300
Rabbit polyclonal anti-SNAIL is obtained from Proteintech Cat# 13099-1-AP 1:1000 for Western blot 1:200 for Immunofluorescence Staining
Rabbit polyclonal anti-FAK is obtained from Proteintech Cat# 12636-1-AP 1:1000
Rabbit polyclonal anti-EPHA2 is obtained from Proteintech Cat# 25749-1-AP 1:1000
Rabbit Monoclonal anti-Phospho-FAK (Tyr397) is obtained from Proteintech Cat# 83933-1-RR 1:1000
Rabbit Monoclonal anti-Phospho-EPHA2 (Tyr588) is obtained from Proteintech Cat# 30263-1-AP 1:1000
Rabbit polyclonal anti-SOX2 is obtained from Proteintech Cat# 11064-1-AP 1:500
Rabbit polyclonal anti-SLUG is obtained from Proteintech Cat# 12129-1-AP 1:500
Rabbit polyclonal anti-ZEB1 is obtained from Proteintech Cat# 21544-1-AP 1:500
Beta Tubulin Recombinant antibody is obtained from Proteintech Cat# 80713-1-RR 1:1000
Rabbit polyclonal anti-Importin 8 is obtained from Thermofisher Cat# PA1-41599 1:1000
Rabbit polyclonal anti-GFP is obtained from Thermofisher Cat# PA1-980A 1:1000
Rabbit polyclonal anti-Hsp60 is obtained from MedChemExpress Cat# HY-P80184 1:200

Validation

Primary antibodies were validated by the manufacturers for the indicated applications. For key antibodies, specificity was further supported by knockdown or overexpression controls and detection at the expected molecular weight, where applicable.

Eukaryotic cell lines

Policy information about cell lines and Sex and Gender in Research

Cell line source(s)

Human A549/DTX cells are constructed by this paper
Human PC9/OR cells are constructed by this paper
Human PC-9 cells are obtained from CellBank Cat# SCSP-5085
Human A549 cells are obtained from CellBank Cat# SCSP-503
Human NCI-H1975 cells are obtained from CellBank Cat# SCSP-597
Human NCI-H460 cells are obtained from CellBank Cat# SCSP-584
Primary HUVEC are obtained from ZhongQiaoXinZhou Co., Ltd., Cat# DFSC-EC-01

Authentication

Commercially available cell lines were authenticated by the Cell Bank of the Chinese Academy of Sciences. The efficiency of gene knockdown or overexpression in the engineered cell lines was confirmed by western blotting.

Mycoplasma contamination

All cell lines were routinely tested and confirmed to be negative for mycoplasma contamination.

Commonly misidentified lines
(See ICLAC register)

No commonly misidentified cell lines were used in the study.

Animals and other research organisms

Policy information about studies involving animals; ARRIVE guidelines recommended for reporting animal research, and Sex and Gender in Research

Laboratory animals	Balb/c nude mice(Beijing Vital River Laboratory Animal Technology), Danio rerio zebra fish(Hunter Biotech)
Wild animals	No wild animals were used in this research.
Reporting on sex	For the zebrafish xenograft experiments, biological sex cannot be reliably determined; therefore, sex was not considered as a biological variable in these experiments. For the Balb/c nude mouse experiments, female mice were used to minimize fighting-related stress and injuries during group housing, which could otherwise affect tumor growth and therapeutic responses.
Field-collected samples	This study did not involve field-collected samples.
Ethics oversight	All animal procedures were approved by the Institutional Animal Care and Use Committee (IACUC) of China Pharmaceutical University (Approval No. SYXK2021-0010) and conducted in accordance with the National Research Council's Guide for the Care and Use of Laboratory Animals.

Note that full information on the approval of the study protocol must also be provided in the manuscript.

Plants

Seed stocks	not applicable
Novel plant genotypes	not applicable
Authentication	not applicable
