# Supplementary material for: α-KG conformationally stabilizes nuclear GLUD1-SNAIL complex to fuel trans-endothelial migration in metastasis
Source: Nat Commun. 2026 Aug 31;17:10377. doi: 10.1038/s41467-026-77149-0 (PMC13627656; doi:10.1038/s41467-026-77149-0)
Supplement: Supplementary file 4 [file 41467_2026_77149_MOESM7_ESM.pdf]

nature portfolio

Peer Review File

α-KG conformationally stabilizes nuclear GLUD1-SNAIL complex to fuel trans-endothelial migration in metastasis

Corresponding Author: Professor Jiali Liu

This file contains all reviewer reports in order by version, followed by all author rebuttals in order by version.
Parts of this Peer Review File have been redacted as indicated to maintain the confidentiality of unpublished data.

Version 0:

Reviewer comments:

Reviewer #1

(Remarks to the Author)
In this manuscript, Cui et al., report their findings of GLUD1 in trans-endothelial cell migration (TEM) of the metastatic cascade. They show that alpha-keto glutamate as a metabolite of glutamate, which is concerted by GLUD1, acts as a allosteric stabilizer for GLUD1. in the nucleus, the formation of α-KG/GLUD1/SNAIL ternary complex that promotes metastasis via the VE-Cadherin.

Comments:

1. Cancer metastasis is a complex and multistep process that requires metastatic cancer cells to at least transmigration the vessel wall twice, i.e., intravasation and extravasation. The intravsation often occurs in the primary tumor microenvironment whereas the extravasation happens in the circulation. Thus, intravasation and extravasation are two independent processes and occur in different tissues and locations. Thus, the transmigration of out-in and in-out through the vessel wall are differentially regulation in relation to the presence of primary tumors, blood flow and heterogeneity of micro vessels. In their in vitro and in vivo assays, the authors have not taken these variation into account to mechanistically address the real issue of TEM. Their both in vitro and in vivo assays are based on in the presence of primary tumors. Therefore, despite their claims, they have not addressed the issue of extravasation of tumor cells. This should be done in the absence of primary tumors. A experimental metastasis model should be employed for further investigations.

2. This reviewer is surprised with their experimental designs. They started their discovery using the vasculogenic mimicry (VM) that represents vasculature-like structures formed by tumor cells rather than vascular endothelial cells. All human sample studies and the so called unbiased approaches are based on VM-mediated metastasis. This issue is still highly controversial. The key mechanistic issue of VM in cancer metastasis remains unresolved. How could VM be connected to the blood stream ? Would these tumor cell-formed channels be connected to blood vessels ? If so, why would tumor cells have to transmigrate through the vessel wall. The tumor cells could through the VM lumen to travel into the blood stream. If not, what VM has anything to do with TEM. This reviewer has difficulties to understand the rationale for dragging VM into the experimental design. Particularly, this is the initial starting point of the whole project. Designing a TEM-related project should have a novel rationale, but not just according to what is known in the literature's. Having said this, this project also lacks conceptual novelties although they have devoted great efforts to molecular characterization.

3. This reviewer also has another important question that remains unresolved. VE-cadherin could acts has an adhesion molecule to tether cancer cells. In this regard, VE-cadherin acts as pro-metastatic adhesion molecule. TEM occurs through opening of the inter-endothelial cell junctions which are tightly sealed by VE-cadhrin and other adhesion molecules. to break up this tight junction, they need to destroy or downregulate VE-cadherin. These authors have not really deal with the key mechanism of TEM. How do tumor cells go through the endothelial cell tight junctions ? Unfortunately, no information has been provided to address this issue.

4. The zebrafish model is an inappropriate to investigate metastasis because the primary tumor cancer microenvironment is missing. Also, it is inappropriate to study extraversion in this model.

5. They authors completely ignore the role of tumor stroll cellular and molecular components in cancer metastasis in thier studies. TAMS and CAFs all play important roles in interaction with tumor cells and blood vessels.

6. For extravasation, they authors have not dead with the issue of specific extravasation sites in their preferred tissues/organs.

In summary, the authors findings are too preliminary and major flaws exist in the current version of the manuscript. Particularly, conceptual and rational misconceptions make this reviewer not enthusiastic.

Reviewer #2

(Remarks to the Author)
In this manuscript, Cui et al,. found the relationship between GLUD1 and the tumor trans-endothelial migration (TEM) and also did experiments try to figure out the molecular mechanism for explaining how the GLUD1 promotes TEM. This is an interesting finding, cause the non-canonical function of enzyme still beyond the boundaries of human understanding. However, I still have some concerns before its acceptance.

1.The absolute cellular α-KG concentration (not the relative concentration) is very important and requires thorough investigation. Previous studies have suggested that the mammalian cellular α-KG concentration is approximately 0.1–5 mM (PMID: 27189937; PMID: 19935646). Therefore, the absolute cellular α-KG concentrations of the cell lines used in this manuscript would be valuable. Experiments to determine the dimerization abilities of GLUD1-SNAIL under conditions with different α-KG concentrations (either in cellular or biochemical experiments) are required. The effective α-KG concentration should be relevant to the cellular or physiological concentration.

2. The purified GLUD1 and SNAIL proteins used in biochemical analyses should be presented with Coomassie brilliant blue-stained SDS-PAGE images.

3. The authors state that α-KG induces allosteric regulation of the GLUD1-SNAIL heterodimer; however, only the AI-predicted PDB models are insufficient. The authors should perform experiments to determine the complex structure of α-KG-GLUD1-SNAIL and analyze the conformational changes compared with those of the GLUD1-SNAIL complex or GLUD1 alone. Alternatively, they should revise their statement.

4. The nucleus allows molecules (<10 kDa) to freely enter and exit. The authors detected the α-KG concentration in the nucleus; how were these experiments designed and conducted? The authors should add the experimental details in the Methods section.

5. The authors should analyze the cross-species conservation of the key residues in Fig. 5, and the animal species used in this manuscript should be included.

6. K147 of GLUD1 forms a hydrogen bond with the carboxyl group of α-KG. Can dimethyl-α-KG, which lacks a carboxyl group, also form a hydrogen bond with K147? In general, this structural change would disrupt the original interaction. Since a lower concentration of dimethyl-α-KG induced significant VE-Cad expression, the authors should add experiments to determine the Kd value of dimethyl-α-KG and discuss the potential underlying causes.

7. Diethyl glutarate can also bind to GLUD1 and exhibit competitive inhibitory activity. The structural differences between α-KG, dimethyl-α-KG, and diethyl glutarate should be discussed. It is notable that these three structurally similar chemicals exhibit significantly different results.

8. All α-KG concentrations used in the experiments should be added to the figure captions.

9. Can α-KG-like metabolites (e.g., glutamate, succinate, D/L-2HG, malate, fumarate) play a similar role or a competitive inhibitory role?

Minor:

1. IDH1, a NADP-dependent reversible enzyme, is one of the isocitrate dehydrogenases (IDHs). Other IDHs, such as IDH2 (also NADP-dependent) and especially IDH3 (NAD-dependent), may be key α-KG-generating enzymes because of the high cellular NAD/NADP ratio and the fact that NAD is the commonly used H+ carrier for oxidative phosphorylation. The reasons for the authors' choice of IDH1 should be presented.

2. Which group do the gray dots in Fig. 5B belong to?

3. The marked colors in Fig. 5D are incorrect.

4. The predicted PDB models should be uploaded for free access.

Reviewer #3

(Remarks to the Author)
In this study by Cui et al., the authors uncover a unique non-canonical role of nuclear GLUD1 in tumor metastasis. The authors demonstrated that GLUD1 is transported to the nucleus by the transporter importin-8, where it binds to SNAIL,

inducing metastasis-promoting genes and inducing tumor metastasis. The authors showed that the interaction between SNAIL and GLUD1 is enhanced by GLUD1 product alpha-ketoglutarate (a-KG), which allosterically binds to GLUD1 through K147 and stabilizes the GLUD1-SNAIL complex. Overall, the discovery of an enzyme-independent role for GLUD1 as a scaffold protein that promotes transcriptional regulation is intriguing, and the data presented generally support this finding. Several issues identified could be addressed through additional experiments and clarification to provide concrete mechanistic insight into the non-classical role of GLUD1 in human cancer.

1. While the authors are commended for validating the role of GLUD1 using both RNAi- and overexpression-based approaches in zebrafish and murine models, the knockdown experiments rely on single siRNAs (e.g., siGLUD1, siImportin-8). In addition, much of the mechanistic work is conducted in a single cell line (A549) and its derivative. It would strengthen the study to validate the key findings using multiple siRNAs and additional cancer cell lines.

2. The authors modulated potential effector SNAIL and downstream target VE-cadherin in the GLUD1 overexpression setting using siRNA. It is worth determining whether the rescue effects of VE-cadherin or SNAIL are specific or more pronounced in cells overexpressing GLUD1. Can VE-cadherin SNAIL knockdown alter TEM regardless of GLUD1 overexpression? In addition, it would be useful to determine whether the effects of VE-cadherin or SNAIL overexpression on TEM are diminished or overshadowed when cells are lacking GLUD1.

3. The manuscript compares nuclear and cytosolic levels of GLUD1 (Figure 3), but GLUD1 is known to reside predominantly in the mitochondria. The authors should clarify what fraction of total cellular GLUD1 is transported to the nucleus when the mitochondrial, cytosolic, and nuclear pools are considered.

4. On a related note, it is worth knowing whether nuclear transport of GLUD1 is more prominent in metastatic cancer cells than in cells with low TEM potential. Or whether nuclear GLUD1 is higher in circulating tumor cells that have undergone intravasation or extravasation compared to primary tumors.

5. Does GLUD1 specifically and only bind to SNAIL and no other transcription factor related to EMT? It would be ideal to have experimental controls. In addition, Figures 3m, 3r, 4k, 4o, and 5g, IgG control and GLUD1 immunoprecipitation samples should be loaded in parallel for comparison. If different exposure has been used, it is difficult to compare.

6. In Figure 4, the R162 treatment needs an appropriate control. It is worth demonstrating whether R162 worked in these cells, possibly by assessing GLUD1 activity or intracellular a-KG levels. In addition, the authors used 50 microM, which is relatively high and may be toxic to cells, potentially reducing global protein levels. Additional controls could be included in the ChIP or biophysical assays to determine whether a-KG, but not other intermediate metabolites, specifically promotes the SNAIL-GLUD1 complex formation.

7. How did the authors select GLUD1 from the primary bioinformatics screening? It seems some of the representative positive or negative correlative factors are selectively shown in Figure 1c.

Reviewer #4

(Remarks to the Author)
This study uncovers a previously unrecognized non-canonical function of glutamate dehydrogenase 1 (GLUD1) in tumor transendothelial migration (TEM). The authors demonstrate that α-KG binds to a non-enzymatic site (K147) on GLUD1, inducing conformational changes that stabilize its interaction with the transcription factor SNAIL in the nucleus. This α-KG/GLUD1/SNAIL ternary complex activates VE-cadherin transcription, thereby promoting tumor cell TEM and metastasis. The findings reveal a novel "allosteric stabilization" mechanism by which a primary metabolite directly modulates transcriptional complex assembly. However, several key mechanistic and translational aspects require additional clarification and experimental reinforcement.
Major Comments:
1. In Fig 1A, the authors utilize the MetMap database to distinguish strongly metastatic (lung cancer, melanoma) from weakly metastatic tumors (thyroid cancer, prostate cancer) and observe that VM markers are enriched in aggressive tumors, leading to the conclusion that VM plays an important role in metastasis. Given the substantial inherent differences in gene expression and cellular origin across distinct tumor types, baseline VM expression may vary independently of metastatic capacity. To strengthen the rigor of this conclusion, the authors should perform a correlation analysis between VM marker expression and the incidence of metastasis from primary tumors within the same tumor type. This additional data will further validate the functional relevance of VM in metastasis.
2. In Figs. 1B and 1C, the authors identified 102 genes through the intersection of three cohorts (Fig. 1B) and further narrowed down the candidates to 10 genes by correlating with overall survival (OS) and failure-free survival (FFS). Among these, GLUD1 emerged as the lead candidate and the only endogenous metabolic enzyme, prompting the authors to focus on GLUD1. The authors should analyze whether any of these 10 candidate genes have been previously reported to be implicated in VM regulation or TEM. Such an analysis would serve as valuable validation of the screening strategy, reinforcing the biological relevance of the identified gene set and lending credence to the subsequent focus on GLUD1. Additionally, as a critical starting point for the study’s data analysis, the authors need to supplement correlation data between GLUD1 and VM expression across the tumor types in the TCGA database, rather than relying solely on the IHC staining from 19 lung cancer specimens.
3. The formation of metastatic lesions is a complex process influenced by multiple factors beyond TEM. Therefore, in the

murine metastasis model, quantifying the number of tumor cells adjacent to blood vessels in target organs at early time points (e.g., 24 or 48 hours) post-intravenous injection of tumor cells will more accurately reflect TEM efficiency. Furthermore, after metastatic lesion formation, counting the number of metastatic lesions (in addition to measuring lesion area) is essential, as it partially reflects the number of colonized cells in target organs and thus indirectly indicates TEM levels.

4. In Fig. 2, the authors examined three key mediators of TEM (VE-Cadherin, EPHA2, and FAK) in GLUD1-overexpressing or knockdown cells and found that EPHA2 and FAK protein levels were unaffected by GLUD1. Consequently, they excluded EPHA2 and FAK and identified VE-Cadherin as the sole downstream effector of GLUD1. This logical deduction is insufficiently rigorous: EPHA2 and FAK exert their biological functions through extensive phosphorylation-dependent activation. Thus, unchanged protein expression levels do not rule out potential regulation of their activity. The authors must supplement experiments to detect the phosphorylation levels of key functional sites in EPHA2 and FAK in GLUD1-overexpressing or knockdown cells.

5. In Fig. 3E, the distribution of GLUD1 in the cytoplasm needs to be presented. Quantitative analysis of the nuclear-to-cytoplasmic ratio will provide more robust support for the results. Additionally, GLUD1 expression in whole-cell lysates appears to be significantly reduced following siImportin-8 treatment, and the authors should provide a detailed explanation for this observation. In Fig. 3F, including A549 wild-type (WT) cells as a control will better facilitate the evaluation of VE-Cadherin expression upon GLUD1-NLS deletion.

6. The ChIP assays presented in Fig. 3I and Supplementary Fig. 4F lack IgG negative controls, which are essential for assessing the specific binding intensity of GLUD1 at each SNAIL binding site. For Supplementary Fig. 4F, the ChIP results for GLUD1 at each site must be normalized to its respective input before direct comparison, as primer sets targeting distinct regions inherently exhibit variable amplification efficiencies. Furthermore, the precise location of each binding site on the VE-Cadherin promoter and its motif sequence should be provided. The same issue applies to Supplementary Fig. 4I.

7. The dual luciferase reporter assays in Fig 3J lack a vector control to assess the baseline transcriptional activity of the VE-Cadherin promoter. Additionally, including data on the transcriptional activity of promoter-WT and promoter-MUT in both GLUD1-overexpressing and control cells will help evaluate the necessity of the SNAIL binding site for GLUD1-mediated VE-Cadherin transcription activation.

8. In Fig. 3L, the Co-IP assay results appear to indicate that the GLUD1-SNAIL interaction is not restricted to the nucleus but also occurs in the cytoplasm. The apparent absence of signal in the middle region of the cytoplasmic fraction blot is likely an experimental artifact—potentially arising from band distortion, uneven protein loading, or suboptimal exposure conditions. The authors are advised to carefully re-examine their original raw data to confirm the authenticity of the cytoplasmic GLUD1-SNAIL interaction and rule out technical artifacts.

9. While the authors demonstrated the interaction between GLUD1 and SNAIL in Fig. 3, they did not confirm whether this binding is required for SNAIL's binding to the VE-Cadherin promoter. The authors need to supplement ChIP data to show SNAIL binding to the VE-Cadherin promoter under GLUD1 overexpression or knockdown conditions, as well as data on SNAIL-mediated regulation of VE-Cadherin promoter activity.

10. In Fig. 3R, the authors state that the interaction between GLUD1 and SNAIL was markedly enriched in metastatic tumors compared to contralateral normal lung tissues. However, the data do not show striking differences in the expression levels of GLUD1 or SNAIL between metastatic tumors and normal lung tissues. The authors should explain why the GLUD1-SNAIL interaction is specifically enhanced in metastatic tumors despite similar protein expression levels.

11. In Fig 4A-N, the authors observe that the GLUD1 enzymatic inhibitor R162 attenuates the GLUD1-SNAIL interaction and TEM phenotypes. From these findings, they propose that "the reduction of nuclear α-KG resulted by R162 is involved in the regulation of complex assembly." However, this logical inference lacks sufficient experimental substantiation and overlooks alternative mechanisms. Critically, R162 binding to GLUD1 may directly disrupt the GLUD1-SNAIL interaction by altering GLUD1's conformation or interfering with their binding interface, independent of its effect on α-KG production. The authors must rule out these alternative possibilities. Specifically, they need to demonstrate that R162 binding to GLUD1 does not affect GLUD1's conformation or the GLUD1-SNAIL binding interface. Two complementary experimental strategies would be effective: (1) constructing a catalytically inactive GLUD1 mutant (via point mutation at the enzymatic active site) and repeating the key experiments to determine if R162 still attenuates the GLUD1-SNAIL interaction in the absence of enzymatic activity; or (2) performing in vitro pulldown assays with purified recombinant GLUD1 and SNAIL proteins to confirm that R162 does not directly interfere with their binding. Additionally, the authors need to provide quantitative data on intracellular α-KG levels following R162 treatment.

12. In Figs. 4K-N, although the authors demonstrated that α-KG can directly enhance GLUD1-SNAIL binding, there remains a logical gap in this reasoning. As a key intermediate metabolite, α-KG regulates multiple metabolic and downstream signaling pathways. Thus, the ability of α-KG to rescue the R162-induced reduction in GLUD1-SNAIL binding does not directly confirm that α-KG enhances their interaction directly. The authors should supplement experiments to show that inhibitors of enzymes directly responsible for α-KG metabolism do not affect this process, or at least provide a detailed discussion addressing this caveat.

13. While the authors demonstrated that the α-KG-GLUD1-SNAIL ternary complex promotes SNAIL binding to the VE-Cadherin promoter and enhances its transcription, the underlying mechanism remains unclear. Does the complex enhance SNAIL's DNA-binding affinity, protect SNAIL from degradation, or exert other effects? The authors should propose a plausible mechanism.

14. Glutarate mediates competitive inhibition of α-ketoglutarate (α-KG)-dependent metabolic enzymes via structural mimicry of α-KG: it occupies the enzyme's active site, thereby precluding α-KG binding and perturbing the catalytic cycle. Canonically, α-KG undergoes oxidative decarboxylation during enzymatic catalysis, providing energy and functional chemical groups essential for substrate modifications such as hydroxylation and demethylation. In contrast, glutarate lacks the critical α-keto group, rendering it unable to initiate oxidative decarboxylation. This deficiency leads to arrest of the enzyme's catalytic cycle and results in irreversible "non-productive binding-mediated inhibition"—a hallmark of its canonical inhibitory mechanism. However, the binding of α-KG to GLUD1, as delineated in this study, is distinct in that it does not entail oxidative decarboxylation. Thus, the well-established competitive inhibition paradigm for glutarate cannot be directly

extrapolated to its interaction with GLUD1. In fact, Fig. 5 shows that α-KG binding induces allosteric conformational changes in GLUD1. As a structural analog of α-KG, glutarate would theoretically be expected to exert a similar effect. The authors must supplement experiments to clarify why glutarate binding to GLUD1 fails to induce the same α-KG-like allosteric conformational changes that promote GLUD1-SNAIL complex formation. Without this clarification, it is reasonable to hypothesize that the anti-metastatic effects of glutarate may be mediated by its inhibition of other α-KG-dependent metabolic enzymes rather than through direct interference with the α-KG/GLUD1/SNAIL axis.
15. While diethyl glutarate (DEG) exhibits promising anti-metastatic efficacy, its long-term toxicity, metabolic stability, and tissue distribution have not been evaluated. The authors should temper their claims regarding its therapeutic potential or include preliminary toxicity data (e.g., changes in body weight, organ histology) from the murine studies.
16. The authors identified GLUD1 as a key mediator of TEM through integrated analysis across multiple tumor types (lung cancer, melanoma, thyroid cancer and prostate cancer). However, subsequent phenotypic and mechanistic experiments focused solely on lung cancer. Thus, it remains unclear whether the α-KG/GLUD1/SNAIL axis operates similarly in other tumor types. The authors should either include preliminary validation data in additional cancer types (e.g., melanoma, prostate cancer) or explicitly discuss this limitation and propose future research directions.
Minor Comments:
1.In Supplementary Fig. 5E, the image of the control group at 60 minutes is inconsistent with its statistical result. The authors should carefully verify this data. Additionally, these results (along with those in Fig. 4F) are not described or discussed in the manuscript.
2. Many experimental details are missing from the Figure legends, making it difficult to follow the methodology, particularly for the ChIP and dual luciferase reporter assays.
3. Lines 237–238 contain an erroneous description: "R162 also elevated tumor cell adhesion, barrier-destruction and transmigration across endothelial cells." Based on the study's findings, R162 should inhibit TEM-related processes, and this statement should be corrected accordingly.
4. Several linguistic expressions throughout the manuscript require refinement to enhance precision, clarity, and conformity with scientific writing conventions.

Version 2:

Reviewer comments:

Reviewer #1

(Remarks to the Author)
The authors have performed additional experiments to address my previous concerns. In general, the revised manuscript is improved. However, some of my previous concerns related to distinct steps of cancer metastasis remain.

1. The authors performed new experiments using a zebrafish metastasis model. There are some issues about the zebrafish model. a) This model lacks a truly primary tumor and a clinically relevant tumor microenvironment; b) Injection of cancer cells into the yolk sac of developing embryos does not really allow the authors to study the process of intravasation. All these processes occur through passive processed and not related to the invasiveness of cancer cells. Usually, cancer cells are implanted into the intervitelline space that allows cancer cell invasion and intravasate. Also, some excellent and important publications in zebrafish metastasis have not been cited.

2. The authors explained that VM was used as a marker to predict metastasis but not a structure for metastasis. However, the mechanistic link of this marker to invasiveness of cancer cells in to the circulation are hard-to-understand. Perhaps, the authors should clearly indicate that VM has been used as morphological markers and the mechanistic link is missing.

My purpose is to help the authors to improve their work and for readers to understand their findings with logic rationales. If the authors could in the manuscript text further clarify these issues, this reviewer is happy to support publication of this interesting work.

Reviewer #2

(Remarks to the Author)
Overall, the author has addressed most of my concerns, thanks. However, several major concerns still require serious attention and further resolution.

For Major Comment 1, the author stated that cellular α-KG concentrations in the tested cell lines were measured to range from 50 to 500 μM, and these data should be explicitly presented. In addition, IP data for the critical control group (0 μM α-KG treatment) and other concentration groups (10, 1000, and 5000 μM) should be supplemented in Figure A5. Furthermore, it remains unclear whether endogenous α-KG was eliminated prior to the IP experiments. If endogenous α-KG was not removed, its potential interference and influence on the experimental results should be thoroughly discussed.

For Major Comment 4, I appreciate the author's detailed response. Nevertheless, I am somewhat surprised that the nuclear α-KG concentration remains consistent after these complex experimental procedures. Consistent with the suggestion for Major Comment 1, the author should quantify the absolute nuclear α-KG concentration rather than only analyzing its relative content. This quantification is essential, as it allows readers to directly evaluate the assembly of the ternary complex induced by different α-KG concentrations.

For Major Comment 5, sequence alignment analyses across different species are missing for the key residues presented in Figures 5f and 5j.

For Major Comments 6 and 7, dimethyl-α-KG and diethyl glutarate were used at a concentration of 10 μM in this study. The author claimed that these two compounds can be metabolized into α-KG and glutarate, respectively. However, it is critical to verify whether 10 μM exogenous α-KG or glutarate addition is sufficient to induce significant activation or inhibition of the ternary complex. Moreover, it is unclear whether cells can actively uptake these two compounds, which may lead to a substantial discrepancy between intracellular and extracellular compound concentrations. Additionally, the removal of endogenous α-KG during the IP experiments needs to be confirmed.

For Major Comment 9, no data regarding D/L2HG is provided in Figure A10.

For additional suggestions, all newly acquired experimental data should be appropriately displayed in either the main figures or supplementary materials of the manuscript.

Reviewer #3

(Remarks to the Author)
The authors have addressed most of the concerns raised during the initial review. However, the new data generated and presented in the response to the review do not appear to have been incorporated into the revised manuscript; these data should be included.

Reviewer #4

(Remarks to the Author)
The authors have fully addressed all concerns raised in my initial review by presenting rigorous, well-designed experimental data, comprehensive analyses, and targeted textual revisions. The manuscript has seen substantial improvements in mechanistic depth, technical robustness, and presentation clarity. Accordingly, I recommend acceptance of this manuscript for publication in Nature Communications.
Below is an optional minor suggestion for final polishing, which is not required for acceptance:
1. Regarding the pan cancer correlation between GLUD1 expression and vasculogenic mimicry (VM) signatures, I recommend that the authors briefly discuss in the Discussion section the potential factors contributing to the distinct correlation strengths observed across lung cancer, breast cancer, and melanoma. Such a discussion would further strengthen the translational implications of the present study.

Version 3:

Reviewer comments:

Reviewer #1

(Remarks to the Author)
The authors have satisfactorily addressed my concerns and the m manuscript should be accepted for publication in NC.

Reviewer #2

(Remarks to the Author)
The authors have addressed all of my concerns. I now believe this manuscript meets the requirements for publication in Nature Communications.

Open Access This Peer Review File is licensed under a Creative Commons Attribution 4.0 International License, which permits use, sharing, adaptation, distribution and reproduction in any medium or format, as long as you give appropriate credit to the original author(s) and the source, provide a link to the Creative Commons license, and indicate if changes were made.
In cases where reviewers are anonymous, credit should be given to 'Anonymous Referee' and the source.
The images or other third party material in this Peer Review File are included in the article's Creative Commons license, unless indicated otherwise in a credit line to the material. If material is not included in the article's Creative Commons license and your intended use is not permitted by statutory regulation or exceeds the permitted use, you will need to obtain permission directly from the copyright holder.
To view a copy of this license, visit https://creativecommons.org/licenses/by/4.0/

Reviewer #1

1、Cancer metastasis is a complex and multistep process that requires metastatic cancer cells to at least transmigration the vessel wall twice, i.e., intravasation and extravasation. The intravasation often occurs in the primary tumor microenvironment whereas the extravasation happens in the circulation. Thus, intravasation and extravasation are two independent processes and occur in different tissues and locations. Thus, the transmigration of out-in and in-out through the vessel wall are differentially regulation in relation to the presence of primary tumors, blood flow and heterogeneity of micro vessels. In their in vitro and in vivo assays, the authors have not taken this variation into account to mechanistically address the real issue of TEM. Their both in vitro and in vivo assays are based on in the presence of primary tumors. Therefore, despite their claims, they have not addressed the issue of extravasation of tumor cells. This should be done in the absence of primary tumors. An experimental metastasis model should be employed for further investigations.

Response: We thank the reviewer for emphasizing the distinction between intravasation and extravasation. We agree that these two processes occur in different anatomical and physiological contexts. Rather than treating them as completely independent events, our study aims to identify shared functional mechanisms of tumor trans-endothelial migration that may serve as common intervention points. To prove our finding plays an important role in both intravasation and extravasation process, we used different in vitro and in vivo models to validate our concept as follows (Figure.A1).

Unpublished Data Figure Redacted

Figure A1. Schematic diagram of the in vitro and in vivo models employed throughout this study to mimic tumor cell intravasation, extravasation, and the complete metastatic cascade.

In detail explanation:

(1) Intravasation (occurs in the presence of a primary tumor)

Intravasation is defined as the process by which tumor cells invade across the endothelial barrier from the primary tissue into the circulation[1]. The reviewer correctly notes that intravasation requires a primary tumor mass. Our zebrafish yolk sac injection model recapitulates this: tumor cells form a localized aggregate (analogous to a primary tumor) within the yolk sac, then invade into the circulation. This system is powerful to examine intravasation because it has been widely used to study early metastatic dissemination and vascular entry behaviors due to its optical transparency and conserved vascular architecture[2–4]. This is complemented by a 3D microfluidic platform where tumor spheroids in the ECM channel invade into a perfused vascular channel.

(2) Extravasation (requires the absence of a primary tumor)

Extravasation refers to the process by which circulating tumor cells arrest within the vasculature, adhere to the endothelial surface, and subsequently transmigrate across the endothelial barrier into

distant tissues[5]. We agree with the reviewer that extravasation should be studied without a primary tumor. Therefore, we employed a tail vein injection into tumor-free mice, which directly delivers tumor cells into the circulation and allows quantification of extravasation independent of any primary tumor influence. This system is widely recognized as a standard approach to isolate and study extravasation, as it allows direct assessment of tumor cell arrest, endothelial interaction, and vascular exit[6,7]. In our microfluidic extravasation assay, tumor cells are perfused into the vascular lumen (not seeded in ECM), and their transmigration into the ECM is measured under flow.

(3) Orthotopic metastasis model (integrative)

In addition to the step-specific models described above, we also employed an in-situ lung tumor metastatic model in Fig. 6 as part of our intervention strategy validation. This model is commonly used to assess the complete metastatic cascade, as it combines both intravasation and extravasation within a single integrated system. It serves as a complementary validation to the step-specific models, providing the overall effect of modulating the α-KG/GLUD1/SNAIL complex on the metastatic phenotype.

To better justify our model selection and experimental logic, we have added corresponding descriptions in the revised manuscript. Specifically, the 3D in vitro model is detailed in Result 1, second paragraph; the two distinct in vivo models are described in Result 1, third paragraph; and the orthotopic metastasis model is presented in Result 6, last paragraph.

2、This reviewer is surprised with their experimental designs. They started their discovery using the vasculogenic mimicry (VM) that represents vasculature-like structures formed by tumor cells rather than vascular endothelial cells. All human sample studies and the so-called unbiased approaches are based on VM-mediated metastasis. This issue is still highly controversial. The key mechanistic issue of VM in cancer metastasis remains unresolved. How could VM be connected to the blood stream? Would these tumor cell-formed channels be connected to blood vessels? If so, why would tumor cells have to transmigrate through the vessel wall. The tumor cells could through the VM lumen to travel into the blood stream. If not, what VM has anything to do with TEM. This reviewer has difficulties to understand the rationale for dragging VM into the experimental design. Particularly, this is the initial starting point of the whole project. Designing a TEM-related project should have a novel rationale, but not just according to what is known in the literatures. Having said this, this project also lacks conceptual novelties although they have devoted great efforts to molecular characterization.

Response: We thank the reviewer for raising this fundamental conceptual concern. We fully agree that the relationship between VM and TEM must be logically justified, and we appreciate the opportunity to clarify our rationale.

(1) Clarification of the Hypothesis: VM as functional marker of TEM rather than a structural route.

We would like to clarify a critical point that may have caused misunderstanding: In our study, we do not propose that VM structures serve as direct physical conduits connecting to the bloodstream for tumor cell dissemination. Instead, we utilize the VM phenotype as a functional marker to identify tumor cell subpopulations with an enhanced capacity to interact with and traverse endothelial barriers.

The rationale for this hypothesis is as follows: While a 2015 Nature paper reported that tumor cells possess vasculature-like feature (termed vasculogenic mimicry, VM) that drive metastasis[8], as noted by the reviewer, the precise mechanism, specifically whether VM tubular networks physically connect to blood vessels, remains controversial. Drawing inspiration from recent findings in Nature where tumor cells acquire neuronal features to interact with the central nervous system[9,10], we hypothesized that tumor cells exhibiting vasculature-like features may possess an intrinsic advantage in interacting with endothelial cells. Since TEM fundamentally relies on tumor-endothelial interactions, selecting for a high-VM phenotype allows us to enrich for factors that regulate this critical interaction. Our screening strategy was thus designed to identify regulators of TEM based on this novel hypothesis, rather than on the unresolved (and for our purposes, irrelevant) question of whether VM structures physically connect to the bloodstream. Importantly, our hypothesis focuses solely on the endothelial interaction capacity conferred by VM-like phenotypes, not on any potential tubular network connectivity.

Because our screening strategy originated from this novel hypothesis, we carefully validated its credibility during the screening process. First, in our data, VM markers were enriched in aggressive tumors (Supplementary Figure 1A-1B), confirming a positive correlation between vasculature-like signatures and metastasis, consistent with previous Nature findings. Second, among the ten candidate genes identified through our integrative screening, all have been previously reported to be associated with tumor progression or metastasis (RGS16[11,12], RGS20[13], KRT18[14,15], PTTG1[16,17], PRAME[18,19], SLC7A5[20,21], POLR3G[22,23], S100A1[24], SLC20A1[25]). Notably, several genes, including PRAME[26], PTTG1[27] and POLR3G[28], have been directly implicated in VM-related processes, supporting the validity of our screening strategy in capturing functionally relevant regulators of high-VM and highly metastatic tumor behavior. In contrast, among these genes, GLUD1 had not been previously associated with this phenotype. We validated its clinical relevance by performing IHC on clinical samples and confirmed that tumors with high GLUD1 expression exhibit a significantly enhanced vasculature-like signature in lung cancer (Figure 1D-1E, as well as Figure A2(A) here). Second, during the revision process, we additionally analyzed the TCGA-based validation of the GLUD1-VM association and found that GLUD1 is positively correlated with pan-cancer vasculogenic mimicry (Figure A2 (B)).

Having identified GLUD1 through high VM and high metastatic screening, we subsequently performed systematic experiments (presented in Result 1) to validate that GLUD1 indeed mediates

TEM, and that GLUD1-mediated TEM influences both tumor cell intravasation and extravasation. This previously unreported role of GLUD1 formed the basis upon which we initiated this study, leading to our innovative findings regarding the α-KG/GLUD1/SNAIL ternary complex and identifying it as a central regulator and therapeutic target of TEM and its associated metastasis.

In summary, we believe our screening strategy is rational, and subsequent systematic experiments have confirmed that the screened protein indeed plays an important role in the TEM process.

Unpublished Data Figure Redacted

Figure A2. Clinical correlation of GLUD1 expression with vasculogenic mimicry (VM) signatures. (A) Representative immunohistochemistry images and quantitative results showing GLUD1 expression and VM marker (PAS staining) in lung cancer clinical samples. (B) TCGA-based pan-cancer analysis revealing a positive correlation between GLUD1 expression and VM-associated gene signatures across multiple cancer types, including lung, melanoma, and breast cancer.

(2) Regarding conceptual novelty

We respectfully acknowledge the reviewer's concern. Currently, TEM remains the least mechanistically defined step in the tumor metastatic cascade. Although our initial screening strategy was inspired by existing literature, the core findings of this study constitute a significant conceptual advance, specifically the identification of the α-KG/GLUD1/SNAIL ternary complex as a central regulator of TEM and the previously unrecognized non-canonical function of GLUD1 and α-KG. Notably, targeting this complex provides a potential therapeutic opportunity to simultaneously affect both tumor cell intravasation and extravasation, thereby suppressing tumor metastasis.

3. This reviewer also has another important question that remains unresolved. VE-cadherin could act has an adhesion molecule to tether cancer cells. In this regard, VE-cadherin acts as pro-metastatic adhesion molecule. TEM occurs through opening of the inter-endothelial cell junctions which are tightly sealed by VE-cadherin and other adhesion molecules. To break up this tight junction, they need to destroy or downregulate VE-cadherin. These authors have not really deal with the key mechanism of TEM. How do tumor cells go through the endothelial cell tight junctions? Unfortunately, no information has been provided to address this issue.

Response: We thank the reviewer for this critical mechanistic question. Our data directly address how tumor cells traverse endothelial tight junctions, through a three-step, cell type-specific process.

Step1 Adhesion. VE-cadherin expressed in tumor cells functions primarily as an adhesion molecule that facilitates tumor-endothelial interaction which enables tumor cells to establish stable contact with the endothelial layer[29,30]. Consistently, the GLUD1-related upregulation of VE-cadherin in tumor cells can enhance their adhesion to the endothelium (as shown in Figure 2F and supplementary Figure.3B).

Step2 Barrier destruction. It is well established that when different cell types (tumor cells, leukocytes, neutrophils) adhere to the endothelium, endothelial cells undergo rapid VE-cadherin redistribution and disassembly, leading to barrier opening[31–33]. Consistently, in our model, groups with higher tumor cell adhesion, showed greater endothelial barrier destruction (as shown in Figure 2G and supplementary Figure.3C).

Step 3 Transmigration. Increased barrier destruction allowed more tumor cells to transmigrate across the endothelium, completing TEM (as shown in Figure 2H and supplementary Figure.3D).

In summary, as the reviewer correctly noted, TEM requires dynamic regulation of VE-cadherin. However, this dynamic regulation occurs not in tumor cells but in endothelial cells, involving endothelial pathways such as Src signaling[32], p38 MAP kinase[34], and integrin signaling[35].

4. The zebrafish model is an inappropriate to investigate metastasis because the primary tumor cancer microenvironment is missing. Also, it is inappropriate to study extraversion in this model.

Response: We thank the reviewer for this important methodological concern. We agree with the reviewer that the zebrafish model has limitations, and we clarify its specific role in our study as follows:

(1) The zebrafish model is powerful to examine intravasation because it has been widely used to study early metastatic dissemination and vascular entry behaviors due to its optical transparency and conserved vascular architecture[2–4]. Thus, we use this model only for intravasation, not for extravasation.

(2) As detailed in our response to the previous comments (Figure.A1 shown above), we used tail vein injection into tumor-free mice and a 3D microfluidic extravasation assay to study extravasation.

(3) We also used an orthotopic metastasis model, which is commonly used to assess the complete

metastatic cascade, as it combines both intravasation and extravasation within a single system and preserves the native tumor microenvironment.

(4) Furthermore, we performed additional experiments showing that supplementation with microenvironmental factors such as hypoxia or TNF-α does not alter GLUD1-mediated TEM, indicating that GLUD1-driven TEM is not dependent on these specific microenvironmental cues (Figure.A3).

Unpublished Data Figure Redacted

Figure A3. Representative immunofluorescence images of adhesion, barrier-destruction and transmigration for the influence of hypoxia and TNF-α on GLUD1-mediated TEM (above). The quantification results are shown below. Scale bars: B, 20 μm; A,C, 1000 μm. Data are presented as mean ± SEM.

5. The authors completely ignore the role of tumor stroll cellular and molecular components in cancer metastasis in their studies. TAMS and CAFs all play important roles in interaction with tumor cells and blood vessels.

Response: We thank the reviewer for highlighting the important role of tumor microenvironment components, including TAMs and CAFs, in cancer metastasis.

To address whether our findings are dependent on such microenvironmental inputs, we evaluated tumor TEM under conditions incorporating tumor microenvironment related factors. Specifically, we performed TEM-related assays, including adhesion, barrier destruction, and transmigration, in the presence of TAMs and CAFs in co-culture systems (Figure A4). Across both conditions, silencing of GLUD1 consistently resulted in a marked reduction in TEM-associated phenotypes. Taken together with the results in Figures A3 and A4, although multiple factors in the tumor microenvironment indeed influence tumor metastasis, GLUD1-driven TEM is not dependent on these specific microenvironmental cues.

Unpublished Data Figure Redacted

Figure A4. Representative immunofluorescence images of adhesion, barrier-destruction and transmigration for the influence of TAMs and CAFs on GLUD1-mediated TEM (above). The quantification results are shown below. Scale bars: B, 20 µm; A,C, 1000 µm. Data are presented as mean ± SEM.

6. For extravasation, they authors have not dead with the issue of specific extravasation sites in their preferred tissues/organs.

Response: We thank the reviewer for raising this important point regarding organ-specific extravasation. We fully agree that metastatic dissemination exhibits organotropism. However, extensive evidence indicates that organ-specific metastatic preference is largely determined by primary tumor and the formation of pre-metastatic niche (PMN) driven by primary tumor, rather than by intrinsic differences in the basic process of endothelial traversal itself[36]. Primary tumors induced early changes in the microenvironment of secondary distant organs devoid of cancer cells, thereby providing a milieu favorable for cancer cell settlement and metastasis initiation[37]. Therefore, organ-selective extravasation is closely linked to pre-metastatic niche formation rather than solely reflecting tumor cell-intrinsic transmigration capacity.

In our study, we focused on identifying tumor cell-intrinsic mechanisms that regulate the ability to traverse the endothelial barrier that influence both intravasation and extravasation, rather than on organ-specific metastatic preference. Although the cell lines and animal models used in our manuscript are primarily based on lung cancer models and leptomeningeal metastasis of lung cancer, the screening process based on clinical samples in Figure 1 incorporated different tumor models.

Furthermore, Figure A3 and Figure A4 here demonstrate that GLUD1-mediated TEM is minimally influenced by the tumor microenvironment, suggesting that our findings may also play a role in other cancer types and their organ-specific metastatic processes. However, definitive conclusions require further investigation. The relevant content and limitations have been added to the Discussion section.

Reviewer #2

Remarks to the author:

Major

1.The absolute cellular α-KG concentration (not the relative concentration) is very important and requires thorough investigation. Previous studies have suggested that the mammalian cellular α-KG concentration is approximately 0.1-5 mM (PMID: 27189937; PMID: 19935646). Therefore, the absolute cellular α-KG concentrations of the cell lines used in this manuscript would be valuable. Experiments to determine the dimerization abilities of GLUD1-SNAIL under conditions with different α-KG concentrations (either in cellular or biochemical experiments) are required. The effective α-KG concentration should be relevant to the cellular or physiological concentration.

Response: We thank the reviewer for emphasizing the importance of absolute cellular α-KG concentrations and their physiological relevance. For the MST assay shown in Figure 4U, we used α-KG concentrations ranging from 0.005 mM to 15 mM, which covers the mammalian cellular α-KG concentration range of approximately 0.1-5 mM[38]. We found that α-KG binding to GLUD1 increased with increasing α-KG concentrations, with a Kd value of approximately 0.23 mM, which falls within the physiological range. In addition, we measured the cellular α-KG concentrations in our cell lines, which ranged from 50 to 500 μM, also within the physiological range. Therefore, we performed biochemical reconstitution experiments using purified proteins in the presence of a range of α-KG concentrations (50 μM, 100 μM, and 500 μM) and assessed GLUD1-SNAIL complex formation by co-immunoprecipitation. All tested concentrations of α-KG promoted GLUD1-SNAIL complex formation, with the weakest effect observed at 50 μM. In addition, we have added the concentrations of α-KG used in the figure legends of the revised manuscript, all of which are relevant to cellular or physiological concentrations.

Unpublished Data Figure Redacted

Figure A5. Purified GLUD1 and SNAIL proteins were incubated with increasing concentrations of α-KG (50, 100, and 500 μM), and complex formation was assessed by co-immunoprecipitation (Co-IP).

2. The purified GLUD1 and SNAIL proteins used in biochemical analyses should be presented with Coomassie brilliant blue-stained SDS-PAGE images.

Response: We thank the reviewer for this suggestion. The Coomassie brilliant blue-stained SDS-PAGE images of purified GLUD1 and SNAIL proteins have been included in Figure A6.

Unpublished Data Figure Redacted

Figure A6. Coomassie brilliant blue-stained SDS-PAGE of purified proteins.

3. The authors state that α-KG induces allosteric regulation of the GLUD1-SNAIL heterodimer; however, only the AI-predicted PDB models are insufficient. The authors should perform experiments to determine the complex structure of α-KG-GLUD1-SNAIL and analyze the conformational changes compared with those of the GLUD1-SNAIL complex or GLUD1 alone. Alternatively, they should revise their statement.

Response: We thank the reviewer for this important comment. We agree that determining the actual complex structure would be the gold standard to confirm the allosteric regulation. However, we believe that such structural studies (e.g., cryo-EM) are beyond the scope of the current study. Accordingly, we have revised our statements throughout the manuscript to more accurately reflect the current evidence. Specifically, in the Title and whole manuscript, we have changed "α-KG induces allosteric regulation" to "α-KG induces conformational regulation" or similar wordings. Meanwhile, in the Discussion section, we have clarified that our conclusions are based on an AI-predicted (AlphaFold) and requires experimental validation in future studies. We thank the reviewer for helping us improve the accuracy of our claims.

4. The nucleus allows molecules (<10 kDa) to freely enter and exit. The authors detected the α-KG concentration in the nucleus; how were these experiments designed and conducted? The authors should add the experimental details in the Methods section.

Response: Thanks for pointing out the omissions in our methodology. The specific method is as follows and has been included in the revised Methods section.

To quantify nuclear α-KG levels, nuclei were isolated using a rapid chilled fractionation protocol. Briefly, cells were collected in ice-cold 0.25 M sucrose buffer (containing 10 mM Tris-HCl and 3 mM $MgCl_2$, pH 7.4) and homogenized using a pre-cooled homogenizer. The homogenate was filtered and layered onto a 0.34 M sucrose cushion, followed by centrifugation at 700 g for 10 min at 4°C to pellet the nuclei. The nuclear pellet was washed twice with 0.25 M sucrose buffer at 1,000 g for 10 min to minimize cytosolic contamination. To ensure metabolic quenching and prevent α-KG leakage, the final nuclear pellet was immediately extracted with 500 µL of pre-chilled 80% (v/v) methanol at -80°C for 2 hours. After centrifugation at 15,000 g for 15 min, the supernatant was collected and vacuum-dried for subsequent LC-MS analysis. Chromatographic separation was performed on a liquid chromatography system coupled to a QTRAP 5500 mass spectrometer (AB Sciex, Framingham, MA, USA). α-KG was detected in negative electrospray ionization mode using multiple reaction monitoring (MRM). The MRM transition for α-KG was m/z 145.1 → 101.0, with declustering potential (DP) = -20 V and collision energy (CE) = -12 V. Data acquisition and quantitative analysis were performed using the manufacturer's software. Quantification was achieved by comparison with an external standard curve generated using authentic α-KG standards.

5.The authors should analyze the cross-species conservation of the key residues in Fig. 5, and the animal species used in this manuscript should be included.

Response: We thank the reviewer for these suggestions. We analyzed GLUD1 amino acid sequences from five species: Homo sapiens (human), Mus musculus (mouse), Rattus norvegicus (rat), Bos taurus (bovine), and Danio rerio (zebrafish). The key residue K147 is conserved across all five species, as shown in Figure A7.

Unpublished Data Figure Redacted

Figure A7. Cross-species conservation of the GLUD1 K147 residue.

6. K147 of GLUD1 forms a hydrogen bond with the carboxyl group of α-KG. Can dimethyl-α-KG, which lacks a carboxyl group, also form a hydrogen bond with K147? In general, this structural change would disrupt the original interaction. Since a lower concentration of dimethyl-α-KG

induced significant VE-Cad expression, the authors should add experiments to determine the Kd value of dimethyl-α-KG and discuss the potential underlying causes.

Response: We thank the reviewer for this insightful question. As the reviewer correctly noted, the carboxyl group of α-KG forms a hydrogen bond with K147 of GLUD1. Dimethyl-α-KG lacks this free carboxyl group and therefore cannot form the same hydrogen bond with K147.

Free α-KG is membrane-impermeable and cannot cross the plasma membrane to enter cells. Therefore, it is standard practice in the field to use esterified α-KG derivatives such as dimethyl-α-KG, which are cell-permeable. Once inside cells, dimethyl-α-KG is rapidly hydrolyzed by intracellular esterases to release free α-KG, which then exerts its biological functions[39,40]. Thus, dimethyl-α-KG is widely used for cellular experiments to investigate α-KG biology. In addition, we measured the Kd value of dimethyl-α-KG binding to GLUD1 and found it to be approximately 94.8 mM (Figure A8), which is orders of magnitude higher than that of free α-KG (0.23 mM). This indicates that dimethyl-α-KG does not directly interact with GLUD1 in a physiologically relevant manner. Thus, the biological effects observed at relatively low concentrations of dimethyl-α-KG are most likely mediated by its intracellular conversion to α-KG rather than by direct binding of the esterified form to GLUD1.

Unpublished Data Figure Redacted

Figure A8. MST binding assay of dimethyl-α-KG and GLUD1.

7. Diethyl glutarate can also bind to GLUD1 and exhibit competitive inhibitory activity. The structural differences between α-KG, dimethyl-α-KG, and diethyl glutarate should be discussed. It is notable that these three structurally similar chemicals exhibit significantly different results.

Response: We thank the reviewer for this insightful comment. Although α-KG, dimethyl-α-KG, and diethyl glutarate share structural similarity as dicarboxylate derivatives, their biochemical effects differ markedly. Dimethyl-α-KG and diethyl glutarate are esterified forms that can readily enter cells and undergo intracellular hydrolysis to release α-KG and glutarate, respectively[39–41]. Importantly, while glutarate can bind to GLUD1, our circular dichroism analyses revealed that it does not induce detectable conformational changes in GLUD1 (Supplementary Fig.6A in updated manuscript). In contrast, α-KG binding results in a clear alteration in the secondary structure of GLUD1 (Figure 5E in manuscript). These findings suggest that, despite their structural similarity, only α-KG can trigger the conformational changes required for enhanced GLUD1-SNAIL

interaction, whereas glutarate acts as a competitive binder. We have clarified this distinction in the revised manuscript.

8. All α-KG concentrations used in the experiments should be added to the figure captions.

Response: We thank the reviewer for this suggestion. We have now added the specific α-KG concentrations used in each experiment to the corresponding figure captions in the revised manuscript.

9. Can α-KG-like metabolites (e.g., glutamate, succinate, D/L-2HG, malate, fumarate) play a similar role or a competitive inhibitory role?

Response: We thank the reviewer for this important question. In response, we performed co-immunoprecipitation assays using purified proteins and in vitro ChIP assays in the presence of exogenously added TCA-cycle intermediates (glutamate, succinate, malate, and fumarate), with α-KG as the reference control (all metabolites were used at the same concentration: 100 μM). As shown in Figure A9, α-KG showed a clear and robust ability to promote GLUD1-SNAIL complex formation. In contrast, glutamate, succinate, and malate did not show a comparable effect. Fumarate produced only a weak band. These results indicate that the effect of promoting GLUD1 and SNAIL binding is largely specific to α-KG.

We next examined whether these α-KG-like metabolites (glutamate, succinate, D/L-2HG, malate, fumarate) exert a competitive inhibitory effect similar to that of glutarate. Using an in vitro ChIP assay, we tested whether these metabolites affect α-KG-dependent GLUD1/SNAIL binding to the CDH5 promoter. As shown in Figure A10, most of these TCA-related metabolites did not produce a significant effect. Only succinate showed a partial inhibitory effect on promoter binding (reduced by 0.75-fold), and fumarate showed a modest synergistic effect (increased by 1.59-fold). Notably, the magnitude of these effects was substantially smaller than that of glutarate (reduced by 11.99-fold; shown in Figure 6D of the manuscript).

Together, these results demonstrate that the promoting effect on GLUD1-SNAIL complex formation and CDH5 promoter binding is largely specific to α-KG, and that none of the other TCA-cycle intermediates tested phenocopy the strong inhibitory effect of glutarate.

Unpublished Data Figure Redacted

Figure A9. Specificity of α-KG in promoting GLUD1-SNAIL complex formation.

Unpublished Data Figure Redacted

Figure A10. Effects of α-KG-like metabolites on GLUD1/SNAIL binding to the CDH5 promoter. Data are presented as mean ± SEM.

Minor

1. IDH1, a NADP-dependent reversible enzyme, is one of the isocitrate dehydrogenases (IDHs). Other IDHs, such as IDH2 (also NADP-dependent) and especially IDH3 (NAD-dependent), may be key α-KG-generating enzymes because of the high cellular NAD/NADP ratio and the fact that NAD is the commonly used H+ carrier for oxidative phosphorylation. The reasons for the authors' choice of IDH1 should be presented.

Response: We thank the reviewer for this insightful comment. We agree that IDH2 ($NADP^+$-dependent, mitochondrial) and IDH3 (NAD^+-dependent, mitochondrial) are also important α-KG-generating enzymes, and that IDH3 is particularly critical for TCA cycle flux given the high cellular NAD/NADH ratio.

Our rationale for focusing on IDH1 is as follows:

First, subcellular localization: IDH1 is localized in the cytosol and peroxisomes, whereas IDH2 and IDH3 are mitochondrial. Since our study identifies that the α-KG/GLUD1/SNAIL axis functions in the nucleus to regulate transcription, the cytosolic pool of α-KG generated by IDH1 is more readily available for nuclear import compared to mitochondrial α-KG.

Second, relevance to tumor biology: IDH1 has been more frequently implicated in cancer metabolic reprogramming, including its well-known role in reductive carboxylation under hypoxia and its mutations in glioma and AML.

2.Which group do the gray dots in Fig. 5B belong to?

Response: We thank the reviewer for this question. The gray dots in the original Figure 5B represented outliers that were identified and were excluded from statistical analysis to avoid undue influence on the mean and variance. The revised figure clearly shows only the included data points. We have updated Figure 5B accordingly in the revised manuscript.

3. The marked colors in Fig. 5D are incorrect.

Response: We thank the reviewer for catching this error. The marked colors in Figure 5D have been corrected.

4. The predicted PDB models should be uploaded for free access.

Response: We thank the reviewer for this suggestion. The predicted PDB models will be made publicly available upon publication. For peer review, we have included the corresponding PDB files as supplementary attachments in the revised submission for evaluation by the reviewers and editorial office.

Reviewer #3

1.While the authors are commended for validating the role of GLUD1 using both RNAi- and overexpression-based approaches in zebrafish and murine models, the knockdown experiments rely on single siRNAs (e.g., siGLUD1, siImportin-8). In addition, much of the mechanistic work is conducted in a single cell line (A549) and its derivative. It would strengthen the study to validate the key findings using multiple siRNAs and additional cancer cell lines.

Response: We appreciate the reviewer's suggestion. We thank the reviewer for this constructive suggestion. The following points address both the siRNA and the cell line concerns.

(1) Regarding multiple siRNAs

Importantly, for the key phenotypes (GLUD1-mediated TEM ability and VE-cadherin expression, as well as Importin-8-mediated GLUD1 nuclear translocation), we validated the results using two independent siRNAs per target, which yielded consistent outcomes (data from the second siRNA are shown in Figure A11(A-H); data from the first siRNA are shown in the original figures throughout the manuscript). Moreover, for the key findings centered on GLUD1, our conclusions are supported by multiple complementary approaches, including gene silencing by siRNAs, overexpression via GLUD1 plasmid in both in vitro and in vivo assays, and various recombinant protein experiments. Together, these approaches reduce the likelihood that the observed effects arise from siRNA-specific artifacts.

(2) Regarding additional cell lines

We agree with the reviewer that much of the mechanistic work was performed in A549 or A549/DTX cells. To test generalizability across cell types, we have drawn on data from multiple experimental systems. In vivo experiments in zebrafish and murine models were conducted with multiple cell lines including H1975, PC9, H460, A549, A549/DTX, and PC9/OR, consistently supporting the association between GLUD1 and intravasation/extravasation (Supplementary Fig. 2A–2I). For GLUD1-induced VE-cadherin expression and TEM assays, we included PC9 cells for GLUD1 overexpression and PC9/OR cells for GLUD1 silencing (Supplementary Fig. 1I-1K, 3H-3M, 5D-5F). Finally, to directly address whether the key mechanistic findings such as GLUD1-

SNAIL complex formation and VE-cadherin transactivation are specific to A549 or A549/DTX cells, we have now repeated these experiments in PC9 or PC9/OR cells and obtained consistent results (Figure A11(I-J)).

(3) In addition, based on data from the TCGA database, we preliminarily observed that beyond lung cancer, GLUD1 expression also exhibits a strong positive association with VE-cadherin in the high-VM signature subgroups of melanoma and breast cancer. These results suggest that our findings may be applicable to pan-cancer contexts (Figure A11(K)), although systematic validation is still required. In the revised manuscript, we have explicitly acknowledged this limitation in the Discussion section and outlined several future directions, including: validating the α-KG/GLUD1/SNAIL axis in additional cancer types such as melanoma, breast cancer, and other highly metastatic malignancies; further assessing the functional role of GLUD1 in TEM using both in vitro and in vivo models; and determining whether targeting GLUD1 or α-KG may represent a broader therapeutic strategy in GLUD1-high tumors.

Unpublished Data Figure Redacted

Figure A11. (A) VE-Cadherin protein expression in A549/DTX processed by siNC or siGLUD1-2. (B) GLUD1 nuclear localization of A549/DTX cells processed by siNC or siIPO8-2. (C-H) Representative immunofluorescence images of adhesion (C, n=3), barrier-destruction (D, n=3) and transmigration (E, n=3) for siNC or siGLUD1-treated A549/DTX cells, with quantification results shown in F-H. For adhesion and transmigration quantification, cells were counted by FIJI. For barrier-destruction quantification, the immunofluorescence of tight junction is determined by FIJI and normalized to the 30-minute time point within each group. (I) Nuclear complex level in PC9/OR transfected with siNC or siGLUD1. (J) VE-Cadherin mRNA levels in PC9 transfected with Vector and oeGLUD1 plasmid (left) and PC9/OR

transfected with siNC or siGLUD1 (right). (K) Correlation analysis between GLUD1 and CDH5 (VE-cadherin) expression in TCGA cohorts of lung cancer, breast cancer and melanoma. Patients were stratified into high-VM and low-VM signature groups based on median expression of VM marker genes. GLUD1 expression shows a stronger positive correlation with CDH5 in the high-VM signature group compared to the low-VM signature group. R and p values are indicated. Data were analyzed using Pearson/Spearman correlation. Scale bars: B, D, 20 µm, C,E, 1000 µm.

2.The authors modulated potential effector SNAIL and downstream target VE-cadherin in the GLUD1 overexpression setting using siRNA. It is worth determining whether the rescue effects of VE-cadherin or SNAIL are specific or more pronounced in cells overexpressing GLUD1. Can VE-cadherin SNAIL knockdown alter TEM regardless of GLUD1 overexpression? In addition, it would be useful to determine whether the effects of VE-cadherin or SNAIL overexpression on TEM are diminished or overshadowed when cells are lacking GLUD1.

Response: We thank the reviewer for this important suggestion. To address the reviewer's questions: (1) whether the effects of SNAIL/VE-cadherin modulation are specific to or more pronounced in GLUD1-overexpressing cells; (2) whether SNAIL/VE-cadherin knockdown alters TEM regardless of GLUD1 status; and (3) whether the effects of SNAIL/VE-cadherin overexpression are diminished in GLUD1-deficient cells, we performed TEM assays under conditions of GLUD1 overexpression with SNAIL/VE-cadherin knockdown, as well as GLUD1 knockdown with SNAIL/VE-cadherin overexpression.

(1) Whether the effects of SNAIL/VE-cadherin modulation are specific to or more pronounced in GLUD1-overexpressing cells?

For SNAIL: The effect of SNAIL modulation is specific to GLUD1-overexpressing cells. As shown in Figure A12(D-F), SNAIL overexpression failed to rescue the reduction in TEM caused by GLUD1 silencing, suggesting that SNAIL alone is not sufficient to drive TEM in the absence of GLUD1. This conclusion aligns with our model that SNAIL must form a complex with GLUD1 to promote TEM.

For VE-cadherin: The effect of VE-cadherin modulation is not specific to GLUD1-overexpressing cells. As shown in Figure A12(J-L), overexpressing VE-cadherin in GLUD1-silenced cells rescued the declined TEM level. This finding is consistent with VE-cadherin functioning downstream of GLUD1. If other mechanisms independent of GLUD1 can upregulate VE-cadherin expression in tumor cells, they may also enhance TEM.

(2) Whether SNAIL/VE-cadherin knockdown alters TEM regardless of GLUD1 status?

As shown in Figure A12(A-C) and Figure A12(G-I), SNAIL/VE-cadherin knockdown alters TEM in the context of GLUD1 overexpression.

For SNAIL: Because SNAIL requires complex formation with GLUD1 to exert its function, in the absence of GLUD1, neither SNAIL overexpression nor knockdown has a significant effect.

For VE-cadherin: As VE-cadherin acts downstream of GLUD1, even in GLUD1-deficient cells, there may be a basal level of VE-cadherin expression driven by other mechanisms. Therefore, VE-

cadherin knockdown can still reduce TEM under these conditions.

(3) Whether the effects of SNAIL/VE-cadherin overexpression are diminished in GLUD1-deficient cells?

This point is largely consistent with the discussion in (1). As shown in Figure A12(D-F), SNAIL overexpression failed to rescue the reduction in TEM caused by GLUD1 silencing. In contrast, as shown in Figure A12(J-L), overexpressing VE-cadherin in GLUD1-silenced cells successfully rescued the declined TEM level.

In summary, these results demonstrate that SNAIL functions in a GLUD1-dependent manner, requiring complex formation with GLUD1 to promote TEM. In contrast, VE-cadherin acts as a downstream effector that can be modulated independently of GLUD1, although GLUD1 is a major upstream regulator.

Unpublished Data Figure Redacted

Figure A12. Epistatic analysis of GLUD1, SNAIL and VE-cadherin in regulating TEM. (A-C) TEM capacity in control versus GLUD1-overexpressing cells with or without SNAIL knockdown. (D-F) TEM capacity in control versus GLUD1-knockdown cells with or without SNAIL overexpression. (G-I) TEM capacity in control versus GLUD1-overexpressing cells with or without VE-Cadherin knockdown. (J-L) TEM capacity in control versus GLUD1-knockdown cells with or without VE-Cadherin overexpression. Scale bars: B,E,H,K, 20 µm; A,C,D,F,G,I,J,L, 1000 µm. Data are presented as mean ± SEM.

3. The manuscript compares nuclear and cytosolic levels of GLUD1 (Figure 3), but GLUD1 is known to reside predominantly in the mitochondria. The authors should clarify what fraction of total cellular GLUD1 is transported to the nucleus when the mitochondrial, cytosolic, and nuclear pools are considered.

Response: We thank the reviewer for this important comment. To address this, we quantified the subcellular distribution of GLUD1 in A549/DTX using immunofluorescence. Specifically, we defined nuclear regions by DAPI, mitochondrial regions by HSP60, and cytosolic regions as the remaining cellular area. Quantitative analysis revealed that approximately 60% of total GLUD1 localized to mitochondria, while ~20% localized to the nucleus and ~20% to the cytosol. To cross-validate, we analyzed the band intensities from Figure 3B. These complementary approaches consistently demonstrate that a significant non-mitochondrial GLUD1 pool exists in these cells, supporting the functional relevance of nuclear GLUD1.

Unpublished Data Figure Redacted

Figure A13. (A) Representative fluorescence images of GLUD1, HSP60, and nuclear DAPI merge in A549/DTX cells. (B) Quantitative results of fluorescence intensity of mitochondria, cell nucleus, and cytoplasm in fluorescence images, n = 4. Scale bars: A, 20 μm Data are presented as mean ± SEM

4. On a related note, it is worth knowing whether nuclear transport of GLUD1 is more prominent in metastatic cancer cells than in cells with low TEM potential. Or whether nuclear GLUD1 is higher in circulating tumor cells that have undergone intravasation or extravasation compared to primary tumors.

Response: We thank the reviewer for this insightful suggestion. As shown in Figure 3B, A549/DTX cells (high TEM potential) exhibited higher nuclear GLUD1 expression compared to parental A549 cells. In addition, we also observed higher nuclear GLUD1 levels in cells derived from metastatic lesions compared to their parental cell lines (Figure 3S in the manuscript). Furthermore, we extended our analysis to circulating tumor cells (CTCs). As shown in Figure A14, both nuclear GLUD1 and nuclear SNAIL levels were markedly elevated in CTCs compared to primary tumor cells, accompanied by a similar increasing trend in total VE-cadherin protein levels in CTCs. These findings indicate that nuclear enrichment of GLUD1 is more prominent in cells with higher TEM potential.

Unpublished Data Figure Redacted

Figure A14. Immunoblot analysis of nuclear GLUD1 and SNAIL, as well as total VE-cadherin protein levels in primary tumor cells versus circulating tumor cells (CTCs).

5. Does GLUD1 specifically and only bind to SNAIL and no other transcription factor related to EMT? It would be ideal to have experimental controls. In addition, Figures 3m, 3r, 4k, 4o, and 5g, IgG control and GLUD1 immunoprecipitation samples should be loaded in parallel for comparison. If different exposure has been used, it is difficult to compare.

Response: We thank the reviewer for these important comments.

(1) The specificity of GLUD1-SNAIL interaction.

To assess whether GLUD1 specifically binds to SNAIL or also interacts with other EMT-related transcription factors, we performed additional co-immunoprecipitation (Co-IP) experiments using nuclear extracts to examine ZEB1, SLUG, and SOX2 (TWIST was included in the original manuscript). As shown in Figure A15, GLUD1 failed to co-immunoprecipitates these transcription factors, suggesting that the interaction between GLUD1 and SNAIL is relatively selective rather than a general association with EMT-related transcription factors in the nucleus.

Unpublished Data Figure Redacted

Figure A15. Co-immunoprecipitation (Co-IP) analysis of GLUD1 with various EMT-related transcription factors (ZEB1, SLUG, SOX2, and TWIST) using nuclear extracts.

(2) Regarding the IgG control and exposure conditions in Figures 3m, 3r, 4k, 4o, and 5g.

We thank the reviewer for pointing out this technical concern. All IP and IgG group were loaded and exposed in parallel, and we present the origin data for clarification.

Unpublished Data Figure Redacted

Figure A16. Origin IP and IgG bands of Co-IP in Figure 3M, 3R, 4K, 4O.

6. In Figure 4, the R162 treatment needs an appropriate control. It is worth demonstrating whether R162 worked in these cells, possibly by assessing GLUD1 activity or intracellular a-KG levels. In addition, the authors used 50 microM, which is relatively high and may be toxic to cells, potentially reducing global protein levels. Additional controls could be included in the ChIP or biophysical assays to determine whether a-KG, but not other intermediate metabolites, specifically promotes the SNAIL-GLUD1 complex formation.

Response: We thank the reviewer for these important comments. We address each point below.

(1) The on-target activity of R162

We agree that it is critical to confirm that the compound effectively inhibits GLUD1 in our system. In our original study, mass spectrometry analysis showed that treatment with 50 μM R162 significantly reduced intracellular α-KG levels (Figure 4N), supporting that R162 is functionally active at the concentration used in our experiments. In addition, we subsequently directly measured GLUD1 enzymatic activity and confirmed that 50 μM R162 significantly suppressed the activity of wild-type GLUD1. Importantly, this inhibitory effect was abolished in the catalytic-deficient GLUD1 R496S mutant, further supporting that the effect of R162 is on-target and dependent on the catalytic activity of GLUD1 (**Figure A17**).

Unpublished Data Figure Redacted

Figure A17. Treatment with 50 μM R162 significantly reduced the enzymatic activity of wild-type GLUD1. In contrast, the enzymatically inactive GLUD1 R496S mutant already exhibited markedly impaired activity, and R162 treatment failed to induce any further reduction. Data are presented as mean ± SEM.

(2) Potential cytotoxicity of R162

We performed additional CCK-8 assays to assess cell viability following exposure to different doses of R162. We found that 50 μM R162 did not significantly affect cell viability over 24 hours. In addition, total protein levels were not noticeably reduced following R162 treatment under these conditions. These results indicate that the effects observed at 50 μM are unlikely to be due to general cytotoxicity or global suppression of protein expression.

Unpublished Data Figure Redacted

Figure A18. R162 treatment at 50 μM does not induce significant cytotoxicity or global protein reduction. (A) CCK-8 assay assessing cell viability after 24-hour treatment with indicated concentrations of R162. (B) Total protein levels in control versus 50 μM R162-treated cells. Data are presented as mean ± SEM.

(3) The specificity of α-KG in promoting GLUD1-SNAIL complex formation

We further tested a panel of TCA cycle metabolites, including glutamate, succinate, malate, and fumarate, under the same experimental conditions in a purified protein system. Among these metabolites, only α-KG robustly enhanced GLUD1-SNAIL complex formation and increased promoter binding in ChIP assays, whereas the others showed minimal or no effect. These findings indicate that the effect on GLUD1-SNAIL complex assembly is specific to α-KG rather than a general property of TCA cycle intermediates.

Unpublished Data Figure Redacted

Figure A19. α-KG specifically promotes GLUD1-SNAIL complex formation and CDH5 promoter binding. (A) Co-IP analysis of GLUD1-SNAIL complex formation in a purified protein system in the presence of α-KG or other TCA cycle intermediates (glutamate, succinate, malate, fumarate; all at 100 μM). (B) In vitro ChIP analysis of GLUD1/SNAIL binding to the CDH5 promoter under the same conditions. Data are presented as

mean ± SEM.

7. How did the authors select GLUD1 from the primary bioinformatics screening? It seems some of the representative positive or negative correlative factors are selectively shown in Figure 1c.

Response: We thank the reviewer for this important question. GLUD1 was not selected arbitrarily but emerged from a stepwise and unbiased screening strategy designed.

We first performed a pan-cancer analysis using MetMap database to identify tumors with strong metastatic potential (Lung Cancer/Melanoma) from low ones (Thyroid Cancer/Prostate Cancer). Variance analysis of TCGA clinical data revealed 3,743 differentially expressed genes between high and low VM-signature groups, designated Cohort 1. Furthermore, two additional single-cell RNA-seq data of Lung Cancer and Melanoma were adopted and divided into high-VM and low-VM subgroups based on VM programmer SERPINE2/SLPI expression, further variance analysis yielded two additional gene sets (Cohorts 2 and 3). Intersection of all three cohorts identified 102 genes. In Figure 1C, the FFS and OS log-rank P values of the 102 candidate genes are displayed, with FFS plotted on the y-axis and OS on the x-axis based on lung cancer and melanoma datasets from the KMplot database. Arrows indicate the direction of correlation with survival, with red representing positive correlation and blue representing negative correlation. Genes located in the lower-left quadrant, with both FFS and OS log-rank P values < 0.05 and showing negative correlation in both cancer types, were selected, resulting in a total of ten candidate genes for further analysis. Among these, GLUD1 is the only gene directly involved in cellular metabolism.

Additionally, ten genes (RGS16, RGS20, KRT18, PTTG1, PRAME, SLC7A5, POLR3G, S100A1, SLC20A1, and GLUD1) are all have been reported to be associated with tumor progression or metastasis in previous studies (RGS16[11,12], RGS20[13], KRT18[14,15], PTTG1[16,17], PRAME[18,19], SLC7A5[20,21], POLR3G[22,23], S100A1[24], SLC20A1[25]). Several genes (PRAME[26], PTTG1[27] and POLR3G[28]) have been directly implicated in VM-related processes which supports the validity of our screening strategy in capturing functionally relevant regulators of high-VM, highly metastatic tumor behavior.

Reviewer #4

1. In Fig 1A, the authors utilize the MetMap database to distinguish strongly metastatic (lung cancer, melanoma) from weakly metastatic tumors (thyroid cancer, prostate cancer) and observe that VM markers are enriched in aggressive tumors, leading to the conclusion that VM plays an important role in metastasis. Given the substantial inherent differences in gene expression and cellular origin across distinct tumor types, baseline VM expression may vary independently of metastatic capacity. To strengthen the rigor of this conclusion, the authors should perform a correlation analysis between VM marker expression and the incidence of metastasis from primary tumors within the same tumor

type. This additional data will further validate the functional relevance of VM in metastasis.

Response: We thank the reviewer for this insightful suggestion. To further evaluate the relationship between VM markers and tumor progression within the same tumor type, we performed additional analyses using TCGA datasets from lung cancer and melanoma. We examined the association between pathological stage and the expression of the VM marker CDH5. Across these cancer types, CDH5 expression showed a significant trend toward higher levels in stages III/IV against stage I/II shown in Figure A20(A-B). Further, to more accurately distinguish between metastatic and primary tumors and the relationship with VM markers, we used single-cell sequencing data from the GEO public dataset that clearly differentiate between metastatic and primary lung cancer and melanoma. The SERPINE2, EPHA2, PTK2 and CDH5 are up-regulated in metastatic lesions as well as GLUD1 shown in Figure A20(C-D). These results support that VM is high relevant to metastasis across Lung Cancer and Melanoma.

Unpublished Data Figure Redacted

Figure A20. VM marker expression correlates with metastasis incidence within Lung Cancer and Melanoma. (A-B) TCGA analysis showing CDH5 expression levels in stage I/II versus stage III/IV tumors in lung cancer (A) and melanoma (B). (C-D) Single-cell RNA-seq analysis (GEO) showing expression levels of VM markers (SERPINE2, EPHA2, PTK2, CDH5) and GLUD1 in primary versus metastatic lesions of lung cancer (C, GSE131907) and melanoma (D, GSE139829).

2. In Figs. 1B and 1C, the authors identified 102 genes through the intersection of three cohorts (Fig. 1B) and further narrowed down the candidates to 10 genes by correlating with overall survival (OS) and failure-free survival (FFS). Among these, GLUD1 emerged as the lead candidate and the only endogenous metabolic enzyme, prompting the authors to focus on GLUD1. The authors should analyze whether any of these 10 candidate genes have been previously reported to be implicated in VM regulation or TEM. Such an analysis would serve as valuable validation of the screening

strategy, reinforcing the biological relevance of the identified gene set and lending credence to the subsequent focus on GLUD1. Additionally, as a critical starting point for the study's data analysis, the authors need to supplement correlation data between GLUD1 and VM expression across the tumor types in the TCGA database, rather than relying solely on the IHC staining from 19 lung cancer specimens.

Response: We thank the reviewer for this insightful suggestion and have further strengthened the biological validation of our screening strategy as follows.

(1) Functional relevance of the candidate gene set

Among the 10 candidate genes identified from our integrative screening, all have been reported to be associated with tumor progression or metastasis in previous studies (RGS16[11,12], RGS20[13], KRT18[14,15], PTTG1[16,17], PRAME[18,19], SLC7A5[20,21], POLR3G[22,23], S100A1[24], SLC20A1[25]). Notably, several genes, including PRAME[26], PTTG1[27] and POLR3G[28], have been directly implicated in vasculogenic mimicry (VM)-related processes, which supports the validity of our screening strategy in capturing functionally relevant regulators of VM-associated tumor behavior, rather than reflecting random or purely prognostic correlations.

(2) TCGA-based validation of GLUD1-VM association

To further support the relevance of GLUD1 in VM-associated biology beyond our IHC cohort, we performed additional analyses using TCGA datasets (Lung Cancer, Breast Cancer and Melanoma). Correlation analysis revealed that GLUD1 expression shows a stronger positive association with CDH5 (VE-Cadherin, also a key functional marker of VM) in the high-VM signature group, whereas this correlation is markedly weaker in the low-VM signature group. These results support that GLUD1 is positively related to pan-cancer vasculogenic mimicry.

Unpublished Data Figure Redacted

Figure A21. Correlation analysis between GLUD1 and CDH5 (VE-cadherin) expression in TCGA cohorts of lung cancer, breast cancer and melanoma. Patients were stratified into high-VM and low-VM signature groups based on median expression of VM marker genes. GLUD1 expression shows a stronger positive correlation with CDH5 in the high-VM signature group compared to the low-VM signature group. R and p values are indicated. Data were analyzed using Pearson/Spearman correlation.

3. The formation of metastatic lesions is a complex process influenced by multiple factors beyond TEM. Therefore, in the murine metastasis model, quantifying the number of tumor cells adjacent to blood vessels in target organs at early time points (e.g., 24 or 48 hours) post-intravenous injection of tumor cells will more accurately reflect TEM efficiency. Furthermore, after metastatic lesion

formation, counting the number of metastatic lesions (in addition to measuring lesion area) is essential, as it partially reflects the number of colonized cells in target organs and thus indirectly indicates TEM levels.

Response: We thank the reviewer for this insightful suggestion. In the revised analysis, in addition to quantifying metastatic lesion area, we have included the number of metastatic foci as an independent parameter. This complementary metric provides a better estimate of colonization events in target organs and strengthens the evaluation of metastatic efficiency. As shown in Figure A22(A), also added as Supplementary Figure 2K in the revised manuscript, GLUD1 overexpression significantly increased the number of metastatic foci. Furthermore, we have also analyzed and included the number of metastatic foci in experiments involving DEG intervention, as shown in Figure 6J of the manuscript.

To further address early events related to tumor cell seeding, we performed in vivo bioluminescence imaging at an early time point (day 2 post-intravenous injection) to assess tumor cell distribution in the lungs, which has been previously validated to correlate with the number of tumor cells adjacent to blood vessels in target organs[7]. Quantification of total luciferase flux revealed a significant difference between the Vector and GLUD1-overexpressing groups at this early stage, supporting an effect on TEM efficiency (Figure A22(B)).

Unpublished Data Figure Redacted

Figure A22. (A) Quantification of metastatic foci number per lung section. (B) in vivo bioluminescence imaging of tumor cells at day 2 post-tail vein injection. Data are presented as mean ± SEM.

4. In Fig. 2, the authors examined three key mediators of TEM (VE-Cadherin, EPHA2, and FAK) in GLUD1-overexpressing or knockdown cells and found that EPHA2 and FAK protein levels were unaffected by GLUD1. Consequently, they excluded EPHA2 and FAK and identified VE-Cadherin as the sole downstream effector of GLUD1. This logical deduction is insufficiently rigorous: EPHA2 and FAK exert their biological functions through extensive phosphorylation-dependent activation. Thus, unchanged protein expression levels do not rule out potential regulation of their activity. The authors must supplement experiments to detect the phosphorylation levels of key functional sites in EPHA2 and FAK in GLUD1-overexpressing or knockdown cells.

Response: Thanks for pointing this out. To address this concern, we performed additional analyses examining the phosphorylation status of key functional residues of EPHA2 and FAK in GLUD1-

overexpressing cells. Specifically, we assessed phosphorylation at canonical activation sites (EPHA2 Y588 and FAK Y397), which are well-established markers of their kinase activity.

As shown in Figure A23 (also Figure 2A in revised manuscript), our results showed that GLUD1 overexpression did not lead to significant changes in the phosphorylation levels of these residues, indicating that GLUD1 does not modulate the activation state of EPHA2 or FAK at the phosphorylation level. Together with our expression analyses, this supports the conclusion that GLUD1-driven TEM is unlikely to be mediated through EPHA2 or FAK signaling and instead points to VE-Cadherin as the primary downstream effector in this context.

Unpublished Data Figure Redacted

Figure A23. GLUD1 overexpression does not affect EPHA2 or FAK phosphorylation levels.

5. In Fig. 3E, the distribution of GLUD1 in the cytoplasm needs to be presented. Quantitative analysis of the nuclear-to-cytoplasmic ratio will provide more robust support for the results. Additionally, GLUD1 expression in whole-cell lysates appears to be significantly reduced following siImportin-8 treatment, and the authors should provide a detailed explanation for this observation. In Fig. 3F, including A549 wild-type (WT) cells as a control will better facilitate the evaluation of VE-Cadherin expression upon GLUD1-NLS deletion.

Response: We thank the reviewer for these important comments. We address each point below.

(1) Distribution of GLUD1 in the cytoplasm and nuclear-to-cytoplasmic ratio quantification.

We performed quantitative analysis of the nuclear-to-cytoplasmic ratio and added the results in Supplementary Figure 4F of the revised manuscript.

(2) Effects of siImportin-8 on total GLUD1 levels

Upon re-examination, we observed that siImportin-8 treatment leads to a reduction of GLUD1 in the nuclear fraction, accompanied by an accumulation in the cytosolic fraction, consistent with impaired nuclear translocation. In addition, a modest decrease in total GLUD1 levels was also detected in whole-cell lysates. Importin-8 is known to mediate nuclear transport, and its depletion primarily affects the subcellular distribution of GLUD1. Disruption of nuclear localization may alter the stability or turnover of GLUD1, leading to a partial reduction in total protein levels[42]. Importantly, the magnitude of this decrease is limited compared to the redistribution effect observed between nuclear and cytosolic compartments.

(3) Inclusion of control in Fig. 3F

As suggested, we have included wild type cells as an additional control. As shown in Figure A24 (also included as the updated Figure 3F in the revised manuscript), to exclude baseline effects, we used Flag-tagged GLUD1. Following transfection with FLAG-GLUD1, nuclear FLAG-GLUD1 levels increased, accompanied by an increase in VE-cadherin expression. In contrast, after transfection with FLAG-GLUD1 carrying an NLS deletion, no nuclear FLAG-GLUD1 signal was detected, and VE-cadherin expression remained at baseline levels. These findings indicate that GLUD1 must enter the nucleus to promote VE-cadherin expression.

Unpublished Data Figure Redacted

Figure A24. Different localization of FLAG-GLUD1 with immunoblot assay with or without nuclear localization signal (NLS) and VE-Cadherin expression level. The NLS is 517aa-550aa of GLUD1 and determined by NLS mapper (https://nls-mapper.iab.keio.ac.jp/).

6.The ChIP assays presented in Fig. 3I and Supplementary Fig. 4F lack IgG negative controls, which are essential for assessing the specific binding intensity of GLUD1 at each SNAIL binding site. For Supplementary Fig. 4F, the ChIP results for GLUD1 at each site must be normalized to its respective input before direct comparison, as primer sets targeting distinct regions inherently exhibit variable amplification efficiencies. Furthermore, the precise location of each binding site on the VE-Cadherin promoter and its motif sequence should be provided. The same issue applies to Supplementary Fig. 4I.

Response: We thank the reviewer for highlighting these important points regarding ChIP assay rigor and data normalization. We agree that IgG controls are essential to evaluate background binding and ChIP specificity. In the revised experiments, we have included IgG immunoprecipitation controls for all ChIP assays. Meanwhile, all ChIP results in this study have been normalized to their respective inputs, and the specific calculation method is explicitly clarified in the Methods section.

The revised results are shown in Figure A25. We have also updated Figure 3I, Supplementary Figures 4G and 4K in the revised manuscript accordingly. The details of the precise location of each binding site on the VE-Cadherin promoter have been included in the figure legends in the revised manuscript.

Unpublished Data Figure Redacted

Figure A25. (A) JASPAR-predicted SNAIL binding motif1 in the VE-Cadherin promoter and level of GLUD1 binding to predicted sites of SNAIL in A549/DTX with siGLUD1 treatment versus controls, n=5. The binding motif is TGCAGGTGCT. (B) GLUD1 binding to top 4 predicted motifs of SNAIL in A549/DTX. Motif1: TCCAGGTGCT, Motif2: TCAGTTG, Motif3: TTCAAGTGGG, Motif4: GGGAAGTGGG (C) GLUD1 binding affinity to predicted sites with ChIP assay. SNAIL: TGCAGGTGCT, ZEB1/SLUG: GACACCTGCCT, TWIST: TTTACAGATGAGG, SOX2: TCACAAAGGAA. All data are normalized to input and are presented as mean ± SEM.

7. The dual luciferase reporter assays in Fig 3J lack a vector control to assess the baseline transcriptional activity of the VE-Cadherin promoter. Additionally, including data on the transcriptional activity of promoter-WT and promoter-MUT in both GLUD1-overexpressing and control cells will help evaluate the necessity of the SNAIL binding site for GLUD1-mediated VE-Cadherin transcription activation.

Response: We thank the reviewer for this important suggestion. To address this, we performed dual luciferase reporter assays in both control (Vector) and GLUD1-overexpressing (oeGLUD1) cells using the following constructs: empty vector (pGL3-Basic), VE-Cadherin promoter-WT, and VE-Cadherin promoter-MUT (SNAIL binding site mutated). As shown in Figure A26(A), the VE-Cadherin WT promoter exhibits significantly higher activity than both the empty vector and the MUT promoter, confirming that the observed signal reflects specific promoter-driven transcription. As shown in Figure A26(B), GLUD1 overexpression significantly increased WT promoter activity compared to the Vector control, whereas it did not enhance MUT promoter activity, indicating that GLUD1-mediated transcriptional activation of VE-Cadherin depends on an intact SNAIL binding site. We have included Figure A26(A) as Supplementary Figure 4I and Figure A26(B) as Figure 3J in the revised manuscript.

Unpublished Data Figure Redacted

Figure A26. GLUD1 activates VE-Cadherin transcription through the SNAIL binding site. (A) Dual luciferase

reporter assay comparing empty vector, WT-promoter and MUT-promoter. (B) Dual luciferase reporter assay in control and GLUD1-overexpressing cells using WT or MUT promoters. Data are presented as mean ± SEM.

8. In Fig. 3L, the Co-IP assay results appear to indicate that the GLUD1-SNAIL interaction is not restricted to the nucleus but also occurs in the cytoplasm. The apparent absence of signal in the middle region of the cytoplasmic fraction blot is likely an experimental artifact—potentially arising from band distortion, uneven protein loading, or suboptimal exposure conditions. The authors are advised to carefully re-examine their original raw data to confirm the authenticity of the cytoplasmic GLUD1-SNAIL interaction and rule out technical artifacts.

Response: We thank the reviewer for this careful evaluation. To exclude the possibility that the cytoplasmic GLUD1-SNAIL interaction arose from technical artifacts, we repeated the Co-IP experiments using independent biological replicates and carefully controlled protein loading and exposure conditions. As shown in Figure A27, the interaction signal in the cytoplasmic fraction was consistently reproducible and not attributable to band distortion, uneven loading, or suboptimal exposure. Notably, the interaction detected in the cytoplasmic fraction was substantially weaker than that observed in the nuclear fraction, indicating that GLUD1-SNAIL association is predominantly nuclear. The detectable interaction in the cytoplasmic fraction is biologically plausible, given that abundant α-KG and GLUD1 are present in the cytoplasm, and even a small amount of SNAIL localized outside the nucleus could facilitate GLUD1-SNAIL association in this compartment. We have replaced the original figure with the updated Co-IP results in the revised manuscript.

Unpublished Data Figure Redacted

Figure A27. GLUD1-SNAIL interaction occurs predominantly in the nucleus.

9. While the authors demonstrated the interaction between GLUD1 and SNAIL in Fig. 3, they did not confirm whether this binding is required for SNAIL's binding to the VE-Cadherin promoter. The authors need to supplement ChIP data to show SNAIL binding to the VE-Cadherin promoter under GLUD1 overexpression or knockdown conditions, as well as data on SNAIL-mediated regulation of VE-Cadherin promoter activity.

Response: We thank the reviewer for this important suggestion. To determine whether GLUD1 is required for SNAIL binding to the VE-Cadherin promoter, we performed ChIP-qPCR assays for SNAIL under GLUD1 knockdown conditions. As shown in Figure A28, we found that depletion of GLUD1 led to a marked reduction of SNAIL occupancy at the VE-Cadherin promoter, indicating that SNAIL binding is dependent on the presence of GLUD1.

Consistently, sequential ChIP (ChIP-reChIP) assays in Figure 3K of manuscript, using anti-GLUD1 followed by anti-SNAIL antibodies revealed robust enrichment at the promoter compared to GLUD1/IgG controls, whereas this enrichment was substantially diminished upon GLUD1 knockdown. These findings demonstrate that GLUD1 is indispensable for efficient recruitment of SNAIL to the VE-Cadherin promoter and support the notion that formation of the GLUD1/SNAIL complex is critical for their binding to the VE-Cadherin promoter.

Although we did not perform experiments on SNAIL-mediated regulation of VE-cadherin promoter activity, we synthesized recombinant promoter DNA sequences and incubated them with different combinations of α-KG, recombinant GLUD1, and recombinant SNAIL. Following pull-down assays, we quantified the binding affinity of the GLUD1/SNAIL complex for the promoter DNA via qPCR (Figure 4S). As shown in Figure 4T, neither GLUD1 nor SNAIL alone recruited the VE-cadherin promoter. Only when α-KG, GLUD1, and SNAIL were all present—allowing the formation of the ternary complex—was significant promoter enrichment detected. This result provides very solid evidence that the interaction between GLUD1 and SNAIL is required for SNAIL's binding to the VE-cadherin promoter.

Unpublished Data Figure Redacted

Figure A28. GLUD1 is required for SNAIL recruitment to the VE-Cadherin promoter. ChIP-qPCR analysis of SNAIL occupancy at the VE-Cadherin promoter in control versus GLUD1-knockdown (GLUD1-KD) cells. GLUD1 depletion markedly reduces SNAIL binding. Data are presented as mean ± SEM.

10. In Fig. 3R, the authors state that the interaction between GLUD1 and SNAIL was markedly enriched in metastatic tumors compared to contralateral normal lung tissues. However, the data do not show striking differences in the expression levels of GLUD1 or SNAIL between metastatic tumors and normal lung tissues. The authors should explain why the GLUD1-SNAIL interaction is specifically enhanced in metastatic tumors despite similar protein expression levels.

Response: we thank the reviewer for this important comment. In our Co-IP experiments, equal amounts of total protein from normal and metastatic tumor tissues were used as input for immunoprecipitation, ensuring that the observed differences in GLUD1-SNAIL interaction are not

attributable to unequal loading. The input lanes shown in the initial figure were derived from these equalized samples to ensure comparability of the IP assay. To better reflect the endogenous expression levels of GLUD1 and SNAIL, we have now included nucleus protein amount in the Figure 3R of revised manuscript (as also shown in Figure A29 below). These data show that both GLUD1 and SNAIL tend to be expressed at higher levels in metastatic tumor tissues compared to normal tissues. Therefore, the enhanced GLUD1-SNAIL interaction in metastatic tumors is consistent with both (i) higher endogenous expression levels of GLUD1 and SNAIL, and (ii) elevated α-KG levels in metastatic tumors, which acts as a conformational stabilizer of complex formation.

Unpublished Data Figure Redacted

Figure A29. Endogenous nuclear expression of GLUD1 and SNAIL and their interaction in normal lung versus metastatic tumor tissues.

11. In Fig 4A-N, the authors observe that the GLUD1 enzymatic inhibitor R162 attenuates the GLUD1-SNAIL interaction and TEM phenotypes. From these findings, they propose that "the reduction of nuclear α-KG resulted by R162 is involved in the regulation of complex assembly." However, this logical inference lacks sufficient experimental substantiation and overlooks alternative mechanisms. Critically, R162 binding to GLUD1 may directly disrupt the GLUD1-SNAIL interaction by altering GLUD1's conformation or interfering with their binding interface, independent of its effect on α-KG production. The authors must rule out these alternative possibilities. Specifically, they need to demonstrate that R162 binding to GLUD1 does not affect GLUD1’s conformation or the GLUD1-SNAIL binding interface. Two complementary experimental strategies would be effective: (1) constructing a catalytically inactive GLUD1 mutant (via point mutation at the enzymatic active site) and repeating the key experiments to determine if R162 still attenuates the GLUD1-SNAIL interaction in the absence of enzymatic activity; or (2) performing in vitro pulldown assays with purified recombinant GLUD1 and SNAIL proteins to confirm that R162 does not directly interfere with their binding. Additionally, the authors need to provide quantitative data on intracellular α-KG levels following R162 treatment.

Response: We thank the reviewer for raising this important concern. To determine whether R162 attenuates the GLUD1-SNAIL interaction through inhibition of GLUD1 enzymatic activity or via

direct structural disruption of the binding interface, we generated a catalytically inactive GLUD1 mutant (R496S) in A549 cells[43].

As shown in Figure A30, upon R162 treatment, we observed that R162 no longer attenuated the interaction between GLUD1 and SNAIL in the catalytic mutant background. In contrast, R162 significantly reduced the interaction in wild-type GLUD1-expressing cells (Figure 4K in manuscript). This result indicates that the effect of R162 on GLUD1-SNAIL complex formation is dependent on GLUD1 enzymatic activity, rather than resulting from a direct structural disruption of the GLUD1/SNAIL binding interface. In the revised manuscript, we have included Figure A30 as Supplementary Figure 5I.

To further support this conclusion, we utilized two additional independent pharmacological approaches:

(1) Another GLUD1 inhibitor (EGCG). As shown in Supplementary Fig. 5A-5C, treatment with EGCG, a structurally distinct GLUD1 inhibitor, similarly attenuated the VE-Cadherin expression, consistent with the effects observed with R162.

(2) IDH inhibitor (reducing α-KG production). As shown in Supplementary Fig. 5G-5H, treatment with an IDH inhibitor, which reduces intracellular α-KG levels by blocking α-KG production upstream of GLUD1, also diminished the GLUD1/SNAIL interaction and TEM phenotypes.

Taken together, these complementary approaches, including catalytically inactive GLUD1 mutant, a structurally distinct GLUD1 inhibitor (EGCG), and an IDH inhibitor that lowers α-KG independently of GLUD1, collectively demonstrate that the reduction of GLUD1/SNAIL complex formation is mediated by decreased α-KG availability rather than direct compound-induced structural interference with the GLUD1/SNAIL binding interface.

Additionally, quantitative data on intracellular α-KG levels following R162 and IDH inhibitor treatment are provided in Figure 4N and Supplementary Fig. 5G, respectively, of the revised manuscript.

Unpublished Data Figure Redacted

Figure A30. Co-IP analysis of the effect of R162 on GLUD1-SNAIL interaction in A549 cells expressing catalytically inactive GLUD1 mutant (R496S).

12. In Figs. 4K-N, although the authors demonstrated that α-KG can directly enhance GLUD1-SNAIL binding, there remains a logical gap in this reasoning. As a key intermediate metabolite, α-KG regulates multiple metabolic and downstream signaling pathways. Thus, the ability of α-KG to rescue the R162-induced reduction in GLUD1-SNAIL binding does not directly confirm that α-KG enhances their interaction directly. The authors should supplement experiments to show that inhibitors of enzymes directly responsible for α-KG metabolism do not affect this process, or at least provide a detailed discussion addressing this caveat.

Response: We thank the reviewer for this important point. To address whether α-KG directly promotes GLUD1-SNAIL interaction or acts through other metabolic pathways, we performed the following experiments.

(1) We inhibited two major α-KG-producing enzymes, GLUD1 and IDH1, using R162 and (R,R)-GSK321, respectively. Both treatments reduced GLUD1-SNAIL interaction, indicating that intracellular α-KG availability contributes to efficient complex formation in cells (Figure 4K for GLUD1 inhibitor and Supplementary Fig. 5G-5H for IDH inhibitor).

(2) Following the reviewer's suggestion, we further tested a panel of TCA cycle metabolites, including glutamate, succinate, malate, and fumarate, under the same experimental conditions in a purified protein system. Among these metabolites, only α-KG robustly enhanced GLUD1-SNAIL complex formation and increased promoter binding in ChIP assays, whereas the others showed minimal or no effect (Figure A31(A-B)). These findings indicate that the effect on GLUD1-SNAIL complex assembly is specific to α-KG rather than a general property of TCA cycle intermediates. We have included these results in Supplementary Figures 5J and 5K of the manuscript.

(3) To further assess structural specificity, we examined these TCA cycle metabolites for their ability to induce conformational changes in GLUD1 using circular dichroism (CD) spectroscopy. Notably, none of the other metabolites induced comparable secondary structural changes in GLUD1, consistent with the functional specificity observed in the Co-IP and ChIP assays (Figure A31(C)).

(4) Consistently, our in vitro binding assays using purified proteins demonstrated that α-KG directly enhances GLUD1-SNAIL interaction and complex/promoter binding in a cell-free system (Figure 4R-4T), effectively ruling out the involvement of other metabolites.

Taken together, these results support that α-KG directly and specifically promotes GLUD1-SNAIL interaction.

Unpublished Data Figure Redacted

Figure A31. α-KG specifically promotes GLUD1-SNAIL complex formation and CDH5 promoter binding. (A)

Co-IP analysis of GLUD1-SNAIL complex formation in a purified protein system in the presence of α-KG or other TCA cycle intermediates (glutamate, succinate, malate, fumarate; all at 100 µM). (B) In vitro ChIP analysis of GLUD1/SNAIL binding to the CDH5 promoter under the same conditions. (C) Circular dichroism results of purified GLUD1 added with glutamate, succinate, malate, fumarate; all at 100 µM. Data are presented as mean ± SEM.

13. While the authors demonstrated that the α-KG-GLUD1-SNAIL ternary complex promotes SNAIL binding to the VE-Cadherin promoter and enhances its transcription, the underlying mechanism remains unclear. Does the complex enhance SNAIL's DNA-binding affinity, protect SNAIL from degradation, or exert other effects? The authors should propose a plausible mechanism.

Response: We thank the reviewer for this important mechanistic question. Neither genetic (siGLUD1) nor pharmacological (R162) inhibition of GLUD1 enzymatic activity caused an obvious change in SNAIL protein levels, whether in vitro or in vivo (Figure 3M, 4K, 4R in manuscript), suggesting that GLUD1 does not regulate this process mainly through controlling SNAIL abundance or degradation.

In addition, we employed an in vitro reconstitution system using purified GLUD1, SNAIL, and VE-Cadherin promoter DNA, which lacks the cellular machinery required for protein degradation. Notably, we found that SNAIL alone showed minimal binding to the promoter, whereas the addition of both GLUD1 and α-KG enabled robust association of SNAIL with the promoter (Figure 4S-4T in manuscript). Therefore, our results suggest that the complex enhance SNAIL's DNA-binding affinity.

14. Glutarate mediates competitive inhibition of α-ketoglutarate (α-KG)-dependent metabolic enzymes via structural mimicry of α-KG: it occupies the enzyme's active site, thereby precluding α-KG binding and perturbing the catalytic cycle. Canonically, α-KG undergoes oxidative decarboxylation during enzymatic catalysis, providing energy and functional chemical groups essential for substrate modifications such as hydroxylation and demethylation. In contrast, glutarate lacks the critical α-keto group, rendering it unable to initiate oxidative decarboxylation. This deficiency leads to arrest of the enzyme's catalytic cycle and results in irreversible "non-productive binding-mediated inhibition"—a hallmark of its canonical inhibitory mechanism. However, the binding of α-KG to GLUD1, as delineated in this study, is distinct in that it does not entail oxidative decarboxylation. Thus, the well-established competitive inhibition paradigm for glutarate cannot be directly extrapolated to its interaction with GLUD1. In fact, Fig. 5 shows that α-KG binding induces allosteric conformational changes in GLUD1. As a structural analog of α-KG, glutarate would theoretically be expected to exert a similar effect. The authors must supplement experiments to clarify why glutarate binding to GLUD1 fails to induce the same α-KG-like allosteric conformational changes that promote GLUD1-SNAIL complex formation. Without this clarification, it is reasonable to hypothesize that the anti-metastatic effects of glutarate may be mediated by its

inhibition of other α-KG-dependent metabolic enzymes rather than through direct interference with the α-KG/GLUD1/SNAIL axis.

Response: We thank the reviewer for this insightful comment. Given its structural similarity to α-KG, glutarate could in principle interact with GLUD1. To address why glutarate does not phenocopy α-KG in promoting GLUD1-SNAIL complex formation, we performed additional circular dichroism (CD) analyses to assess the conformational response of GLUD1 upon glutarate treatment. As shown in Figure A32, in contrast to α-KG, glutarate did not induce an obvious secondary structural change in GLUD1. Importantly, this was not unique to glutarate. We also tested other α-KG-like metabolites, including glutamate, succinate, malate, and fumarate, under the same CD spectroscopy conditions. None of these metabolites induced comparable conformational changes in GLUD1, consistent with their inability to promote GLUD1-SNAIL complex formation in our functional assays. These results indicate that binding alone is not sufficient; rather, the conformational change induced upon binding is the critical determinant for promoting GLUD1-SNAIL complex assembly.

Furthermore, in Figure 6, we found that mutating the glutarate-binding site in GLUD1 effectively reversed the inhibitory effects of glutarate. This result collectively indicates that the anti-metastatic effects of glutarate are unlikely to be mediated through inhibition of other α-KG-dependent metabolic enzymes.

Unpublished Data Figure Redacted

Figure A32. Circular dichroism results of purified GLUD1 added with 100 μM α-KG (A), Glutarate (B) and other α-KG-like metabolites (C).

15. While diethyl glutarate (DEG) exhibits promising anti-metastatic efficacy, its long-term toxicity, metabolic stability, and tissue distribution have not been evaluated. The authors should temper their claims regarding its therapeutic potential or include preliminary toxicity data (e.g., changes in body weight, organ histology) from the murine studies.

Response: We thank the reviewer for this important comment. We agree that the long-term toxicity, metabolic stability, and tissue distribution of diethyl glutarate (DEG) require further systematic investigation.

Diethyl glutarate (DEG), as a membrane-permeable esterified form of glutarate, can be rapidly metabolized to generate glutarate in vivo. DEG has been used as a therapeutic strategy to modulate T-cell metabolism and anti-tumour immunity[41]. In that study, the authors administered DEG at 10 mg/kg every 2–3 days and observed no overt toxicity. Of note, the dosing regimen used in our

study is comparable to that regimen.

Furthermore, glutarate itself is an endogenous metabolic intermediate that is normally metabolized under physiological conditions. Pathological accumulation of glutarate, leading to toxicity, occurs only in extreme settings such as hereditary glutaric aciduria type I, where a genetic enzyme defect causes a several-hundred-fold increase in glutarate levels. Therefore, administration of DEG at the doses used in our study is unlikely to reach such pathological concentrations, supporting its favorable safety profile under the tested conditions.

To further address the reviewer's concern, we have now performed H&E staining of major normal organs following long-term DEG treatment (related to Figure 6L-6O in the revised manuscript). As shown in new Figure A33, we did not observe apparent histopathological toxicity in major tissues at the tested concentration.

Based on the previously reported findings and our preliminary toxicity data from the murine studies, we believe that DEG exhibits a favorable safety profile under the dosing conditions where therapeutic efficacy is observed. Accordingly, we have tempered our discussion regarding the therapeutic potential of DEG in the revised manuscript and now emphasize that further pharmacological and toxicological characterization will be necessary before clinical translation.

Unpublished Data Figure Redacted

Figure A33 H&E staining of major normal organs from the orthotopic lung tumor model following DEG treatment (related to Figure 6L-6O in revised manuscript) (10 mg/kg, i.p., every three days).

16. The authors identified GLUD1 as a key mediator of TEM through integrated analysis across multiple tumor types (lung cancer, melanoma, thyroid cancer and prostate cancer). However, subsequent phenotypic and mechanistic experiments focused solely on lung cancer. Thus, it remains unclear whether the α-KG/GLUD1/SNAIL axis operates similarly in other tumor types. The authors should either include preliminary validation data in additional cancer types (e.g., melanoma, prostate cancer) or explicitly discuss this limitation and propose future research directions.

Response: We thank the reviewer for this important comment. We agree that the relevance of the

α-KG/GLUD1/SNAIL axis in other tumor types remains to be determined, as our mechanistic studies focused primarily on lung cancer models. In the revised manuscript, we have explicitly acknowledged this limitation in the Discussion section and outlined the following future directions, including validating the axis in additional cancer types such as melanoma, breast cancer and other Malignant tumors assessing the functional role of GLUD1 in TEM using in vitro/vivo models, and determining whether targeting GLUD1 or α-KG has therapeutic potential in other GLUD1-high tumors. We thank the reviewer for helping us identify this important direction for future studies.

Minor

1. In Supplementary Fig. 5E, the image of the control group at 60 minutes is inconsistent with its statistical result. The authors should carefully verify this data. Additionally, these results (along with those in Fig. 4F) are not described or discussed in the manuscript.

Response: We thank the reviewer for carefully examining this point. All quantitative analyses were performed after normalization to the 30-minute time point within each group. Therefore, the statistical results reflect relative changes normalized to this internal reference, which may not be directly apparent from the representative images shown. We have optimized the description in the revised manuscript to clarify this normalization method. In addition, we have added the missing descriptions and discussions for these results (Supplementary Fig. 5E and Fig. 4F) in the revised manuscript.

2. Many experimental details are missing from the Figure legends, making it difficult to follow the methodology, particularly for the ChIP and dual luciferase reporter assays.

Response: We thank the reviewer for pointing this out. We have carefully revised and expanded the figure legends in the revised manuscript, with particular attention to the ChIP and dual luciferase reporter assays, to ensure that all essential experimental details are clearly described.

3.Lines 237-238 contain an erroneous description: "R162 also elevated tumor cell adhesion, barrier-destruction and transmigration across endothelial cells." Based on the study’s findings, R162 should inhibit TEM-related processes, and this statement should be corrected accordingly.

Response: We thank the reviewer for catching this error. We have corrected the statement in the revised manuscript, changing "elevated" to "reduced" to accurately reflect that R162 inhibits TEM-related processes.

4. Several linguistic expressions throughout the manuscript require refinement to enhance precision, clarity, and conformity with scientific writing conventions.

Response: We thank the reviewer for this suggestion. We have carefully reviewed the entire manuscript and refined the language to enhance precision, clarity, and adherence to scientific writing

conventions. The manuscript has been edited accordingly.

Reference

1. Gerstberger, S., Jiang, Q. & Ganesh, K. Metastasis. *Cell* **186**, 1564–1579 (2023).
2. White, R., Rose, K. & Zon, L. Zebrafish cancer: the state of the art and the path forward. *Nat Rev Cancer* **13**, 624–636 (2013).
3. Weiss, J. M. *et al.* Anatomic position determines oncogenic specificity in melanoma. *Nature* **604**, 354–361 (2022).
4. Hunter, M. V. *et al.* Mechanical confinement governs phenotypic plasticity in melanoma. *Nature* **647**, 517–527 (2025).
5. Reymond, N., d'Água, B. B. & Ridley, A. J. Crossing the endothelial barrier during metastasis. *Nat Rev Cancer* **13**, 858–870 (2013).
6. Adrover, J. M. *et al.* Neutrophils drive vascular occlusion, tumour necrosis and metastasis. *Nature* **645**, 484–495 (2025).
7. Huang, Y. *et al.* Leptomeningeal metastatic cancer cells induce a permissive choroid plexus vasculature through extracellular-vesicle-derived 5-HIAA signaling. *Nat Cancer* https://doi.org/10.1038/s43018-026-01145-y (2026) doi:10.1038/s43018-026-01145-y.
8. Wagenblast, E. *et al.* A model of breast cancer heterogeneity reveals vascular mimicry as a driver of metastasis. *Nature* **520**, 358–362 (2015).
9. Peinado, P. *et al.* Intrinsic electrical activity drives small-cell lung cancer progression. *Nature* **639**, 765–775 (2025).
10. Zhi, X. *et al.* Nociceptive neurons promote gastric tumour progression via a CGRP-RAMP1 axis. *Nature* **640**, 802–810 (2025).

11. Zhang, Y., Zhu, Q., Cao, X. & Ni, B. RGS16 regulates Hippo-YAP activity to promote esophageal cancer cell proliferation and migration. *Biochemical and Biophysical Research Communications* **675**, 122–129 (2023).

12. Huang, R. *et al.* RGS16 promotes glioma progression and serves as a prognostic factor. *CNS Neurosci Ther* **26**, 791–803 (2020).

13. Huang, G., He, X. & Wei, X.-L. lncRNA NEAT1 promotes cell proliferation and invasion by regulating miR-365/RGS20 in oral squamous cell carcinoma. *Oncol Rep* **39**, 1948–1956 (2018).

14. Li, G. *et al.* Keratin gene signature expression drives epithelial-mesenchymal transition through enhanced TGF-β signaling pathway activation and correlates with adverse prognosis in lung adenocarcinoma. *Heliyon* **10**, e24549 (2024).

15. Wang, P.-B., Chen, Y., Ding, G.-R., Du, H.-W. & Fan, H.-Y. Keratin 18 induces proliferation, migration, and invasion in gastric cancer via the MAPK signalling pathway. *Clinical and Experimental Pharmacology and Physiology* **48**, 147–156 (2021).

16. Huang, J. *et al.* The long non-coding RNA PTTG3P promotes cell growth and metastasis via up-regulating PTTG1 and activating PI3K/AKT signaling in hepatocellular carcinoma. *Mol Cancer* **17**, 93 (2018).

17. Xiang, W. *et al.* PTTG1 regulated by miR-146a-3p promotes bladder cancer migration, invasion, metastasis and growth. *Oncotarget* **8**, 664–678 (2016).

18. Cai, L. *et al.* Gene Expression Profiling and *PRAME* Status Versus Tumor-Node-Metastasis Staging for Prognostication in Uveal Melanoma. *American Journal of Ophthalmology* **195**, 154–160 (2018).

19. Field, M. G. *et al.* PRAME as an Independent Biomarker for Metastasis in Uveal Melanoma.

Clin Cancer Res **22**, 1234–1242 (2016).

20. Ding, K. *et al.* GSE1 predicts poor survival outcome in gastric cancer patients by SLC7A5 enhancement of tumor growth and metastasis. *Journal of Biological Chemistry* **293**, 3949–3964 (2018).
21. Wang, J. *et al.* SLC7A5 Functions as a Downstream Target Modulated by CRKL in Metastasis Process of Gastric Cancer SGC-7901 Cells. *PLOS ONE* **11**, e0166147 (2016).
22. Chen, H., Ma, L., Yang, W., Li, Y. & Ji, Z. POLR3G promotes EMT via PI3K/AKT signaling pathway in bladder cancer. *The FASEB Journal* **37**, e23260 (2023).
23. Lautré, W., Richard, E., Feugeas, J.-P., Dumay-Odelot, H. & Teichmann, M. The POLR3G Subunit of Human RNA Polymerase III Regulates Tumorigenesis and Metastasis in Triple-Negative Breast Cancer. *Cancers* **14**, (2022).
24. Tian, T., Liu, Z., Chen, H. & Cui, Z. S100A1 Promotes Cell Proliferation and Migration and Is Associated with Lymph Node Metastasis in Ovarian Cancer. *Discovery Medicine* **23**, 235–245 (2017).
25. Onaga, C. *et al.* High expression of SLC20A1 is less effective for endocrine therapy and predicts late recurrence in ER-positive breast cancer. *PLOS ONE* **17**, e0268799 (2022).
26. Vartanian, A. *et al.* Inhibitor of vasculogenic mimicry restores sensitivity of resistant melanoma cells to DNA-damaging agents. *Melanoma Research* **27**, 8 (2017).
27. Cui, L. *et al.* Suppression of PTTG1 inhibits cell angiogenesis, migration and invasion in glioma cells. *Med Oncol* **37**, 73 (2020).
28. Fan, C. *et al.* CircCCNB1 inhibits vasculogenic mimicry by sequestering NF90 to promote miR-15b-5p and miR-7-1-3p processing in nasopharyngeal carcinoma. *Mol Oncol* **19**, 1876–

1893 (2025).

29. Brock, T. *et al.* The Influence of VE-Cadherin on Adhesion and Incorporation of Breast Cancer Cells into Vascular Endothelium. *International Journal of Molecular Sciences* **22**, 6049 (2021).

30. Le Guelte, A., Dwyer, J. & Gavard, J. Jumping the barrier: VE-cadherin, VEGF and other angiogenic modifiers in cancer. *Biol Cell* **103**, 593–605 (2011).

31. Aragon-Sanabria, V. *et al.* VE-Cadherin Disassembly and Cell Contractility in the Endothelium are Necessary for Barrier Disruption Induced by Tumor Cells. *Sci Rep* **7**, 45835 (2017).

32. Alcaide, P. *et al.* p120-Catenin prevents neutrophil transmigration independently of RhoA inhibition by impairing Src dependent VE-cadherin phosphorylation. *Am J Physiol Cell Physiol* **303**, C385-395 (2012).

33. Schulte, D. *et al.* Stabilizing the VE-cadherin–catenin complex blocks leukocyte extravasation and vascular permeability. *EMBO J* **30**, 4157–4170 (2011).

34. Khanna, P. *et al.* p38 MAP kinase is necessary for melanoma-mediated regulation of VE-cadherin disassembly. *Am J Physiol Cell Physiol* **298**, C1140-1150 (2010).

35. Huang, R.-L. *et al.* ANGPTL4 modulates vascular junction integrity by integrin signaling and disruption of intercellular VE-cadherin and claudin-5 clusters. *Blood* **118**, 3990–4002 (2011).

36. Peinado, H. *et al.* Pre-metastatic niches: organ-specific homes for metastases. *Nat Rev Cancer* **17**, 302–317 (2017).

37. Patras, L., Shaashua, L., Matei, I. & Lyden, D. Immune determinants of the pre-metastatic niche. *Cancer Cell* **41**, 546–572 (2023).

38. Altea-Manzano, P., Cuadros, A. M., Broadfield, L. A. & Fendt, S. Nutrient metabolism and cancer in the in vivo context: a metabolic game of give and take. *EMBO Rep* **21**, e50635 (2020).

39. Parker, S. J. *et al.* Spontaneous hydrolysis and spurious metabolic properties of α-ketoglutarate esters. *Nat Commun* **12**, 4905 (2021).

40. Triggering and amplification of insulin secretion by dimethyl α-ketoglutarate, a membrane permeable α-ketoglutarate analogue. *European Journal of Pharmacology* **607**, 41–46 (2009).

41. Minogue, E. *et al.* Glutarate regulates T cell metabolism and anti-tumour immunity. *Nat Metab* **5**, 1747–1764 (2023).

42. Oka, M. & Yoneda, Y. Importin α: functions as a nuclear transport factor and beyond. *Proc. Jpn. Acad., Ser. B* **94**, 259–274 (2018).

43. Yang, R. *et al.* Glutamate dehydrogenase 1-catalytic glutaminolysis feedback activates EGFR/PI3K/AKT pathway and reprograms glioblastoma metabolism. *Neuro Oncol* **27**, 668–681 (2025).

Reviewer #1

The authors have performed additional experiments to address my previous concerns. In general, the revised manuscript is improved. However, some of my previous concerns related to distinct steps of cancer metastasis remain.

1. The authors performed new experiments using a zebrafish metastasis model. There are some issues about the zebrafish model. a) This model lacks a truly primary tumor and a clinically relevant tumor microenvironment; b) Injection of cancer cells into the yolk sac of developing embryos does not really allow the authors to study the process of intravasation. All these processes occur through passive processed and not related to the invasiveness of cancer cells. Usually, cancer cells are implanted into the intervitelline space that allows cancer cell invasion and intravasate. Also, some excellent and important publications in zebrafish metastasis have not been cited.

Response: We thank the reviewer for the constructive comments on the zebrafish metastasis model. We fully acknowledge the limitations pointed out and have revised the manuscript accordingly to clarify the scope and interpretation of the zebrafish experiments.

(1) The limitation of zebrafish metastasis model.

We agree with the reviewer that the zebrafish xenograft model does not recapitulate a true primary tumor or the full complexity of the mammalian tumor microenvironment. In our study, we did not intend to use this model to study the complete metastatic cascade. Instead, we employed it solely as a rapid in vivo system to assess the intravasation potential of tumor cells. Importantly, we have performed additional experiments to validate our main conclusions under conditions that more closely mimic the mammalian tumor microenvironment. Furthermore, we have included primary tumor-based metastasis assays (now presented in Figure 6), which strongly support our key findings without relying on the zebrafish model.

(2) Injection site (yolk sac vs. intervitelline space) and intravasation.

We thank the reviewer for this important technical guidance. Although yolk sac implantation is a well-established zebrafish model for evaluating tumor cell intravasation[1], previous studies have noted that cell leakage associated with the injection site may represent a potential limitation[2,3]. Because implantation into the intervitelline space provides a more stringent assessment of tumor cell entry into the circulation, we have now performed additional zebrafish experiments using this approach. As shown in Figure B1, GLUD1 indeed serves as a key mediator of intravasation in this setting, further supporting our conclusions in **Figure 1**. We have now incorporated these new data into the revised manuscript and have cited the recommended references on zebrafish metastasis models. The relevant results have been added as Supplementary Fig. 2R in the revised manuscript.

Unpublished Data Figure Redacted

Figure B1. Quality control of zebrafish xenograft implantation and assessment of tumor cell intravasation. (A) Representative image showing localized implantation of tumor cells in the intervitelline space of zebrafish larvae. At 12 h post-injection, larvae with correctly localized tumor cell engraftment and without overt early dissemination into the circulation were retained for further analysis. (B) The migration distance of A549 cells transfected with Vector/oeGLUD1 plasmid.

2. The authors explained that VM was used as a marker to predict metastasis but not a structure for metastasis. However, the mechanistic link of this marker to invasiveness of cancer cells in to the circulation is hard-to-understand. Perhaps, the authors should clearly indicate that VM has been used as morphological markers and the mechanistic link is missing.

Response: We thank the reviewer for this important clarification. We have now revised the manuscript to clearly state that VM was used solely as a morphological marker to predict enhanced transendothelial migration (TEM) capacity

Specifically, at the beginning of the Results section, we have clarified the rationale for using VM as a phenotypic marker to identify tumor cell populations with potentially enhanced TEM capacity. We have also explicitly acknowledged the limitation regarding the mechanistic link between VM and cancer cell entry into the circulation in the Discussion section. The following statement has been added at the end of the second paragraph of the Discussion: "Notably, VM has been widely used as a morphological feature associated with aggressive tumor behavior. We therefore employed it as a phenotypic screening marker to identify subpopulations with enhanced TEM capacity, which subsequently led us to identify GLUD1 as a key regulator of this process. It should be emphasized that the GLUD1-VM correlation is associative, and we do not claim a mechanistic connection between VM structures and metastasis. Rather, our study focuses on the role of GLUD1 in regulating TEM-related tumor cell behavior, whereas the mechanistic basis of VM formation and its causal contribution to metastasis remain beyond the scope of the current work. "

We believe these revisions clarify the intended use of VM in our study and address the reviewer's concern by explicitly distinguishing VM as a morphological screening marker from the mechanistic regulation of TEM by GLUD1.

3. My purpose is to help the authors to improve their work and for readers to understand their findings with logic rationales. If the authors could in the manuscript text further clarify these issues, this reviewer is happy to support publication of this interesting work.

Response: We are grateful to the reviewer for the constructive and encouraging comments, as well as for the valuable time and effort devoted to evaluating our manuscript. We appreciate the reviewer's goal of helping us improve the logical clarity and interpretability of our findings.

As detailed in our responses to Points 1 and 2 above, we have revised the manuscript text accordingly. Specifically, we have:

1. Clarified the rationale and limitations of the zebrafish assay and provided additional experimental evidence using intervitelline space injection to support our conclusions.

2. Explicitly stated that VM was employed solely as a morphological screening marker to identify tumor cells with potentially enhanced TEM capacity. We have also acknowledged that the mechanistic link between VM and cancer cell invasiveness remains unresolved and is beyond the scope of the current study.

We believe that these revisions have substantially improved the logical clarity of our manuscript and will help readers more accurately interpret the scope and implications of our findings. We sincerely hope that the revised manuscript now meets the reviewer's expectations. Once again, we thank the reviewer for the constructive and supportive feedback.

Reviewer #2

Overall, the author has addressed most of my concerns, thanks. However, several major concerns still require serious attention and further resolution.

Response: We sincerely thank the reviewer for the positive acknowledgment and for the continued constructive feedback. We have taken all remaining concerns very seriously and have addressed each point as detailed below. In particular, we have further strengthened the concentration-related evidence by performing absolute quantification of nuclear α-KG, adding dose-response analyses in a purified recombinant protein system, clarifying the interpretation of extracellular prodrug concentrations, and excluding endogenous α-KG interference in the key concentration-dependent IP assays. All newly generated data have now been incorporated into the revised manuscript as main or supplementary figures and cited in the relevant sections.

1. For Major Comment 1, the author stated that cellular α-KG concentrations in the tested cell lines were measured to range from 50 to 500 μM, and these data should be explicitly presented. In addition, IP data for the critical control group (0 μM α-KG treatment) and other concentration groups (10, 1000, and 5000 μM) should be supplemented in Figure A5. Furthermore, it remains unclear whether endogenous α-KG was eliminated prior to the IP experiments. If endogenous α-KG was not removed, its potential interference and influence on the experimental results should be

thoroughly discussed.

Response: We thank the reviewer for this important suggestion. We have now explicitly presented the absolute α-KG concentration data in the tested cell lines in the revised manuscript. Because the GLUD1/SNAIL interaction investigated in this study occurs in the nucleus, we measured and reported nuclear α-KG concentrations, which ranged from approximately 50 to 500 μM across the tested cell lines. These data are now shown in **Figure B2**.

Unpublished Data Figure Redacted

Figure B2. Absolute nuclear α-KG concentration (μM) of PC9/OR, A549/DTX, A549 and PC9 cell lines. All data are represented as mean ± SEM.

In addition, we have supplemented the IP data for the critical control 0 μM α-KG control group and a broader range of α-KG concentrations, including 0.05, 0.5, 1, 10, 50, 100, 500, and 1000 μM, to generate a full dose-response panel. Importantly, these IP experiments were performed in a cell-free purified recombinant protein system rather than in cell lysates. Therefore, endogenous α-KG was not present in this assay system, eliminating potential interference from endogenous α-KG. As shown in **Figure B3**, increasing concentrations of α-KG progressively promoted GLUD1/SNAIL complex formation, with 100 μM identified as the threshold concentration for promoting ternary complex assembly. This concentration falls within the measured nuclear α-KG concentration range in the tested cell lines, supporting the physiological relevance of the observed effect. It is noteworthy that in the High TEM cell line, which demonstrates enhanced complex formation, the nuclear concentration of a-KG is elevated to a level sufficient to effectively facilitate complex assembly.

The data presented in **Figures B2 and B3** have now been incorporated into the revised manuscript as Figure 4K-4L.

Unpublished Data Figure Redacted

Figure B3. α-KG promotes GLUD1/SNAIL complex formation in a purified recombinant protein system. (A) Representative immunoblot showing GLUD1/SNAIL complex formation in the presence of increasing concentrations of α-KG: 0, 0.05, 0.5, 1, 10, 50, 100, 500, and 1000 μM. (B) Quantification of the immunoblot results. Arrows indicate the measured absolute nuclear α-KG concentrations in A549/DTX, A549, PC9, and PC9/OR cells.

2. For Major Comment 4, I appreciate the author's detailed response. Nevertheless, I am somewhat surprised that the nuclear α-KG concentration remains consistent after these complex experimental procedures. Consistent with the suggestion for Major Comment 1, the author should quantify the absolute nuclear α-KG concentration rather than only analyzing its relative content. This quantification is essential, as it allows readers to directly evaluate the assembly of the ternary complex induced by different α-KG concentrations.

Response: We thank the reviewer for this insightful comment. We fully agree that absolute quantification of nuclear α-KG concentration is essential for directly evaluating the concentration-dependent assembly of the ternary complex.

In our initial response to Major Comment 4, we described the experimental details for nuclear α-KG detection. We performed absolute quantification of nuclear α-KG using LC-QTRAP/MS. Authentic α-KG standards were used to generate an external calibration curve, and 2-ketoglutaric acid-$^{13}C_5$ was included as an isotopically labeled internal standard to correct for potential variations during sample extraction and MS detection. The nuclear α-KG concentration was calculated based on the measured α-KG amount normalized to the estimated nuclear fraction volume, as detailed in the Methods section.

The absolute nuclear α-KG concentrations in different cell lines, as well as the concentration changes following GLUD1 genetic intervention or exogenous dimethyl-α-KG supplementation, are now presented in **Figure B4**. These data allow direct comparison with the α-KG concentration range used in the purified protein IP assay and support the conclusion that physiologically relevant nuclear α-KG concentrations promote GLUD1/SNAIL ternary complex assembly.

Unpublished Data Figure Redacted

Figure B4. Absolute nuclear α-KG concentrations (μM) of PC9/OR, PC9, A549/DTX, A549, siGLUD1-transfected A549/DTX cells, oeGLUD1-transfected A549 cells, and A549/DTX cells treated with 10 μM dimethyl-α-KG.

3. For Major Comment 5, sequence alignment analyses across different species are missing for the key residues presented in Figures 5f and 5j.

Response: We thank the reviewer for this valuable suggestion. In our previous revision, we only analyzed the sequence conservation of the α-KG-binding residue K147 in GLUD1. Following the reviewer's recommendation, we have now extended the sequence alignment analysis to include all key residues presented in Figures 5F and 5J, including GLUD1 residues Y2, Y4, R13, I132, and V156 and SNAIL residues P2, F5, and R8. As shown in **Figure B5**, these residues are highly or largely conserved across vertebrate species, supporting the evolutionary conservation of the residues involved in the α-KG/GLUD1/SNAIL interaction. The sequence alignment data have now been incorporated into the revised manuscript as Supplementary Fig. 7.

Unpublished Data Figure Redacted

Figure B5. Cross-species conservation of key residues involved in the α-KG/GLUD1/SNAIL interaction. (A) Cross-species conservation of the α-KG-binding residue K147 in GLUD1. (B-C) Cross-species conservation of GLUD1 (B) and SNAIL (C) residues contributing to the GLUD1-SNAIL interface.

4. For Major Comments 6 and 7, dimethyl-α-KG and diethyl glutarate were used at a concentration of 10 μM in this study. The author claimed that these two compounds can be metabolized into α-KG and glutarate, respectively. However, it is critical to verify whether 10 μM exogenous α-KG or glutarate addition is sufficient to induce significant activation or inhibition of the ternary complex. Moreover, it is unclear whether cells can actively uptake these two compounds, which may lead to a substantial discrepancy between intracellular and extracellular compound concentrations. Additionally, the removal of endogenous α-KG during the IP experiments needs to be confirmed.

Response: We thank the reviewer for raising this important concern. We agree that it is essential to determine whether 10 μM extracellular dimethyl-α-KG or diethyl glutarate is sufficient to alter intracellular metabolite levels and thereby affect ternary complex assembly.

It should be noted that the 10 μM concentration refers to the extracellular concentration of esterified prodrugs, rather than the intracellular concentration of the corresponding metabolites. Dimethyl-α-KG and diethyl glutarate are cell-permeable esterified derivatives that can enter cells and be hydrolyzed by intracellular esterases to release α-KG and glutarate, respectively. Because

the hydrolyzed products are charged and cannot freely diffuse back across the plasma membrane, they undergo metabolic trapping, leading to sustained intracellular accumulation at concentrations that can substantially exceed the extracellular concentration[4]. Consistent with this mechanism, our LC-QTRAP/MS measurements confirmed that treatment with 10 μM dimethyl-α-KG significantly elevated nuclear α-KG levels from 115.9 μM to 370.3 μM, corresponding to a 3.19-fold increase (**Figure B6**). According to our dose-response results shown in Figure B4, this nuclear α-KG range is sufficient to promote GLUD1/SNAIL ternary complex assembly. For diethyl glutarate, although nuclear glutarate concentrations were not directly quantified in the same manner, the esterified prodrug design supports efficient cellular uptake and intracellular hydrolysis via the same metabolic trapping mechanism as dimethyl-α-KG. In addition, the significant biological changes observed in Supplementary Fig. 8C and 8D support that 10 μM diethyl glutarate was sufficient to exert functional effects under our experimental conditions.

Regarding the potential interference of endogenous α-KG during IP experiments, our study employed both cell-based IP assays and the purified recombinant protein system. In the cell-based IP assays, endogenous α-KG was not experimentally removed, and these experiments were used to assess the interaction under cellular conditions. In contrast, the concentration-dependent IP assays used to evaluate the direct effect of α-KG on ternary complex assembly were performed in a purified recombinant protein system, which is free from endogenous α-KG interference. Specifically, the IP results presented in Figures 4K, 4Q, and 4R were derived from the purified recombinant protein system. Therefore, the observed α-KG-dependent regulation of GLUD1/SNAIL complex formation is not confounded by endogenous α-KG present in cell lysates.

Unpublished Data Figure Redacted

Figure B6. Absolute nuclear α-KG (μM) concentrations in A549/DTX cells with or without 10 μM dimethyl-α-KG treatment, n = 3. All data are represented as mean ± SEM.

5. For Major Comment 9, no data regarding D/L 2-HG is provided in Figure A10.

Response: We thank the reviewer for pointing out this omission. To address this concern, we have now performed additional Co-IP assays using purified proteins and in vitro ChIP assays in the presence of D-2-HG and L-2-HG. As shown in **Figure B7**, treatment with 100 μM D- or L-2-HG only weakly promoted the GLUD1-SNAIL protein-protein interaction, and the magnitude of this

effect was substantially lower than that of α-KG at the same concentration. Consistently, D/L-2-HG only marginally increased the occupancy of the GLUD1/SNAIL complex at the CDH5 promoter, reaching approximately 9% (L-2-HG)/ 22.8% (D-2-HG) of the effect observed with α-KG. Together with our previous analysis of other TCA-cycle intermediates (glutamate, succinate, malate, and fumarate), these results confirm that the promoting effect on GLUD1-SNAIL complex formation is largely specific to α-KG. Furthermore, we also examined whether D-2-HG or L-2-HG exerts a competitive inhibitory effect on α-KG, similar to that observed with glutarate, in the in vitro ChIP assays. Interestingly, D-2-HG and L-2-HG, like fumarate, showed a modest synergistic effect rather than a competitive inhibitory effect. Data for all tested metabolites, including glutamate, succinate, malate, fumarate, D-2-HG and L-2-HG, have now been incorporated as Supplementary Fig. 6O-6R in the revised manuscript.

Unpublished Data Figure Redacted

Figure B7. Effects of D-2-HG and L-2-HG on GLUD1–SNAIL complex formation and promoter binding. (A) Co-IP analysis showing the effects of L-2-HG and D-2-HG, relative to α-KG, on GLUD1–SNAIL complex formation in a purified protein system. All metabolites were tested at 100 µM. (B) Effects of L-2-HG and D-2-HG on GLUD1/SNAIL binding to the CDH5 promoter in an in vitro ChIP assay. All metabolites were tested at 100 µM. Data were normalized to input. n = 6. All data are represented as mean ± SEM.

6. For additional suggestions, all newly acquired experimental data should be appropriately displayed in either the main figures or supplementary materials of the manuscript.

Response: We thank the reviewer for this suggestion. All newly acquired data generated in response to the reviewer's comments have now been incorporated into the revised manuscript, either in the main figures or in the supplementary materials, and have been cited in the relevant sections of the manuscript. Specifically, the full dose-response panel for α-KG-promoted GLUD1-SNAIL interaction has been added as Fig. 4K-4L (in response to Major Comment 1); the Coomassie blue-stained SDS-PAGE gels of purified GLUD1 and SNAIL proteins have been included as Supplementary Fig. 6K (in response to Major Comment 2); the cross-species sequence conservation of key residues involved in complex formation has been presented in Supplementary Fig. 7 (in response to Major Comment 5); and the effects of α-KG-related metabolites have been shown in

Supplementary Fig. 6O-6R (in response to Major Comment 9).

Reviewer #3

The authors have addressed most of the concerns raised during the initial review. However, the new data generated and presented in the response to the review do not appear to have been incorporated into the revised manuscript; these data should be included.

Response: We thank the reviewer for this helpful suggestion. All newly acquired data generated in response to the reviewers' comments have now been incorporated into the revised manuscript, either in the main figures or in the supplementary materials and have been cited in the relevant sections of the manuscript.

In response to Comment 1, GLUD1 knockdown using a second siRNA and its effect on TEM ability have been shown in Supplementary Fig. 1I-1K.Importin-8 knockdown using a second siRNA and its effect on GLUD1 nuclear translocation have been shown in Supplementary Fig. 4H-4I. The effect of GLUD1 knockdown on nuclear complex formation in another cell line, such as PC9/OR, has been shown in Supplementary Fig. 5I. The broader relevance of the protein complex across additional cancer types has been presented in Supplementary Fig. 8E-8H.

In response to Comment 2, the effects of SNAIL and VE-Cadherin intervention in GLUD1-high and GLUD1-low cells have been shown in Fig. 2F-2H, Supplementary Fig. 3M-3O, and Supplementary Fig. 5J-5O.

In response to Comment 3, the subcellular distribution ratio of GLUD1 has been shown in Supplementary Fig. 4E-4F, Supplementary Fig. 4H-4I.

In response to Comment 4, GLUD1 expression in CTCs has been shown in Supplementary Fig. 5R.

In response to Comment 5, the R162-dependent enzymatic activity of GLUD1 has been shown in Supplementary Fig. 6L.

In response to Comment 6, the effects of α-KG-related metabolites have been shown in Supplementary Fig. 6O-6R.

Reviewer #4 (Remarks to the Author):

The authors have fully addressed all concerns raised in my initial review by presenting rigorous, well-designed experimental data, comprehensive analyses, and targeted textual revisions. The manuscript has seen substantial improvements in mechanistic depth, technical robustness, and presentation clarity. Accordingly, I recommend acceptance of this manuscript for publication in Nature Communications.

Below is an optional minor suggestion for final polishing, which is not required for acceptance:

1. Regarding the pan cancer correlation between GLUD1 expression and vasculogenic mimicry (VM) signatures, I recommend that the authors briefly discuss in the Discussion section the potential factors contributing to the distinct correlation strengths observed across lung cancer, breast cancer,

and melanoma. Such a discussion would further strengthen the translational implications of the present study.

Response: We thank the reviewer for this valuable suggestion. We appreciate the reviewer's recommendation to further discuss the distinct correlation strengths between GLUD1 expression and VE-Cadherin/CDH5 across different cancer types.

In the revised manuscript, we have added a brief analysis of the clinical relevance of the GLUD1/VE-Cadherin axis across multiple cancer types using TCGA datasets, with the following description included in the final result section: "Additionally, we further explored the clinical relevance of the GLUD1/VE-Cadherin axis across multiple cancer types using TCGA datasets. Notably, GLUD1 expression positively correlated with VE-Cadherin gene levels in high-VM breast cancer and melanoma cohorts (Supplementary Fig. 8E-8F). Moreover, VE-Cadherin expression was significantly elevated in advanced-stage tumors (stage III/IV) compared with early-stage tumors (stage I/II), supporting a potential role for this pathway in other malignancies, as we showed in NSCLC models (Supplementary Fig. 8G-8H)." These data have now been included as Supplementary Fig. 8E-8H in the revised manuscript.

In addition, we have added a brief discussion of the potential reasons underlying the variable correlation strengths across cancer types in the Discussion section, as follows: "The variable correlation strengths between GLUD1 expression and VE-Cadherin across lung cancer, breast cancer, and melanoma may be partly attributable to the nature of bulk TCGA transcriptomic data, which may be influenced by differences in tumor purity, vascular density, stromal composition, endothelial cell abundance, and immune infiltration across cancer types. In addition, cancer type-specific molecular backgrounds, metabolic dependencies and baseline propensities may further contribute to the variable correlation patterns observed among different malignancies. Thus, while our data suggest that the GLUD1/VE-cadherin axis may have broader translational relevance beyond NSCLC, the applicability and clinical value of these pan-cancer correlations in other cancer types require further systematic investigation."

We believe these additions address the reviewer's suggestion by providing both correlative data and a cautious biological interpretation, which we hope will help readers better assess the translational relevance of the GLUD1/VE-Cadherin axis across different tumor types.

Reference

1. Roth, S. M., Berens, E. B., Sharif, G. M., Glasgow, E. & Wellstein, A. Cancer Cell Invasion and Metastasis in Zebrafish Models (Danio rerio). *Methods Mol Biol* **2294**, 3–16 (2021).
2. Teng, Y. *et al.* Evaluating human cancer cell metastasis in zebrafish. *BMC Cancer* **13**, 453 (2013).
3. Loveless, R., Shay, C. & Teng, Y. Unveiling Tumor Microenvironment Interactions Using Zebrafish Models. *Front Mol Biosci* **7**, 611847 (2020).

4. Parker, S. J. *et al.* Spontaneous hydrolysis and spurious metabolic properties of α-ketoglutarate esters. *Nat Commun* **12**, 4905 (2021).

Reviewer #1 The authors have satisfactorily addressed my concerns and the manuscript should be accepted for publication in NC.

Response: We sincerely thank the reviewer for the positive assessment of our revised manuscript and for recommending its acceptance for publication in Nature Communications. We are also grateful for the reviewer's constructive comments throughout the review process, which have been instrumental in improving the rigor, clarity and overall quality of the manuscript.

Reviewer #2 The authors have addressed all of my concerns. I now believe this manuscript meets the requirements for publication in Nature Communications.

Response: We greatly appreciate the reviewer's confirmation that all concerns have been satisfactorily addressed and that the manuscript now meets the requirements for publication in Nature Communications. We sincerely thank the reviewer for the insightful suggestions provided during the review process, which have helped us substantially strengthen and refine the manuscript.
